# Photocontrolled Energy Storage in Azobispyrazoles with Exceptionally Large Light Penetration Depths

**DOI:** 10.1021/jacs.2c07537

**Published:** 2022-10-12

**Authors:** Alejandra Gonzalez, Magdalena Odaybat, My Le, Jake L. Greenfield, Andrew J. P. White, Xiang Li, Matthew J. Fuchter, Grace G. D. Han

**Affiliations:** † Department of Chemistry, Brandeis University, 415 South Street, Waltham, Massachusetts 02453, United States; ‡ Molecular Sciences Research Hub, Department of Chemistry, Imperial College London, White City Campus, 82 Wood Lane, London W12 0BZ, U.K.

## Abstract

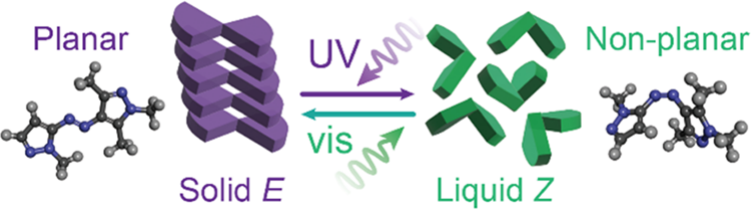

Azobispyrazole, 4pzMe-5pzH, derivatives with small terminal substituents (Me, Et, *i*-Pr, and *n*-Pr) are reported to undergo facile reversible photoswitching in condensed phases at room temperature, exhibiting unprecedentedly large effective light penetration depths (1400 μm of UV at 365 nm and 1400 μm of visible light at 530 nm). These small photoswitches exhibit crystal-to-liquid phase transitions upon UV irradiation, which increases the overall energy storage density of this material beyond 300 J/g that is similar to the specific energy of commercial Na-ion batteries. The impact of heteroarene design, the presence of *ortho* methyl substituents, and the terminal functional groups is explored for both condensed-phase switching and energy storage. The design principles elucidated in this work will help to develop a wide variety of molecular solar thermal energy storage materials that operate in condensed phases.

## Introduction

Molecular solar thermal (MOST) energy storage compounds that undergo light-induced reversible isomerization have been explored for optically controlled thermal energy storage and release. Particularly, their application as solar energy collectors, whereby MOST compounds harness solar irradiation in the daytime and release thermal energy on demand after sunset, has been pursued^1,2^ to provide active energy load shifting^3^ for energy-efficient buildings. A variety of photoswitches, such as norbornadiene–quadricyclane,^4^ dihydroazulene–vinylheptafulvene,^5^ fulvalene diruthenium,^6,7^ and azobenzene,^8−10^ have been investigated, with an aim to increase their isomerization enthalpy, Δ*H*_iso_, through covalent functionalization and thereby achieve a greater energy storage density. In addition to Δ*H*_iso_, the absorption profiles, quantum yields, thermal reverse-isomerization temperatures (*T*_iso_), and thermal half-lives (*t*_1/2_) of metastable isomers are key and give an indication of a photoswitch’s potential as a MOST material.

Recently, the design of MOST compounds that undergo a phase transition (PT-MOST) from solid to liquid during light irradiation has emerged, which enables the storage of latent heat, in addition to Δ*H*_iso_, upon the photoisomerization of the switches. This design strategy has been widely employed to achieve a MOST system that operates in condensed phases^11^ and accomplishes a gravimetric energy density over 300 J/g for practical applications;^12^ 300 J/g, or 83 Wh/kg, is within the range of specific energies of commercial 18650-size Na-ion batteries and two to three times lower than those of Li-ion batteries.^13^ However, the translation of the typically measured solution-state properties to the condensed phase is often not straightforward: the molecular packing in a crystalline phase substantially limits the conformational freedom of the molecules and often translates to suboptimal conversion of isomers under light irradiation.^14^ In addition, the light penetration depth through condensed-phase materials is significantly reduced as compared to that of dilute solutions, due to the increased optical density of photochromic materials. The effective light penetration depth for condensed-phase MOST compounds has been experimentally probed for only a few azobenzene derivatives, ranging from 1 to 2 μm^15^ (at 365 nm) to 100–350 μm (at 530 and 590 nm),^16^ depending on the substitution pattern on the azobenzene scaffold. The larger effective penetration depths were achieved by the *ortho*-functionalization of azobenzene core with methoxy (108 μm, measured for 590 nm irradiation) and fluorine (349 μm for 530 nm) groups. The negligible absorption of the incident light by the generated isomer leads to the deeper penetration of light and the successful isomerization of thicker samples.^16^ However, achieving effective light penetration depths in the hundreds of μm range remains a bottleneck for the application of MOST compounds in thick films or large-scale devices. We note that for the purpose of this study, the term “effective penetration depth” is used to mean the maximum sample thickness for complete conversion.

Following growing understanding of their structure–property relationships,^17^ many derivatives of azoheteroarene switches have been recently developed,^18^ incorporating various heteroarenes such as pyrazole,^19−22^ triazole,^23−25^ tetrazole,^17^ pyrrole,^17,26^ imidazole,^27,28^ and isoxazole.^29,30^ Studies on the PT-MOST properties of azoheteroarene compounds have been rarely conducted, except for arylazopyrazole derivatives with long alkyl functional groups.^31−33^ Designing photoswitchable azoheteroarenes to enable condensed-phase switching with appropriate phase transitions is a key challenge to overcome in realizing efficient PT-MOST compounds with high energy densities. In this work, we present the design principles of azobispyrazole switches that allow for a reversible phase transition at room temperature upon light irradiation, a substantial heat storage (>300 J/g), and an exceptionally large effective light penetration depth of UV (>1400 μm) and visible light (>1400 μm). The design principles discovered here will guide the future research of other azoheteroarene PT-MOST compounds, opening up the opportunities to rationally generate a diverse suite of compounds for energy storage applications.

**Figure 1:**
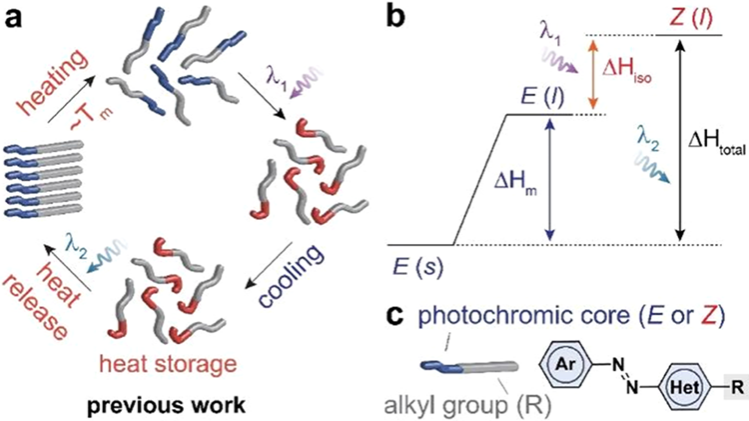
(a) Previous design of the optically controlled latent heat storage and release cycle of PT-MOST, which requires the initial melting of *E* isomers prior to the photoisomerization.^16,31,34^ See Figure 2a for the new design that allows for photoswitching and concurrent phase transition at room temperature, enabled by the compact molecular design with *ortho*-substituents and without long alkyl groups. Reprinted with permission from ref (^31^). Copyright 2020 American Chemical Society. (b) Energy diagram for PT-MOST compounds that store both latent heat and isomerization energy upon photoisomerization. Reprinted with permission from ref (^11^). Copyright 2022 American Chemical Society. (c) A general structure of PT-MOST compounds that are previously studied.^16,32,33^

Figure 1a shows a general schematic of energy storage and release in PT-MOST compounds, as demonstrated in the previous work of our group^16,31,32^ and others.^35−37^ Since the initial *E* isomers form a crystalline phase that hinders the configurational change of photoswitches upon irradiation, the *E*-rich material is typically melted prior to photoactivation at λ_1_. The resulting *Z* isomers form a stable liquid phase within a wide range of temperatures (e.g., −40 °C to *T*_iso_),^31^ which is then irradiated at λ_2_ to switch to the *E* isomers, crystallize and release the stored energy. The total energy storage (Δ*H*_total_) in PT-MOST compounds is illustrated in Figure 1b as the combination of the *E–Z* isomerization energy (Δ*H*_iso_) and the latent heat of *E* isomers (Δ*H*_m_). We note that all enthalpy terms are experimentally obtained by differential scanning calorimetry (DSC). Unlike thermally driven phase transitions, the photoinduced phase transitions are under photodynamic equilibrium^38^ where Δ*G* is not 0. Thus, the quantification of entropy contribution to the phase transition is not viable.

In general, the established molecular design of reported PT-MOST compounds incorporates a photochromic core, including various azobenzene and arylazopyrazole derivatives, and an alkyl group that ranges from hexyl to pentadecyl that are linked to the core via an ether^33,39^ or ester group^16,31^ (Figure 1c). The long alkyl chains contribute to increasing Δ*H*_m_ per molecule (in kJ/mol) while increasing the melting points of compounds and lowering the gravimetric energy density (in J/g). The high melting points of some compounds, exceeding *T*_iso_ of their *Z* isomers, crucially limit the *E*-to-*Z* photoisomerization due to the competing thermal reversion (*Z*-to-*E*) during the photoirradiation of the molten *E* isomers. The low gravimetric energy density can be addressed through the design of PT-MOST compounds with low molecular weights. A recent work reported by our group investigated a series of compact azobenzene PT-MOST compounds with a small functional group, replacing R, and demonstrated a potential to achieve gravimetric energy density over 300 J/g.^34^ However, due to the short half-lives of (∼2 days) the azobenzene derivatives, it remains a challenge to store latent heat for a long period of time.

We have previously studied the alkyl-functionalized arylazopyrazoles as PT-MOST materials,^31,32^ which are developed from arylazopyrazole switches characterized with increasingly long *Z* isomer half-lives from 10 days to 74 days, to 1000 days, to 46 years.^17,19^ These values correspond to the storage of a decreasing amount of Δ*H*_iso_ from 49 kJ/mol to 38 kJ/mol, 36 kJ/mol, and 30 kJ/mol. Thus, to achieve Δ*H*_iso_ larger than 49 kJ/mol while maintaining a reasonable stability of *Z* isomers for diurnal heat storage, the desired half-lives of *Z* isomers should range from a few days up to 10 days. Concurrently, the optical properties of switches should exhibit near-quantitative photostationary state (PSS) ratios, i.e., near 100% conversion, for both UV and vis light irradiations in solution, which enhances the switchability of compounds in condensed phases and the light penetration depth as well. In addition, the design of low-molecular-weight PT-MOST compounds is crucial for achieving a desired level of gravimetric energy storage density over 300 J/g.

## Results and Discussion

### Molecular Designs

**Figure 2:**
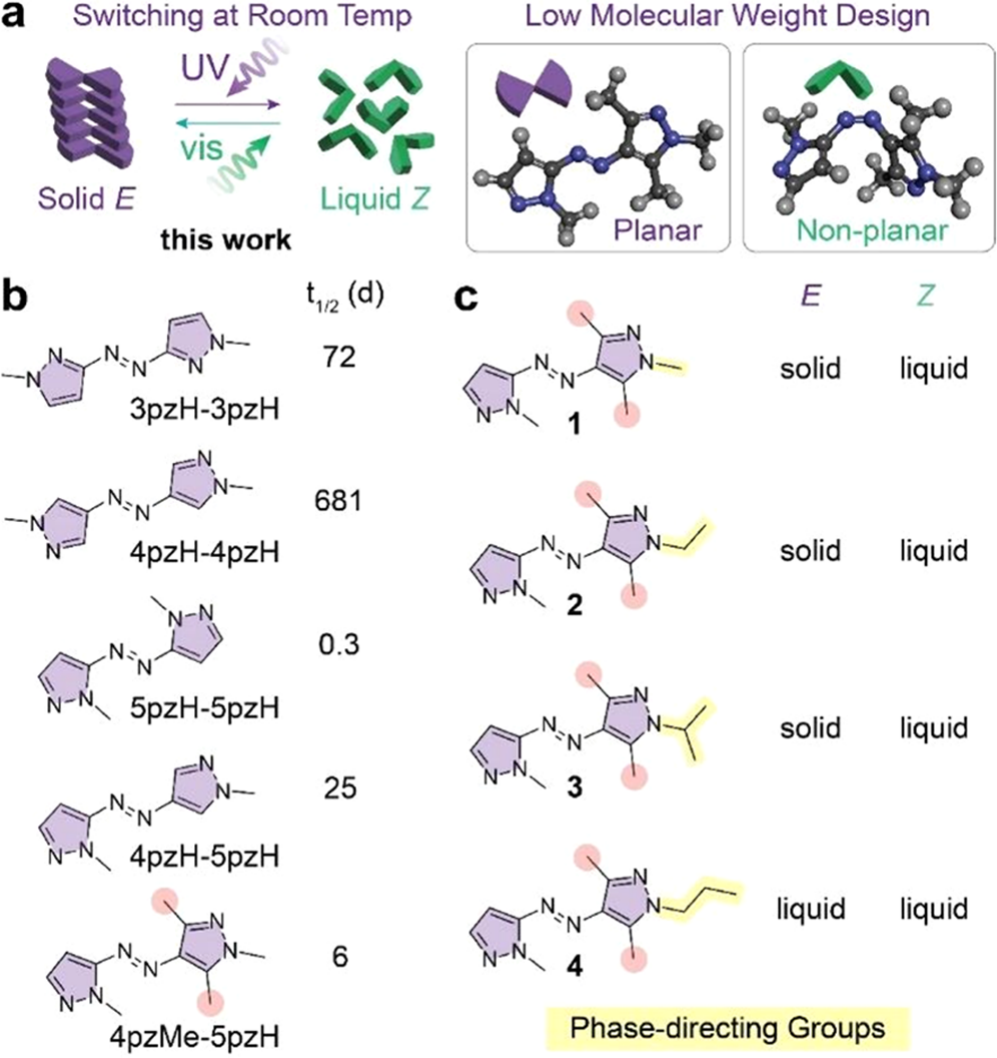
(a) Schematic of UV- and visible light-induced isomerization, phase transition, and isomeric structures of azobispyrazoles. (b) Structures and *Z* isomer half-lives of reported azobispyrazoles^20^ and newly designed 4pzMe-5pzH. (c) Chemical structures of four azobispyrazoles studied in this work, highlighting the phase-directing groups on the identical photochromic core (4pzMe-5pzH), and their phase in each isomeric form.

To identify switches that meet these criteria and undergo reversible *E–Z* isomerization and an appropriate phase transition at room temperature, we elected to study the azobispyrazoles (Figure 2a). It should be highlighted that azo switches bearing two heteroaromatic rings are currently unexplored as MOST materials. We surveyed an array of azobispyrazole designs, including three symmetrical derivatives (3pzH-3pzH, 4pzH-4pzH, and 5pzH-5pzH) very recently reported by Li et al.,^20^ all of which exhibited suboptimal half-lives given the analysis above: 72 days, 681 days, and 0.3 day, respectively. Among the potential alternative candidates, we identified an azobispyrazole, 4pzH-5pzH, that was found to have a *Z* isomer half-life of 25 days^20^ (Figure 2b). Previously, we have demonstrated the degree of heteroaromatic ring substitution adjacent to the azo group to significantly affect the thermal isomerization kinetics for a number of azoheteroarene switches,^17^ with increased steric bulk adjacent to the azo group destabilizing the *Z* isomer leading to more rapid thermal *Z*-*E* relaxation. [Note, we refer to substitution at this position as “*ortho*” in analogue to other *ortho*-substituted azobenzenes]. Therefore, to further adjust the half-life of 4pzH-5pzH to below 10 days, we incorporated two *ortho* methyl groups on the 4pz ring. The resultant compound, 4pzMe-5pzH, was characterized to have the ideal optical and thermal properties: 98% PSS ratio for both UV (365 nm) and visible light (525 nm) irradiation due to the excellent spectral separation between *E* and *Z* isomers (Figure S7), high quantum yields associated with both the forward (Φ*_EZ_* 0.33) and backward (Φ*_ZE_* 0.60) photoisomerization processes (Figures S10–S12, Table S1), a *Z* isomer half-life of 6 days (Figure S13), and high fatigue resistance with no sign of fatigue over 10 switching cycles (Figure S14).

Following the selection of 4pzMe-5pzH as the optimal photochromic core, the terminal alkyl group on the nitrogen of the 4pzMe ring was varied to probe its impact on the molecular packing of *E* and *Z* isomeric forms (Figure 2**c**). The impact of a small functional group on azobenzene core on the condensed-phase switching was demonstrated in our previous work^34^ where the variation of the small substituents drastically influenced the yield of photoswitching in condensed phases, the stability of the liquid phase, and the total energy storage density. The results elucidated the following effective design principles: a small, nonplanar functional group (e.g., methoxy and ethoxy) on a planar photochromic core successfully induces the facile photoswitching of azobenzene at room temperature in solids and leads to the formation of a *Z* liquid that is stable at various temperatures. Inspired by the findings, we varied the functional group on the nitrogen of the 4pzMe ring among methyl (**1**), ethyl (**2**), isopropyl (**3**), and *n*-propyl (**4**) substituents. Since the gravimetric energy density of PT-MOST systems is critically dependent on the molecular weight of MOST compounds, the substituent was limited to or smaller than C_3_H_7_ (43.1 g/mol, less than 18% of the molecular weight). The precursor of the derivatives, 4pzMe-5pzH with a free N-H group, was not considered in this study due to its short thermal half-life of 1.5 h (Figure S15).

Variation of the terminal alkyl group resulted in switches with comparable properties. The ethyl (**2**), isopropyl (**3**), and *n*-propyl (**4**) analogues exhibited thermal half-lives of 5.9, 6.2, and 6.7 days, respectively (Figures S16–S18): very similar thermal isomerization kinetics to the parent N-methyl analogue **1** (*t*_1/2_ = 6.0 days, Figure S13). The excellent photoswitch characteristics (quantitative bidirectional photoswitching (Figure S7), high quantum yields (Table S1), and fatigue resistance (Figure S14) for both photoisomerization processes) were also retained.

### Phases of Compounds and Their Changes under Photoirradiation

**Figure 3:**
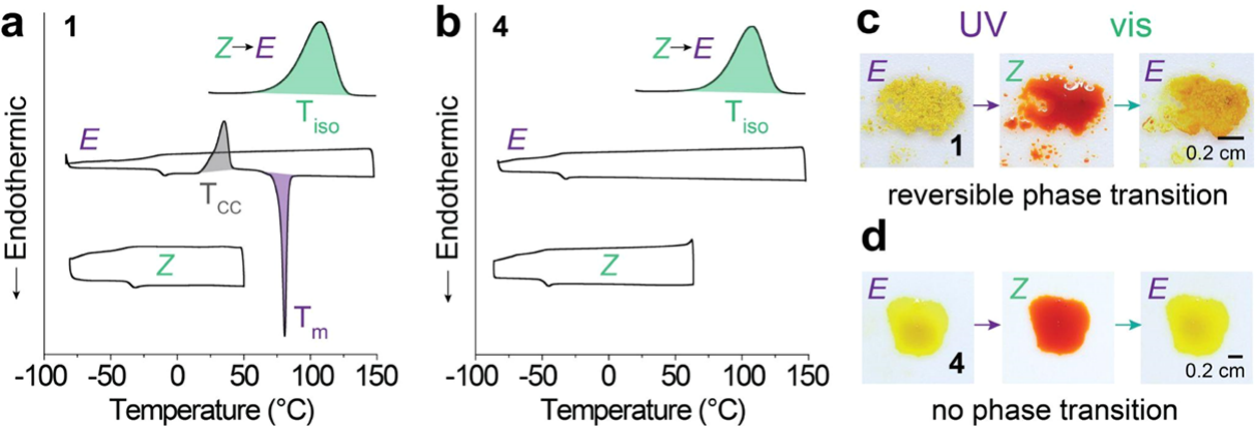
(a) DSC plots of *E* and *Z* isomers of compound **1** and the thermal reversion of *Z* to *E* isomer. (b) DSC plots of *E* and *Z* isomers of compound **4** and the thermal reversion of *Z* to *E* isomer. (c) Optical images of UV- and visible light-induced reversible phase transition of compound **1**. (d) Optical images of UV- and visible light-induced isomerization of compound **4** in liquid.

The thermal properties of compounds **1** and **4** are illustrated in Figure 3a,b, respectively. The *E* isomer of compound **1** undergoes melting and subsequent supercooling to −90 °C, and then the supercooled liquid cold-crystalizes upon being reheated above room temperature. The *Z* isomer, on the other hand, exhibits a stable liquid state from −30 to 50 °C, due to its nonplanar structure. This indicates the potential of the compounds to store energy over a wide range of temperatures and enable thermal energy storage applications in both cold and hot climates. The *Z* isomer upon heating above 60 °C undergoes thermal reversion to the *E* isomer, releasing the isomerization energy (Δ*H*_iso_). Therefore, the UV-activated compound **1** stores both Δ*H*_iso_ and Δ*H*_m_ in its *Z* isomeric form, and compounds **2** and **3** behave similar to the *E* and *Z* isomers (Figure S19). In contrast, compound **4** with an *n*-propyl functional group on the nitrogen of the 4pzMe ring displays a stable liquid phase for both *E* and *Z* isomeric states. Therefore, the total energy storage is limited to Δ*H*_iso_, in the absence of latent heat storage, leading to the lowest energy storage in the series. Table 1 summarizes the thermal parameters of all compounds studied, and compounds **1–3** showed excellent gravimetric energy densities, close to or exceeding 300 J/g, due to their low molecular weights and the storage of both Δ*H*_iso_ and Δ*H*_m_.

**Table 1:** Thermal Properties of Compounds **1–4** in Their *E* Isomeric Forms and Their *Z*-to-*E* Isomerization Process

	*E*			*Z* → *E*				
	*T*_m_ (°C)	Δ*H*_m_ (kJ/mol)	Δ*H*_m_ (J/g)	*T*_iso_ (°C)	Δ*H*_iso_ (kJ/mol)	Δ*H*_iso_ (J/g)	Δ*H*_total_ (kJ/mol)	Δ*H*_total_ (J/g)
**1**	83	21	97	109	51	234	72	331
**2**	76	19	81	110	50	216	69	297
**3**	113	28	114	112	49	200	77	314
**4**	liq.	liq.	liq.	111	50	203	50	203

Parameters include melting point (*T*_m_), heat of fusion (Δ*H*_m_), peak isomerization temperature (*T*_iso_), isomerization enthalpy (Δ*H*_iso_), and the total energy storage density (Δ*H*_total_). For energy terms, both molar energy storage density (kJ/mol) and gravimetric density (J/g) are shown.

Surprisingly, compounds **1–3** underwent a solid-to-liquid phase transition upon UV irradiation at room temperature, despite the high melting temperatures of their *E* isomers (Table 1). Many of the previously reported *E* isomers of azo(hetero)arene derivatives required heating near to, or above, their melting points, which increases conformational freedom of molecules, to undergo photoisomerization in condensed phases.^16,31,34^ Therefore, the rare ability to photoisomerize in the crystalline phase and to produce liquid phase of *Z* isomers at room temperature, far below the melting points of *E* isomers, is recognized and indicates the unique crystal packing of the *E* isomers (*vide infra*). We note that the light sources used in the condensed-phase switching experiments are of low irradiance; we chose LEDs (2.1 mW/cm^2^ at 365 nm and 0.95 mW/cm^2^ at 530 nm) that closely represent the level of solar irradiance at the given wavelengths (0.36 mW/cm^2^ at 360 nm and 1.3 mW/cm^2^ at 530 nm; ASTM G-173–031 reference,^16^ 10 nm bandwidth, Figure S20). This allows us to predict the isomerization behavior and phase transition kinetics of the compounds, upon the exposure to solar irradiation (1 Sun) through bandpass filters that select the desired range of wavelengths. This is in contrast to previously reported experiments where high-irradiance lamps were used (80 mW/cm^2^ at 365 nm and 60 mW/cm^2^ at 450 and 420 nm)^36,37^ to facilitate rapid isomerization of azobenzene derivatives in the condensed phase.

Figure 3c shows the crystalline powder of compound **1** (*E* isomer) photoswitching to the *Z* isomer and melting simultaneously under the UV irradiation over 70 min, reaching about an 80% *Z*-rich PSS (Figure S21). The *Z*-rich liquid undergoes a complete reversion to a crystalline solid of *E* isomers, when exposed to 530 nm light for 20 min. Compounds **2** and **3** also exhibit reversible phase transitions (Figures S22 and S23), while the UV irradiation time required to achieve a similar level of PSS varied: 45 min for compound **2** and 140 min for compound **3** with a comparable sample amount of 3 mg and thickness (∼100 μm). This variation is attributed to the lower and higher *T*_m_ and Δ*H*_m_ of compounds **2** and **3**, respectively, compared to those of compound **1**, indicating the varied level of intermolecular interactions between the *E* isomers of each compound. Compound **4** exhibits no phase transition upon photoirradiation (Figure 3d) and a 95% *Z*-rich PSS (Figure S24) in the liquid phase within 45 min. X-ray diffraction of the *E* and *Z* isomers of each compound (Figure S25) corroborates the phase transition observed by the DSC and optical images.

### Static and Effective Light Penetration Depth Studies

**Figure 4:**
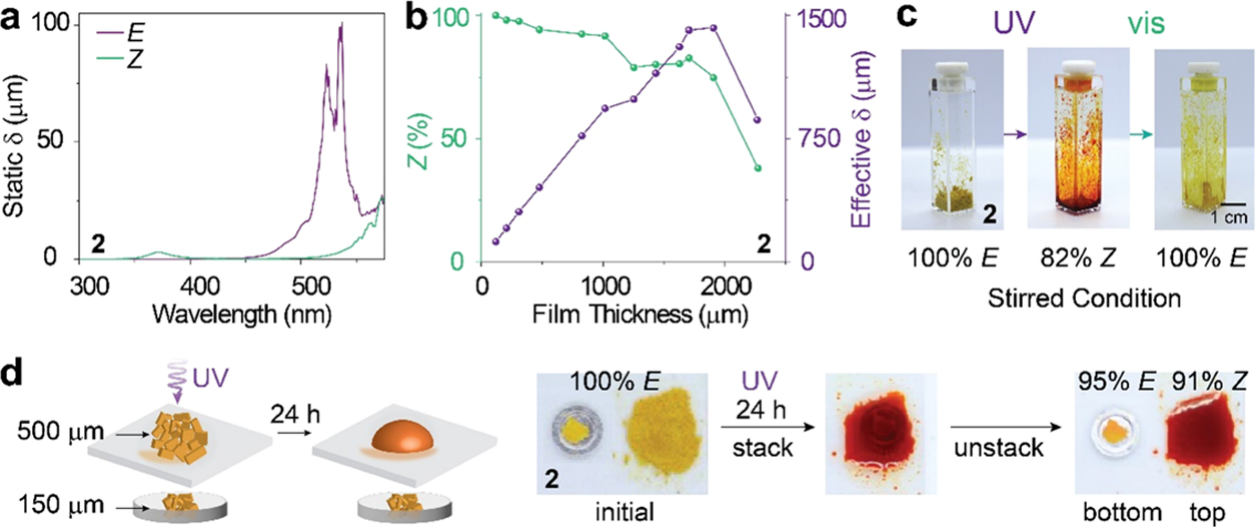
(a) Static penetration depth δ calculation of compound **2**. (b) *E*-to-*Z* conversion yield under 365 nm irradiation (left, green line) and the measured 365 nm penetration depth δ (right, purple line) at different film thicknesses of compound 2-*E*. The effective penetration depth was experimentally determined by the multiplication of film thickness and the *E-*to-*Z* conversion yield. (c) Optical images of compound 2 in bulk undergoing reversible isomerization and phase transition under the stirred condition. (d) A schematic of the molecular convection test and optical images acquired from experiments. Glass slides (2.5 cm × 2.5 cm × 0.1 cm^3^) were stacked atop aluminum pans (0.5 cm diameter × 330 μm thickness).

Compound **2**, which undergoes the most rapid isomerization and phase transition under UV irradiation, was selected to investigate the light penetration depth of the 4pzMe-5pzH scaffold, which determines the potential of the photoswitches for large energy storage applications. We first calculated the static light penetration depth (δ) of the *E* and *Z* isomers in condensed phases^35^ based on their extinction coefficient measured by UV–vis absorption spectroscopy (Figure 4a). In the absence of diffusion and convection, the maximum penetration depth of 365–375 nm through the *Z* isomer is ∼3 μm, and that of 530–540 nm through the *E* isomer is ∼100 μm. The static light penetration depth calculations of other compounds are shown in Figure S26. Then, we evaluated the effective light penetration depths of the *E* and *Z* isomers through the condensed-phase irradiation experiments. The pressed *E* powder sample thickness was varied from 100 to 2300 μm, and the samples were irradiated with a 365 nm LED (2.1 mW/cm^2^) for 24 h (Figures S27–S34). The liquefied samples were analyzed by ^1^H NMR to measure the *E*-to-*Z* conversion yield (Figure 4b). Remarkably, the effective UV penetration depth, calculated by the multiplication of the conversion yield and the initial *E* film thickness, culminated in 1400 μm. In contrast, a previously studied azobenzene-tethered polymer^15^ shows only 1–2 μm of light penetration at 365 nm, despite 24-h irradiation by a much stronger UV lamp (21.7 mW/cm^2^).

Compound **2** displays a continuously raised δ as the film thickness increases until the thickness is greater than 1700 μm. We hypothesize that the gradual liquefaction of compounds, the solvation of remaining *E* isomers by *Z* liquid, and the molecular diffusion and convection in the liquid contribute to increasing the *E*-to-*Z* conversion yield even in thick samples. When the film thickness exceeds 2000 μm, the *E*-to-*Z* conversion decreases significantly, only reaching ∼38% within 24 h of UV irradiation. We hypothesize that the molecular convection is limited in the thickest sample (∼2300 μm). As compared to the previously studied photochromes with long alkyl chains or their polymerized forms, the azobispyrazoles with small functional groups are anticipated to allow for a more facile molecular convection in liquid, due to the reduced van der Waals interactions, which is speculated to enhance the δ. The *Z*-to-*E* reverse photoisomerization, promoted by the irradiation at 530 nm, was greater than 96.6% within 300 min of exposure, except for the thickest sample (∼2300 μm) that required 540 min of irradiation for the reversion greater than 86%. We have determined the largest effective penetration depth of 530 nm to be ∼1400 μm based on the 1705 μm film that was able to switch from 82.5% *Z* to 0% *Z* within 300 nm of irradiation. This breaks the record (349 μm penetration depth at 530 nm) of a previously studied PT-MOST that was irradiated by the identical 530 LED (0.95 mW/cm^2^) for 3 h.^16^ The thick samples were irradiated with 365 and 530 nm repeatedly, showing a consistent level of isomerization and phase transition over three cycles (Figure S35). Encouraged by the exceptionally large effective penetration depths measured for compound **2**, we performed a large-volume phase transition experiment, as shown in Figure 4c. The powder of compound **2**-*E* of ∼115 mg was irradiated by UV while being stirred in a cuvette, which represents an artificial convection condition, and the *E*-to-*Z* conversion was monitored over 62 h. The PSS ratio over 82% *Z* was achieved within 24 h, and the complete photoreversion to *E* isomers was obtained in ∼7 h (Figures S36–S38).

To confirm the impact of spontaneous molecular diffusion and convection on the achievement of large effective penetration depth, we devised an experiment (Figure 4d) where two *E* solid samples were separated by a glass substrate (100 μm thick) that prevents physical mixing between them. After 24 h of UV exposure, the top layer (∼500 μm) was photoliquefied, and 91% of *E*-to-*Z* conversion was achieved, which again verifies its large effective penetration depth that is far greater than the static depth (∼3 μm) of UV. In contrast, the bottom layer remained largely intact, showing only 5% photoconversion, due to the UV attenuation by the upper layer and the lack of molecular convection. We performed a control experiment where the bottom layer was covered only by a glass substrate (Figure S39), which confirmed that the glass substrate did not hinder the photoliquefaction of *E* solids (∼100 μm; 94% *Z* achieved). We note that photothermal heating, particularly by UV irradiation, is prominent, which may contribute to the facilitated molecular convection of *Z* isomers. We monitored a ∼2 °C increase in the temperature around the sample (Figure S40) upon UV irradiation, during the *E*-to-*Z* isomerization, using an IR camera. This temperature change is significant, compared to the negligible change observed during the irradiation at 530 nm. The heat released from the *Z*-to-*E* isomerization and concurrent crystallization of ∼40 mg sample, estimated to be ∼12 J, did not contribute to any detectable temperature change. This is attributed to the rapid heat dissipation from the sample to the environment, despite the presence of thermal insulation around the sample by styrofoam.

We hypothesize that the exceptionally facile photoliquefaction of *E* isomers with high T_m_ and the large effective penetration depths are attributed to our molecular design bearing the *ortho* methyl substituents on the 4pzMe and 5pzH rings. The crystal structures of compounds **1** and **3** are shown in Figures S41 and S42. Compound **1** displays a staggered stacking among the photochromes (Figure S43); 4pzMe rings are separated from each other and primarily stack with 5pzH rings, presumably due to the stronger steric repulsion between the 4pzMe rings bearing two *ortho* methyl groups. Compound **3** primarily exhibits van der Waals interactions between the isopropyl chains, leaving the aromatic cores well separated (Figure S44). Both the *ortho* methyl substituents and the additional isopropyl group are speculated to contribute to increasing the conformational freedom of photoswitches in the crystal. The role of *ortho* methyl substituents in enabling the crystal-phase photoswitching and liquefaction was further investigated by comparing a series of arylazopyrazoles and their ability to switch in crystals. Previously reported arylazopyrazoles 4pzMe, 5pzH, and 4pzH^17^ that contain two, one, and zero *ortho* methyl groups, respectively, on the photochrome were irradiated with UV at room temperature for 24 h to induce *E*-to-*Z* isomerization and liquefaction (Figure S45). We used two types of UV lamps (UVA with λ_max_ of 351 nm and UVB with λ_max_ of 312 nm) with broad emission bands (315–400 and 280–380 nm) instead of 365 nm LED to test their behavior under common UV sources. Only 4pzMe switched and liquefied under both UV irradiations, and 4pzH did not undergo photoliquefaction; 5pzH underwent photoliquefaction only by the irradiation of UVA that is more strongly absorbed by 5pzH. From this experiment, we suggest that the methyl substituents near the azo group enhance isomerization upon irradiation, whereas the N-methyl group on the 4pzH ring, pointing away from the azo group, has a lesser impact. Another example of UV-induced liquefaction of arylazo-3,5-dimethylisoxazoles, reported by Venkataramani et al.,^30^ corroborates our hypothesis.

## Conclusions

In summary, we have discovered that azobispyrazoles with *ortho* methyl functional groups exhibit extremely facile photoswitching between crystalline *E* and liquid *Z* at room temperature, storing over 300 J/g of energy for MOST applications. Most notably, the switches with small substituents, instead of conventionally used long alkyl chains, displayed an effective light penetration depth over 1400 μm, which is attributed to the facile photoliquefaction and solvation of *E* isomers. The design strategies of PT-MOST compounds that can store a substantial amount of energy in a thick device of condensed liquid phase for a long period of time will be further explored to develop optimal MOST compounds based on azoheteroarenes.

## Supporting Information Available

Experimental details; synthetic procedures; NMR spectra of the *E* and *Z* isomers of compounds **1**–**4**; UV–vis spectra; quantum yield measurements; thermal isomerization kinetics; DSC plots; photoliquefaction experiments; bulk sample experiments; and crystal structures of compounds **1** and **3** (PDF)

## Notes

CCDC 2165362 and 2165363 contain the supplementary crystallographic data for this article. These data can be obtained free of charge via www.ccdc.cam.ac.uk/data_request/cif, or by emailing data_request@ccdc.cam.ac.uk, or by contacting The Cambridge Crystallographic Data Centre, 12 Union Road, Cambridge CB2 1EZ, U.K.; fax: +44 1223 336033.
