# Supplementary material for: Photocontrolled Energy Storage in Azobispyrazoles with Exceptionally Large Light Penetration Depths
Source: J Am Chem Soc. 2022 Oct 12;144(42):19430–6. doi: 10.1021/jacs.2c07537 (PMC9619401; doi:10.1021/jacs.2c07537)
Supplement: Supplementary file 1 — ja2c07537_si_001.pdf [file ja2c07537_si_001.pdf]

Photo-Controlled Energy Storage in Azobispyrazoles with Exceptionally Large Light Penetration Depths

Alejandra Gonzalez,[a,#] Magdalena Odaybat,[b,#] My Le,[a] Jake L. Greenfield,[b] Andrew J.P. White,[b] Xiang Li,[a] Matthew J. Fuchter,[b,*] and Grace G. D. Han[a,*]

[a] Department of Chemistry, Brandeis University, 415 South Street, Waltham, MA, 02453, USA

[b] Molecular Sciences Research Hub, Department of Chemistry, Imperial College London, London W12 0BZ, U.K.

[#] These authors contributed equally.

Email: gracehan@brandeis.edu, m.fuchter@imperial.ac.uk

SUPPORTING INFORMATION

Table of Contents

1. General Methods 3

Synthesis 3

UV-Vis Absorbance Spectroscopy 3

Photoswitching 4

Quantum Yield Measurements 4

Thermal Isomerization Kinetics 5

Differential Scanning Calorimetry (DSC) 5

Preparation of *Z* isomer Samples for DSC Measurement 6

Thin Film Preparation for Powder Charging 6

Thin Film Preparation for Penetration Depth Studies [thickness <450 μm] 7

Pan Preparation for Penetration Depth Studies [thickness >450 μm] 7

Thin Film Preparation for UV-Vis Measurements in Condensed Phase 8

Bulk Sample *E*-*Z* Isomerization 9

Photoliquefaction Experiments of Arylazopyrazole Switches 4pzMe, 5pzH and 4pzH 9

Emission Spectra of LCZ-UVA and LCZ-UVB Lamps, LEDs 10

Powder Diffraction Measurements 13

Supplementary Notes 13

2. Synthetic Route and Procedures 15

3. NMR Analysis of *E* and *Z* isomers of Compounds 1-4 21

4. UV-Vis Absorption Spectra 30

5. Measuring Quantum Yields 32

6. Thermal Isomerization Kinetics and Fatigue Resistance 35

7. Differential Scanning Calorimetry Plots of Compounds 2 and 3 41

8. Solar Irradiance Measurement 42

9. Photoinduced Reversible Phase Transition of Compounds 1-4 43

10. X-Ray Diffraction Measurements 47

11. Penetration Depth Measurements 48

12. Profilometer Measurements 51

13. Bulk Sample Isomerization 57

14. Additional Photoliquefaction Results 59

15. Crystal Structures 60

16. Photoliquefaction of Arylazopyrazoles 4pzMe, 5pzH and 4pzH 64

17. References 65

1. General Methods

Synthesis

All reactions were performed in the dark. Reagents and solvents were obtained from commercial sources and used as supplied unless otherwise stated. Reactions were monitored by thin-layer chromatography (TLC) using Merck silica gel 60 F254 plates (0.25 mm). TLC plates were visualized using UV light (254 nm) and/or by using the appropriate TLC stain. Column chromatography was performed on Merck Silica Gel 60 (230–400 mesh) with technical grade solvents used as supplied. ^{1}H spectra were recorded on a Bruker Avance 400 spectrometer at 400 MHz. Chemical shifts (δ) are reported in ppm (parts per million) referenced to residual solvent signals: 7.26 ($CHCl_3$), 1.94 (MeCN). Coupling constants (*J*) are reported in hertz (Hz). All ^{13}C spectra were recorded on a Bruker Avance 400 spectrometer at 101 MHz. Chemical shifts (δ) are reported in ppm referenced to solvent signals: 77.16 ($CDCl_3$). High-resolution mass spectra (ESI, APCI) were recorded by the Imperial College London Department of Chemistry Mass Spectroscopy Service using a Micromass Autospec Premier and Micromass LCT Premier spectrometer.

UV-Vis Absorbance Spectroscopy

UV-Vis absorption spectra of compounds **1-4** were obtained on a Cary 60 UV-Vis spectrophotometer equipped with a temperature controller or a Cary 50 Bio UV-Vis spectrophotometer in a UV Quartz cuvette with a path length of 10 mm. Solutions of the compounds were prepared in HPLC or higher-grade solvents at a concentration of 25 μM. The condensed-phase UV-vis absorption spectra of all the compounds were recorded with the same

spectrophotometer using thin, sandwiched films of dimensions 2.5 cm × 2.5 cm × 0.1 cm (VWR Vistavision Microscope Slides).

Photoswitching

Solution-state samples were irradiated with a custom-built irradiation set up using 365 nm (3 × 800 mW Nichia NCSU276A LEDs) and 525 nm (3 × 450 mW NCSG219B-V1 LEDs) light sources. Condensed-phase samples were irradiated with other LED light sources including Thorlabs M365LP1 (365 nm, 21.0 µW/mm^2, 2000 mW) and M530L3 (530 nm 9.5 µW/mm^2, 370 mW). We note that different sets of LEDs were used for solution-state and condensed-phase measurements, due to the nature of collaboration conducted between two groups. The FWHM bandwidths of the green LEDs are significant (38 nm for 525 nm LED; 35 nm for 530 nm LED), which enables the comparable effect on photoswitching. The bandwidths of the 365 nm LEDs are smaller: 9 nm for both LED types. To verify that PSS at each wavelength was reached, the sample was irradiated until no change in spectrum was observed either by NMR or UV/vis spectroscopy. For all compounds NMR or UV/vis measurements were taken immediately after irradiation. The PSS compositions were characterized by NMR using 20 mM solution in MeCN-d_3 (Section 3).

Quantum Yield Measurements

Ferrioxalate actinometry was used to determine the photon flux from a Nichia NCSU276A 365 nm LED (365 nm, 800 mW @ 100% power) operating at 5% power, fitted with a collimating lens, following a previously reported procedure.[1] The cuvette was placed 3.5 cm away from the light source. The photon flux of the 365 nm LED operating at 5% power for our setup was determined to be 1.845×10^{16} photons/s. The photon flux from a Nichia NCSG219B-V1 525 nm LED (525 nm,

450 mW @ 100% power) operating at 5% power, fitted with a collimating lens, was determined using Aberchrome 670 as a reference. Initially, a solution of Aberchrome 670 in toluene was converted to the ring-closed form by irradiation with 365 nm light. This solution was then placed 3.5 cm away from the 525 nm light source. The photon flux of the 525 nm LED operating at 5% power for our setup was determined to be 1.248×10^{16} photons/s.

The quantum yield of photoisomerization (Φ) for the switches under 365 and 525 nm light were determined using previously reported literature procedure and the calculation was performed using their provided software.[1]

Thermal Isomerization Kinetics

Thermal *Z*-*E* isomerization kinetics were followed by UV-vis spectroscopy. For 4pzMe-5pzH-H the thermal isomerization kinetics was measured at 25 °C. For all other switches the thermal isomerization kinetics was measured at a range of elevated temperatures that allowed determination of the kinetics at 25 °C via extrapolation using Eyring plots. The measurements were performed in an air-tight cuvette, at 25 μM concentration using DMSO as the solvent. In the beginning each sample was converted to the *Z*-rich PSS and the absorbance was then recorded at a certain time interval. Between measurements the sample was kept inside the instrument at a constant temperature in the absence of ambient light.

Differential Scanning Calorimetry (DSC)

DSC analysis was conducted on a DSC 250 (TA Instruments) with an RCS 90 cooling component. All samples were weighed using a TGA Q500 (TA Instruments) and heated at a rate of 10 °C/min

unless otherwise noted. All *E* isomers were melted and cooled to -90 °C before reheating. In DSC, the *Z* isomers were heated below their respective T_{iso} to prevent *Z*-to-*E* reversion. To determine the ΔH_{iso} of *Z* isomers, samples were heated from 20 °C until the thermal isomerization was completed.

Preparation of *Z* isomer Samples for DSC Measurement

Z isomers of compounds **1-4** were obtained by dissolving the *E* isomers in acetonitrile and irradiating the sample with 365 nm LED for 24 hours until a photostationary state was reached. *Z*-rich samples were concentrated, dried under high-vacuum, and taken for NMR to confirm the percentage of *E*-*Z* conversion. Samples were then transferred to DSC pans for analysis.

Thin Film Preparation for Powder Charging

Thin-film samples of compounds **1-4** were prepared by adding 3 mg of the *E* isomer on a clean glass slide (2.5 cm × 2.5 cm). Photoisomerization of powder film samples of compounds **1-3** and liquid film samples of compound **4** were performed by the following procedure. The films were directly irradiated with 365 nm LED at 1000 mA until films appeared to completely photoliquefy (compounds **1-3**), or for 45 minutes (compound **4**). NMR samples were prepared in dark and taken to determine the *E*-*Z* photoisomerization content.

Discharging of liquid phase films of compounds **1-4** was performed by irradiating the samples with 530 nm LED for 5 to 15 minutes at room temperature until the color changed from dark red-

orange to dark yellow. The samples were then taken for NMR spectra using deuterated acetonitrile to determine the percentage of *Z*-*E* conversion.

Thin Film Preparation for Penetration Depth Studies [thickness <450 μm]

E isomers of compound **2** were first heated on a hot plate at 100 °C. The melted samples were pipetted dropwise on a clean glass slide (2.5 cm × 2.5 cm). The thickness of the *E*-rich droplets were then measured using the Zeta-20 Optical Profilometer. Ten measurements were taken over different portions of the samples, which were then averaged and represent film thickness. The samples were then directly irradiated with 365 nm UV light for 24 hours and the percentage of conversion was determined by ^{1}H NMR using deuterated acetonitrile as solvent.

The discharge of the *Z*-rich films was conducted by irradiating the samples with 530 nm light until the PSS of *Z*-*E* photoisomerization was achieved. The percentage of conversion was also determined by ^{1}H NMR using deuterated acetonitrile as solvent.

Pan Preparation for Penetration Depth Studies [thickness >450 μm]

Profilometer was first used to determine the thickness of empty aluminum DSC pans. The solid *E* isomers of compound **2** were broken apart and packed into the empty DSC pans. The pans were then covered with lids and pressed to create a uniform surface. After the lids were taken off, the DSC pans with uniform sample were taken to the profilometer to determine their thickness. Ten measurements were taken over different portions of the samples, which were then averaged and represent film thickness. The thickness of the sample was calculated by subtracting the value of

the filled pan from the empty one. The samples were then directly irradiated with 365 nm UV light for 24 hours and the percentage of conversion was determined by ^{1}H NMR in deuterated acetonitrile.

The discharge of the *Z*-rich films was conducted by irradiating the samples with 530 nm light until the PSS of *Z*-*E* photoisomerization was achieved. The percentage of conversion was determined by ^{1}H NMR.

Thin Film Preparation for UV-Vis Absorption Measurements in Condensed Phase

The thin film preparation was performed with 5 mg of crystalline compound **1-3** with each being melted on a clean glass slide (2.5 cm x 2.5 cm) on a hot plate at high temperature above their melting points. 5 mg of the liquid compound **4** was obtained by dissolving 2 mg of the compound in 0.2 mL DCM and drop-casting 0.05 mL of the solution on the hot plate until the solvent was totally evaporated. All the thin films were then covered with another clean glass slide for the compounds spread to the entire glass substrate. The samples were then let to cool to room temperature. The sandwiched films were taken to the UV-Vis spectrophotometer to determine the *E* isomer absorption spectra in condensed phase.

The samples were then directly irradiated with 365 nm UV light for 72 hours before taken to the spectrophotometer to obtain the absorption spectra of solid-state *Z* isomer.

Bulk Sample *E*-*Z* Isomerization

The bulk sample isomerization was conducted with 115 mg of crystalline compound **2** in a UV quartz cuvette with a path length of 10 mm. The cuvette was placed on a piece of black paper and a stir plate (600 rpm) under 365 nm LED at 1000 mA at room temperature for 62 hours. The ratios of *Z* isomers were determined by ^{1}H NMR using deuterated acetonitrile as solvent.

The bulk discharge was performed by irradiating the charged sample with 530 nm LED under identical conditions for 430 minutes. The ratios of converted *E* isomers in the sample were also determined by ^{1}H NMR using deuterated acetonitrile as solvent. Digital photos were taken by Canon EOS REBEL SL2 200D.

Photoliquefaction Experiments of Arylazopyrazole Switches 4pzMe, 5pzH and 4pzH

The photoliquefaction experiments were performed with small quantities (~2 mg) of the crystalline compounds **4pzMe**, **5pzH** and **4pzH** on a glass slide. The samples were irradiated with UVA or UVB light for 24 h in a Luzchem LZC-4V photoreactor, fitted with either 10×8 W LZC-UVA (emission band 315–400 nm, λ_{max} 351 nm) or 10×8 W LZC-UVB (emission band 280–380 nm, λ_{max} 312 nm) lamps, respectively.

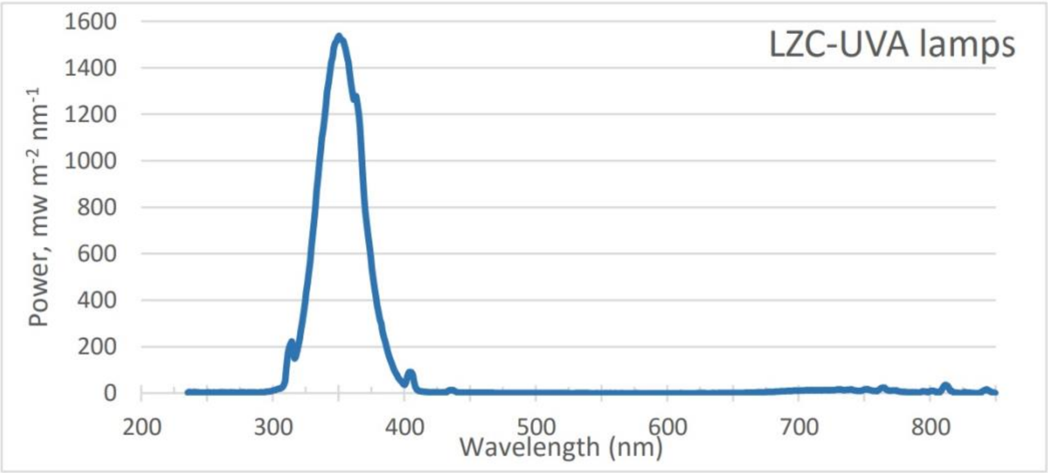

Figure S1. Emission spectrum of LZC-UVA lamps.[2]

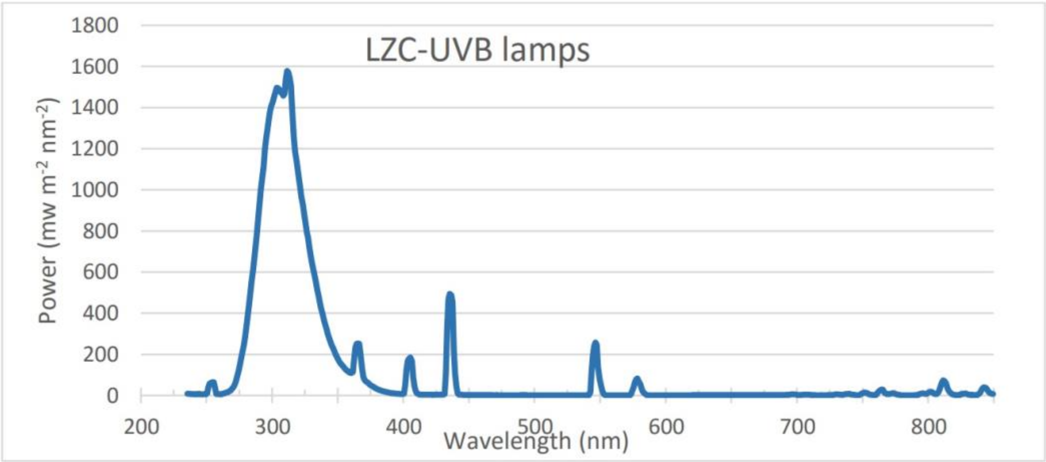

Figure S2. Emission spectrum of LZC-UVB lamps.[2]

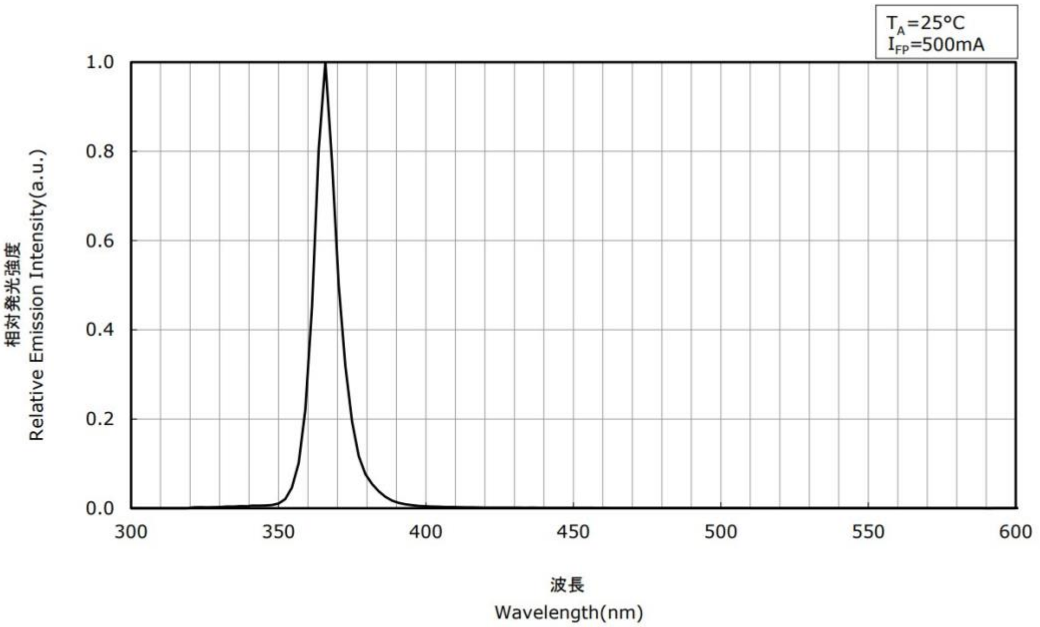

Figure S3. Emission spectra of 365 nm (800 mW Nichia NCSU276A LED) light source.[3]

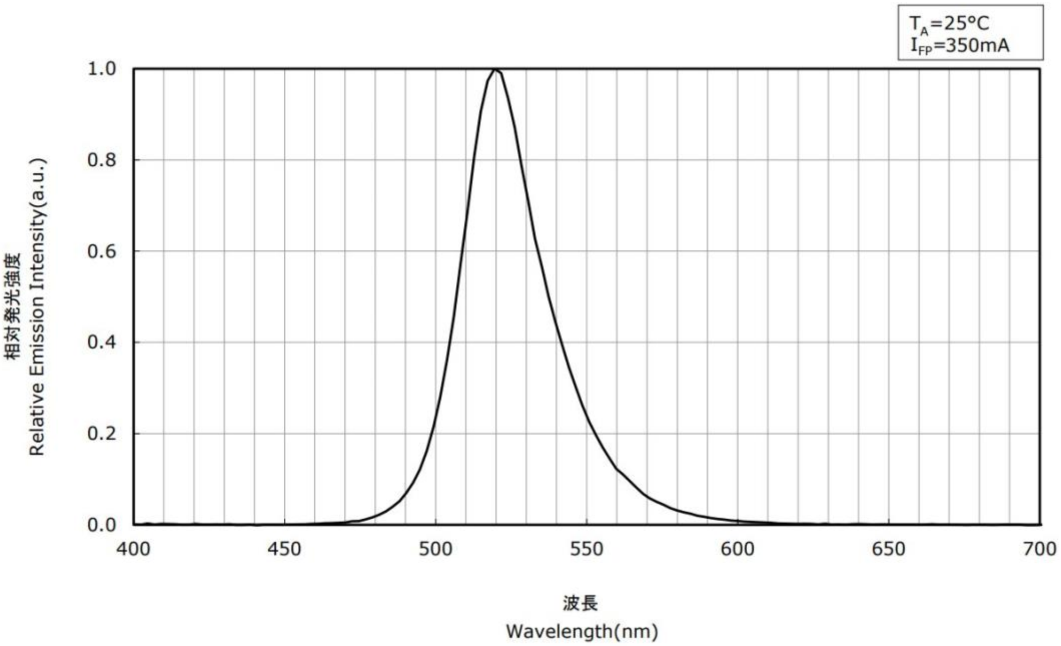

Figure S4. Emission spectra of 525 nm (450 mW NCSG219B-V1 LED) light source.[4]

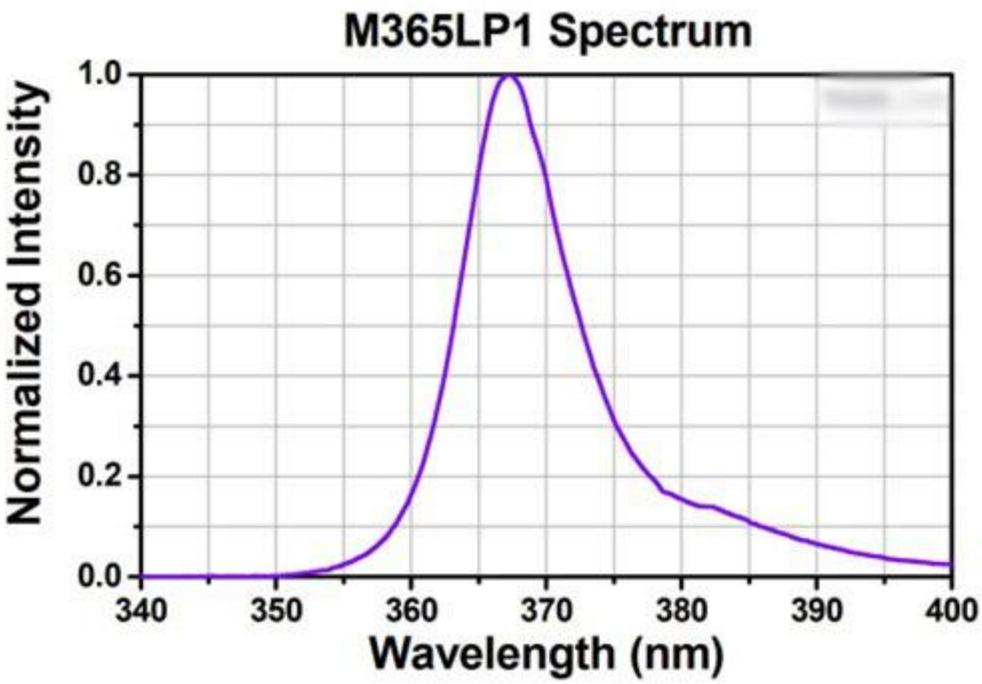

Figure S5. Emission spectra of 365 nm (Thorlabs M365LP1) light source.[5]

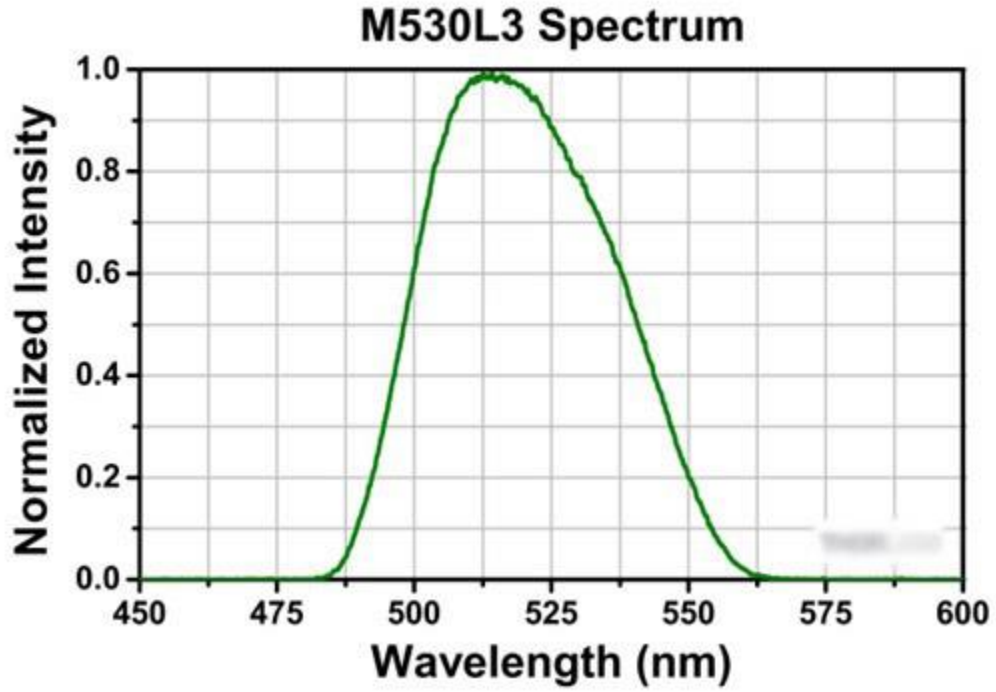

Figure S6. Emission spectra of 530 nm (Thorlabs M530L3) light source.[5]

Powder Diffraction Measurements.

X-ray powder diffraction in the 2θ range 0-40° (step size, 0.014°; time/step, 20 s; 0.04 rad soller; 40 mA × 60 kV) was collected on a PANalytical Empyrean diffractometer equipped with an GaliPIX3D line detector and in Bragg-Brentano geometry, using Mo-Kα radiation (λ=0.7093187 Å) without a monochromator. Around 5 mg samples were loaded into capillary tubes (outer diameter = 0.7 mm) and the measurements were carried out on the capillary spinner.

Supplementary Note 1. Determination of Thermal Half-Life by the Eyring Equation

Thermal half-life of compounds at T = 298 K was extrapolated by use of the following equation:

$$ln\,\frac{k}{T} = \frac{-\Delta H}{R} \times \frac{1}{T} + ln\,\frac{k_B}{h} + \frac{\Delta S}{R}$$

k (rate constant); *T* (absolute temperature) *ΔH* (enthalpy of isomerization); *R* (gas constant); k_B (Boltzmann constant); *h* (Planck's constant); *ΔS* (entropy of isomerization). Rate constants at designated temperatures were fitted to a straight line using the equation above to obtain the rate constant at T = 298 K via extrapolation. The thermal half-life was then calculated using the following equation:

$$t_{1/2} = \frac{ln\,(2)}{k}$$

$t_{1/2}$ (thermal half-life); *k* (rate constant).

Supplementary Note 2. Determination of Effective Light Penetration Depth

The effective light penetration depth was determined using the following equation:

$$\delta = \frac{PSS_2}{PSS_1} \times 100 \times l$$

δ (effective light penetration depth); PSS_1 (max. %*Z* isomer at the photostationary state in a 121 μm thin film, 100% in our studies); PSS_2 (%*Z* isomer at the photostationary state reached in a test film); l (thickness of the test film). The effective light penetration depth was then calculated at each film that was incompletely switched (i.e. $PSS_2 < PSS_1$), then an averaged value was obtained.

Supplementary Note 3. Determination of Thickness of Samples Thicker Than 450 μm

Thickness of samples were determined by the following equation:

$$s = p_f - p_i$$

s (sample thickness); p_f (aluminum pan with compound); p_i (empty aluminum pan) Empty aluminum pans were initially measured by profilometer to determine their thickness prior to adding compound. Each sample was then measured using the profilometer, and ten measurements were taken over different portions of the samples, which were then averaged and represent film thickness. The thickness of the pan was subtracted from the thickness of the sample measured.

2. Synthetic Route and Procedures

The azobispyrazoles **1** – **4** were prepared following two- or three-step syntheses initiated with a diazonium coupling between aminopyrazole **5** and acetylacetone to afford the common diketone intermediate **6.** Then **6** was subjected to cyclisation condensation reactions with methylhydrazine or hydrazine to yield **1** and free N-H intermediate **7** respectively. Lastly, the free N-H intermediate **7** underwent a series of S_N2 reactions with EtI, ^{i}PrI and ^{n}PrI to afford compounds **2** – **4** respectively in good to excellent yields.

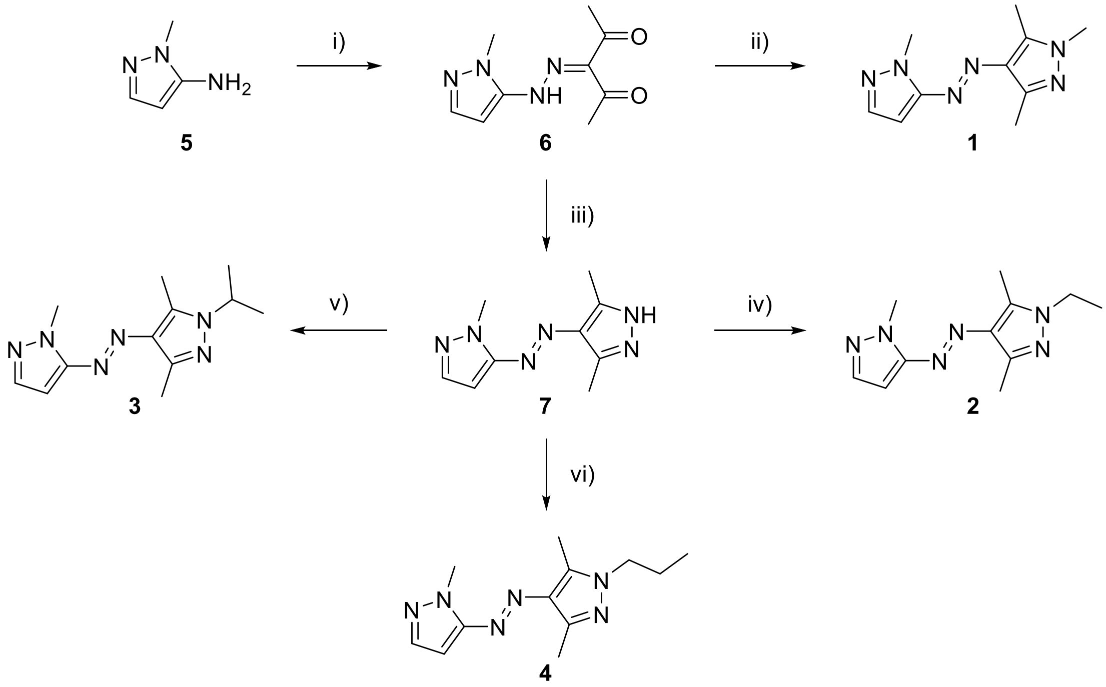

i) $NaNO_2$, HCl, H_2O, 0 °C, 45 min; then acetylacetone, NaOAc, EtOH, H_2O, 0 °C, 2 h, **29%**; ii) methylhydrazine, EtOH, 80 °C, 3 h, **47%**; iii) hydrazine, EtOH, 50 °C, 30 min, **83%**; iv) EtI, K_2CO_3, MeCN, 60 °C, 18 h, **82%**; v) ^{i}PrI, K_2CO_3, MeCN, 60 - 80 °C, 20 h, **50%**; vi) ^{n}PrI, K_2CO_3, MeCN, 60 °C, 18 h, **90%**.

Scheme S1. Synthetic route towards azobispyrazoles **1** – **4**.

3-(2-(1-methyl-1*H*-pyrazol-5-yl)hydrazineylidene)pentane-2,4-dione (6)

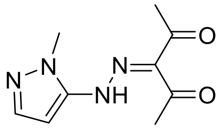

To the stirred solution of aminopyrazole **5** (2.91 g, 30.0 mmol) in water (60 mL) at 0 °C was added HCl (12 M, 14 mL) and the resulting mixture was stirred for 10 min. Prechilled solution of $NaNO_2$ (2.48 g, 36.0 mmol) in water (30 mL) was added dropwise and the resulting mixture was stirred at 0 °C for 45 min. Then the reaction mixture was added dropwise to a premixed suspension of acetylacetone (3.71 mL, 36.0 mmol) and NaOAc (14.8 g, 180 mmol) in EtOH (25 mL) and water (20 mL) resulting in the formation of a yellow precipitate. The pH was adjusted to ~7 by the addition of NaOAc and the reaction mixture was left stirring at 0 °C for 1 h. The precipitate was collected by vacuum filtration, washed with water (3x 30 mL) and the resulting filtrate was extracted with DCM (3x 200 mL). The combined organic fractions were dried over $MgSO_4$, filtered, concentrated under reduced pressure and purified by column chromatography eluting with hexane: EtOAc, 0 – 100%. The purified material was combined with the precipitate to afford compound **6** (1.81 g, 29% yield) as a yellow solid.

Rf 0.41 (hexane: EtOAc, 1: 1); ^{1}H NMR (400 MHz, $CDCl_3$) δ 14.94 (s, 1H), 7.43 (d, *J* = 2.1 Hz, 1H), 6.24 (d, *J* = 2.1 Hz, 1H), 3.91 (s, 3H), 2.62 (s, 3H); ^{13}C NMR (101 MHz, $CDCl_3$) δ 198.9, 196.8, 141.5, 139.1, 134.8, 94.8, 36.4, 31.8, 26.7; HRMS (ESI) *m/z* calc. for $C_9H_{13}N_4O_2$ $[M+H]^+$ 209.1039, found: 209.1037.

(*E*)-1,3,5-trimethyl-4-((1-methyl-1*H*-pyrazol-5-yl)diazenyl)-1*H*-pyrazole (1)

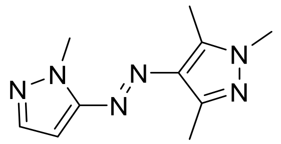

To the stirred solution of compound **6** (724 mg, 3.48 mmol) in EtOH (18 mL) at rt under nitrogen was added methylhydrazine (220 µL, 4.18 mmol). The resulting mixture was warmed to 80 °C and stirred for 3 h. Then the reaction mixture was cooled to rt and concentrated under reduced pressure. Purification by column chromatography eluting with hexane: EtOAc, 0 – 100% afforded compound **1** (355 mg, 47% yield) as a yellow solid.

Rf 0.17 (hexane: EtOAc, 1: 1); ^{1}H NMR (400 MHz, $CDCl_3$) δ 7.46 (d, *J* = 2.2 Hz, 1H), 6.37 (d, *J* = 2.2 Hz, 1H), 4.08 (s, 3H), 3.75 (s, 3H), 2.51 (s, 3H), 2.43 (s, 3H); ^{13}C NMR (101 MHz, $CDCl_3$) δ 154.2, 141.8, 140.3, 138.9, 135.8, 92.0, 36.1, 35.8, 14.4, 9.9; HRMS (ESI) *m/z* calc. for $C_{10}H_{15}N_6$ $[M+H]^+$ 219.1358, found: 219.1361.

(*E*)-3,5-dimethyl-4-((1-methyl-1*H*-pyrazol-5-yl)diazenyl)-1*H*-pyrazole (7)

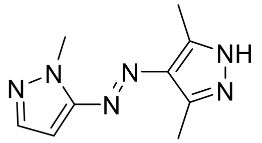

To the stirred solution of compound **6** (940 mg, 4.51 mmol) in EtOH (20 mL) at rt under nitrogen was added hydrazine (50 % in water, 310 µL, 4.97 mmol). The resulting mixture was warmed to 50 °C and stirred for 30 min. Then the reaction mixture was cooled to rt and concentrated under reduced pressure. Purification by column chromatography eluting with DCM: MeOH, 0 – 10 % afforded compound **7** (762 mg, 83 % yield) as a yellow solid.

Rf 0.49 (EtOAc); ^{1}H NMR (400 MHz, DMSO-d_6) δ 12.94 (s, 1H), 7.50 (d, *J* = 2.0 Hz, 1H), 6.36 (d, *J* = 2.0 Hz, 1H), 4.03 (s, 3H), 2.48 (s, 3H), 2.38 (s, 3H); ^{13}C NMR (101 MHz, DMSO) δ 153.4,

142.6, 139.3, 138.5, 134.9, 91.7, 35.6, 14.0, 10.1; HRMS (CI) *m/z* calc. for $C_9H_{13}N_6$ $[M+H]^+$ 205.1196, found: 205.1196.

(*E*)-1-ethyl-3,5-dimethyl-4-((1-methyl-1*H*-pyrazol-5-yl)diazenyl)-1*H*-pyrazole (2)

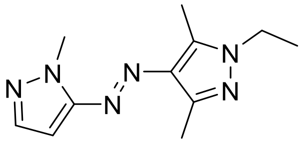

To a stirred solution of compound **7** (153 mg, 0.750 mmol) and K_2CO_3 (207 mg, 1.50 mmol) in MeCN (4 mL) under nitrogen at rt was added iodoethane (121 µL, 1.50 mmol). The temperature was increased to 60 °C and the resulting mixture was stirred overnight. The reaction was cooled to rt and concentrated under reduced pressure. The resulting residue was diluted with water (30 mL) and extracted with EtOAc (3x 30 mL). The combined organic extracts were dried over $MgSO_4$, filtered and concentrated under reduced pressure. Purification by column chromatography eluting with hexane: EtOAc, 0 – 100 % afforded compound **2** (142 mg, 82 % yield) as an orange oil that crystallised upon standing.

Rf 0.20 (hexane: EtOAc, 3: 2); ^{1}H NMR (400 MHz, $CDCl_3$) δ 7.49 (d, *J* = 1.7 Hz, 1H), 6.40 (d, *J* = 1.7 Hz, 1H), 4.19 – 4.07 (m, 5H), 2.55 (s, 3H), 2.47 (s, 3H), 1.44 (t, *J* = 7.3 Hz, 3H); ^{13}C NMR (101 MHz, $CDCl_3$) δ 154.3, 141.9, 139.8, 138.9, 135.9, 92.1, 44.2, 35.9, 15.3, 14.6, 9.7; HRMS (CI) *m/z* calc. for $C_{11}H_{17}N_6$ $[M+H]^+$ 233.1499, found: 233.1509.

(*E*)-1-isopropyl-3,5-dimethyl-4-((1-methyl-1*H*-pyrazol-5-yl)diazenyl)-1*H*-pyrazole (3)

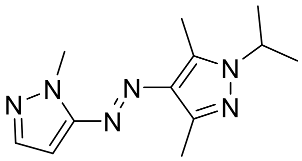

To a stirred solution of compound **7** (204 mg, 1.00 mmol) and K_2CO_3 (276 mg, 2.00 mmol) in MeCN (5 mL) under nitrogen at rt was added 2-iodopropane (200 µL, 2.00 mmol). The temperature was increased to 60 °C and the resulting mixture was stirred overnight. Then, more 2-iodopropane (200 µL, 2.00 mmol) was added, the temperature was increased to 80 °C and stirring continued for further 2 h. The reaction was cooled to rt and concentrated under reduced pressure. The resulting residue was diluted with water (40 mL) and extracted with EtOAc (3x 40 mL). The combined organic extracts were dried over $MgSO_4$, filtered and concentrated under reduced pressure. Purification by column chromatography eluting with hexane: EtOAc, 0 – 100 % afforded compound **3** (123 mg, 50 % yield) as an orange oil that crystallised upon standing.

Rf 0.55 (hexane: EtOAc, 3: 2); ^{1}H NMR (400 MHz, $CDCl_3$) δ 7.48 (d, *J* = 1.8 Hz, 1H), 6.38 (d, *J* = 1.8 Hz, 1H), 4.45 (hept, *J* = 6.7 Hz, 1H), 4.10 (s, 3H), 2.55 (s, 3H), 2.48 (s, 3H), 1.50 (d, *J* = 6.7 Hz, 6H); ^{13}C NMR (101 MHz, $CDCl_3$) δ 154.3, 141.4, 139.6, 138.9, 135.7, 91.9, 50.1, 35.8, 22.2, 14.9, 9.6; HRMS (CI) *m/z* calc. for $C_{12}H_{19}N_6$ $[M+H]^+$ 247.1666, found: 247.1655.

(*E*)-3,5-dimethyl-4-((1-methyl-1*H*-pyrazol-5-yl)diazenyl)-1-propyl-1*H*-pyrazole (4)

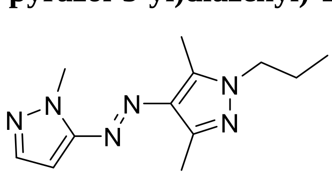

To a stirred solution of compound **7** (153 mg, 0.750 mmol) and K_2CO_3 (207 mg, 1.50 mmol) in MeCN (4 mL) under nitrogen at rt was added 1-iodopropane (146 µL, 1.50 mmol). The temperature was increased to 60 °C and the resulting mixture was stirred overnight. The reaction

was cooled to rt and concentrated under reduced pressure. The resulting residue was diluted with water (30 mL) and extracted with EtOAc (3x 30 mL). The combined organic extracts were dried over $MgSO_4$, filtered and concentrated under reduced pressure. Purification by column chromatography eluting with hexane: EtOAc, 0 – 100 % afforded compound **4** (186 mg, 90 % yield) as an orange oil.

Rf 0.24 (hexane: EtOAc, 3: 2); ^{1}H NMR (400 MHz, $CDCl_3$) δ 7.49 (d, J = 2.1 Hz, 1H), 6.39 (d, J = 2.1 Hz, 1H), 4.11 (s, 3H), 3.99 (t, J = 7.3 Hz, 2H), 2.55 (s, 3H), 2.47 (s, 3H), 1.87 (h, J = 7.3 Hz, 2H), 0.95 (t, J = 7.3 Hz, 3H); ^{13}C NMR (101 MHz, $CDCl_3$) δ 154.3, 141.8, 140.3, 138.9, 135.8, 92.1, 50.8, 35.9, 23.4, 14.7, 11.3, 9.9; HRMS (CI) *m/z* calc. for $C_{12}H_{19}N_6$ $[M+H]^+$ 247.1666, found: 247.1657.

3. NMR Analysis of *E* and *Z* isomers of Compounds 1-4

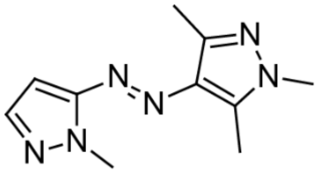

Compound 1 (*E*)

^{1}H NMR: (400 MHz, 298 K, MeCN-d_3) δ 7.46 (d, *J* = 2.1 Hz, 1H), 6.39 (d, *J* = 2.1 Hz, 1H), 4.08 (s, 3H), 3.74 (s, 3H), 2.53 (s, 3H), 2.42 (s, 3H).

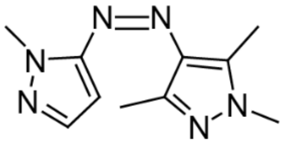

Compound 1 (*Z*)

^{1}H NMR: (400 MHz, 298 K, MeCN-d_3) δ 7.38 (d, *J* = 2.3 Hz, 1H), 5.49 (d, *J* = 2.3 Hz, 1H), 4.16 (s, 3H), 3.69 (s, 3H), 2.10 (s, 3H), 1.70 (s, 3H).

^1H NMR

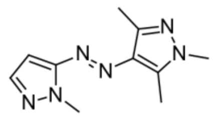

E-1

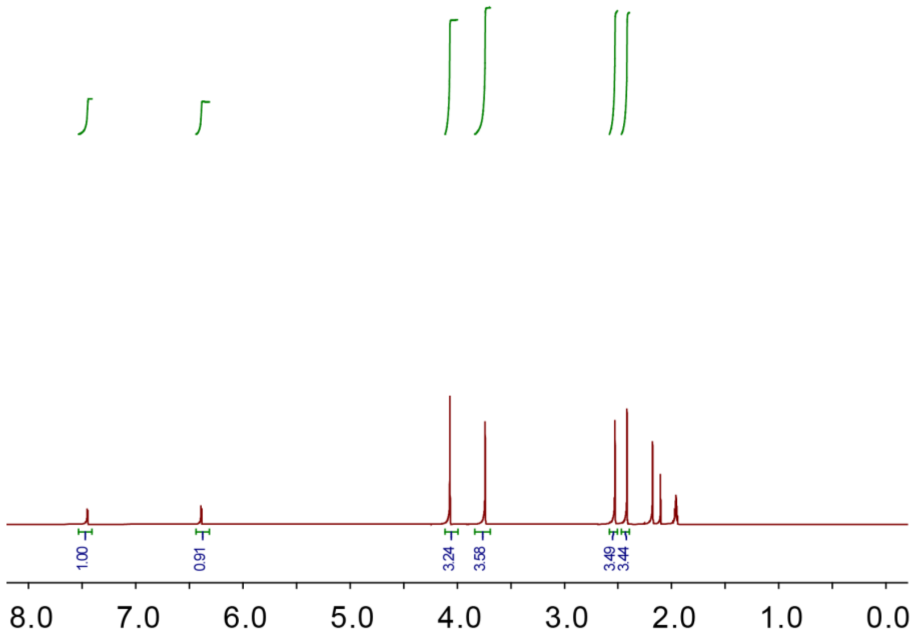

^1H NMR

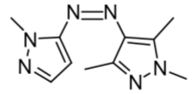

Z-1

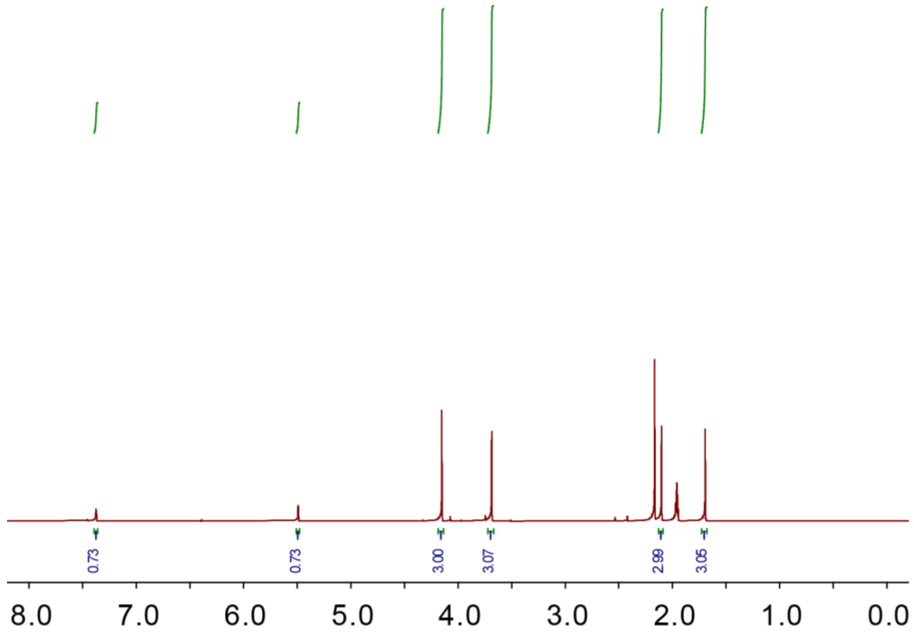

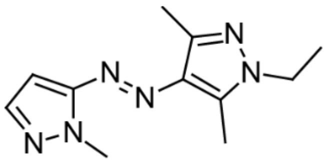

Compound 2 (*E*)

^{1}H NMR: (400 MHz, 298 K, MeCN-d_3) δ 7.45 (d, *J* = 2.1 Hz, 1H), 6.39 (d, *J* = 2.1 Hz, 1H), 4.08 (s, 3H), 4.11-4.06 (q, *J* = 7.3 Hz, 2H), 2.55 (s, 3H), 2.42 (s, 3H), 1.40-1.36 (t, *J* = 7.3 Hz, 3H).

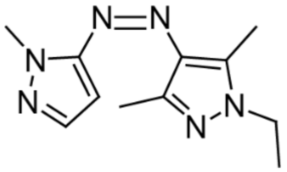

Compound 2 (*Z*)

^{1}H NMR: (400 MHz, 298 K, MeCN-d_3) δ 7.38 (d, *J* = 2.2 Hz, 1H), 5.45 (d, *J* = 2.2 Hz, 1H), 4.16 (s, 3H), 4.07-3.99 (q, *J* = 7.2 Hz, 2H), 2.11 (s, 3H), 1.70 (s, 3H), 1.36-1.33 (t, *J* = 7.2 Hz, 3H).

¹H NMR

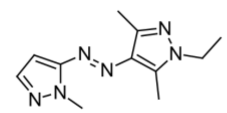

E-2

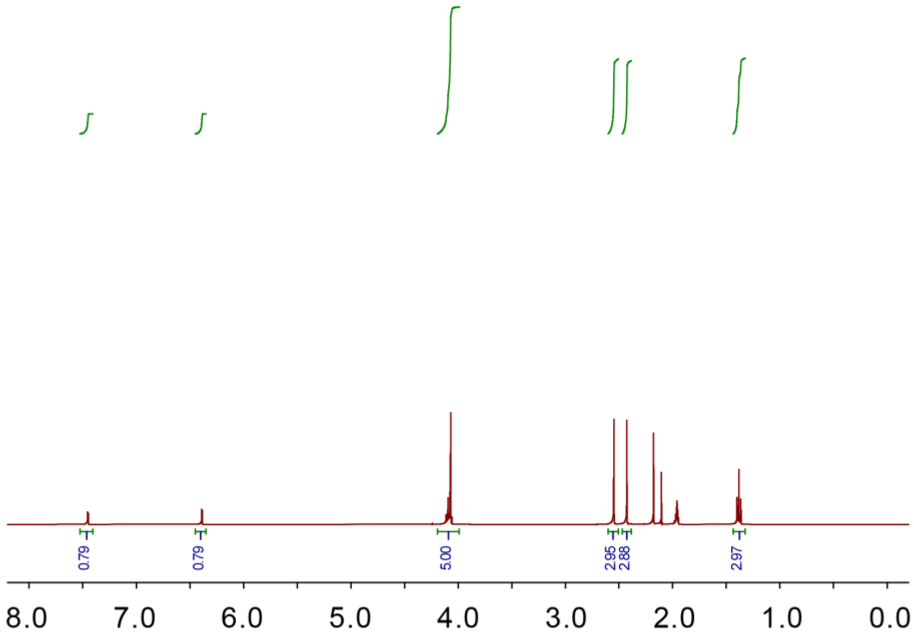

¹H NMR

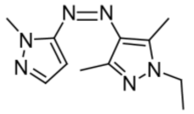

Z-2

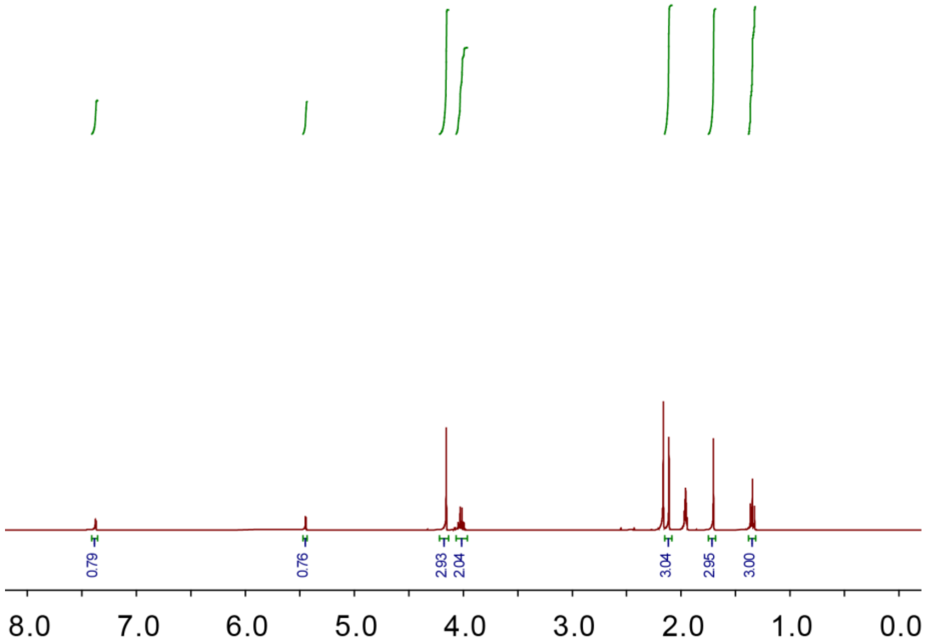

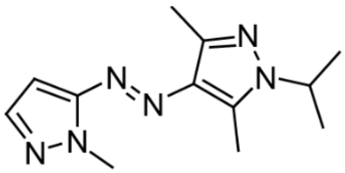

Compound 3 (*E*)

^{1}H NMR: (400 MHz, 298 K, MeCN-d_3) δ 7.45 (d, *J* = 2.1 Hz, 1H), 6.39 (d, *J* = 2.1 Hz, 1H), 4.61-4.51 (m, *J* = 6.6 Hz, 1H), 4.07 (s, 3H), 2.56 (s, 3H), 2.43 (s, 3H), 1.45-1.43 (d, *J* = 6.6 Hz, 6H).

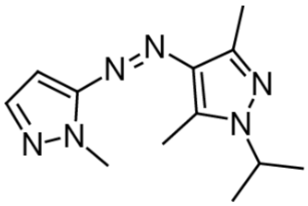

Compound 3 (*Z*)

^{1}H NMR: (400 MHz, 298 K, MeCN-d_3) δ 7.37 (d, *J* = 2.3 Hz, 1H), 5.42 (d, *J* = 2.3 Hz,1H), 4.49-4.42 (m, *J* = 6.6 Hz, 1H), 4.16 (s, 3H), 2.12 (s, 3H), 1.70 (s, 3H) 1.42-1.40 (d, *J* = 6.6 Hz, 6H).

¹H NMR

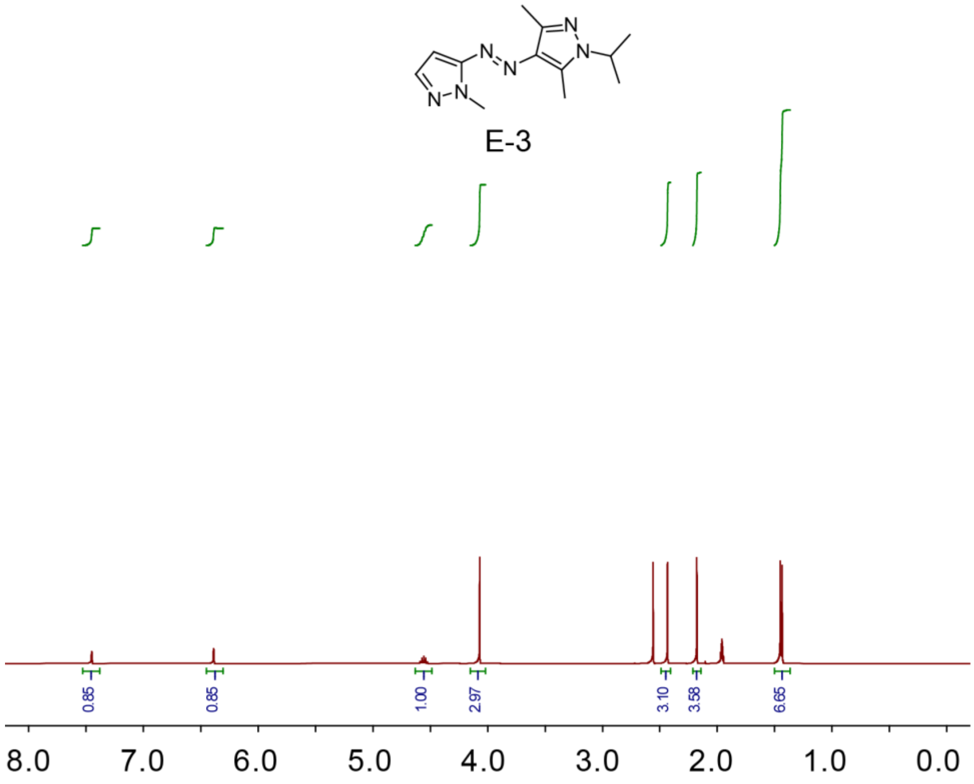

N
N
N
N
N
N
E-3
0.85
0.85
1.00
2.97
3.10
3.58
6.65
8.0
7.0
6.0
5.0
4.0
3.0
2.0
1.0
0.0

¹H NMR

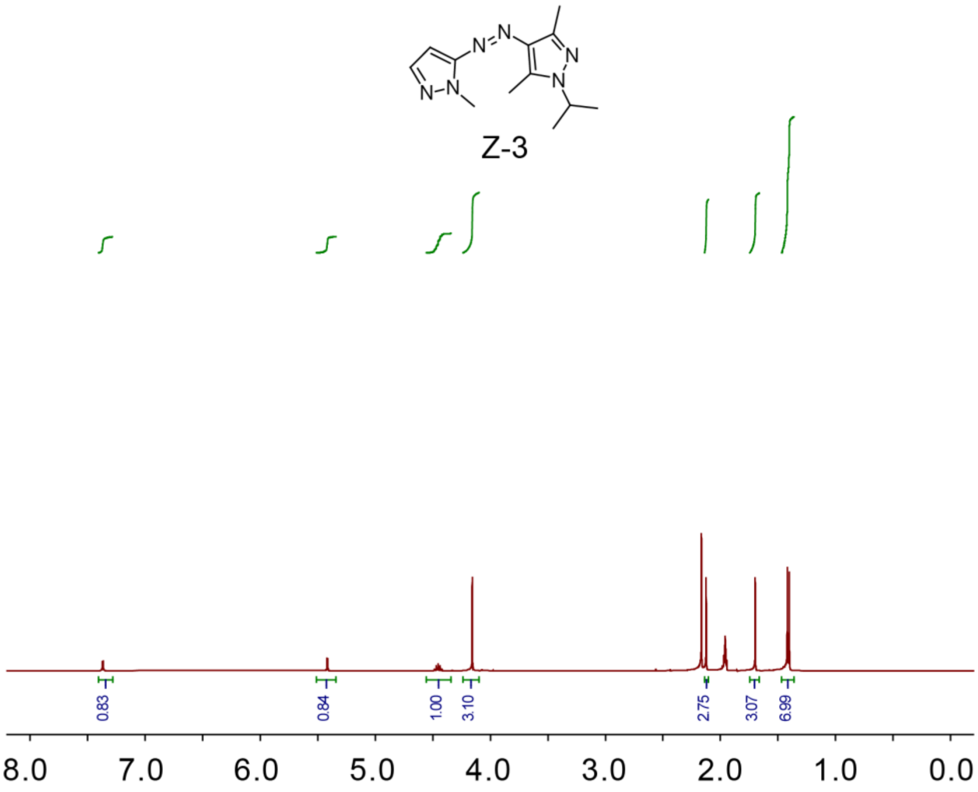

N
N
N
N
N
N
Z-3
0.83
0.84
1.00
3.10
2.75
3.07
6.99
8.0
7.0
6.0
5.0
4.0
3.0
2.0
1.0
0.0

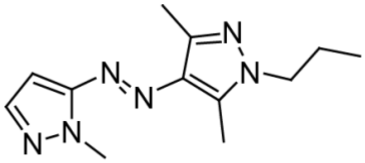

Compound 4 (*E*)

^{1}H NMR: (400 MHz, 298 K, MeCN-d_3) δ 7.46 (d, *J* = 2.1 Hz, 1H), 6.39 (d, *J* = 2.1 Hz, 1H), 4.07 (s, 3H), 4.02-3.99 (t, *J* = 7.1 Hz, 2H), 2.55 (s, 3H), 2.43 (s, 3H), 1.88-1.79 (sextet, *J* = 7.3 Hz, 2H), 0.95-0.91 (t, *J* = 7.4 Hz, 3H).

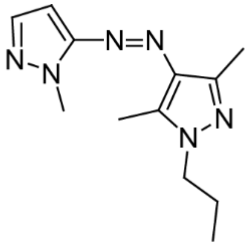

Compound 4 (*Z*)

^{1}H NMR: (400 MHz, 298 K, MeCN-d_3) δ 7.38 (d, *J* = 2.2 Hz, 1H), 5.44 (d, *J* = 2.2 Hz, 1H), 4.15 (s, 3H), 3.96-3.93 (t, *J* = 7.3 Hz, 2H), 2.11 (s, 3H), 1.84-1.76 (sextet, *J* = 7.3 Hz, 2H), 1.71 (s, 3H), 0.92-0.88 (t, *J* = 7.4 Hz, 3H).

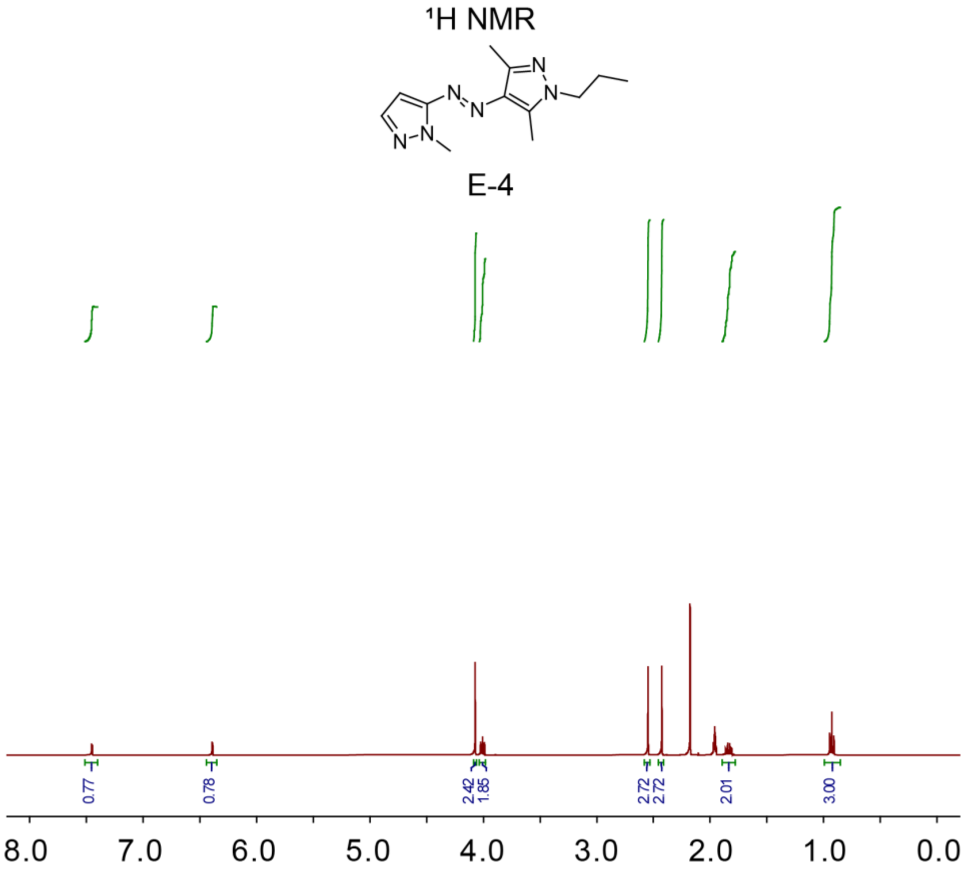
¹H NMR
E-4
0.77
0.78
2.42
1.85
2.72
2.72
2.01
3.00
8.0 7.0 6.0 5.0 4.0 3.0 2.0 1.0 0.0

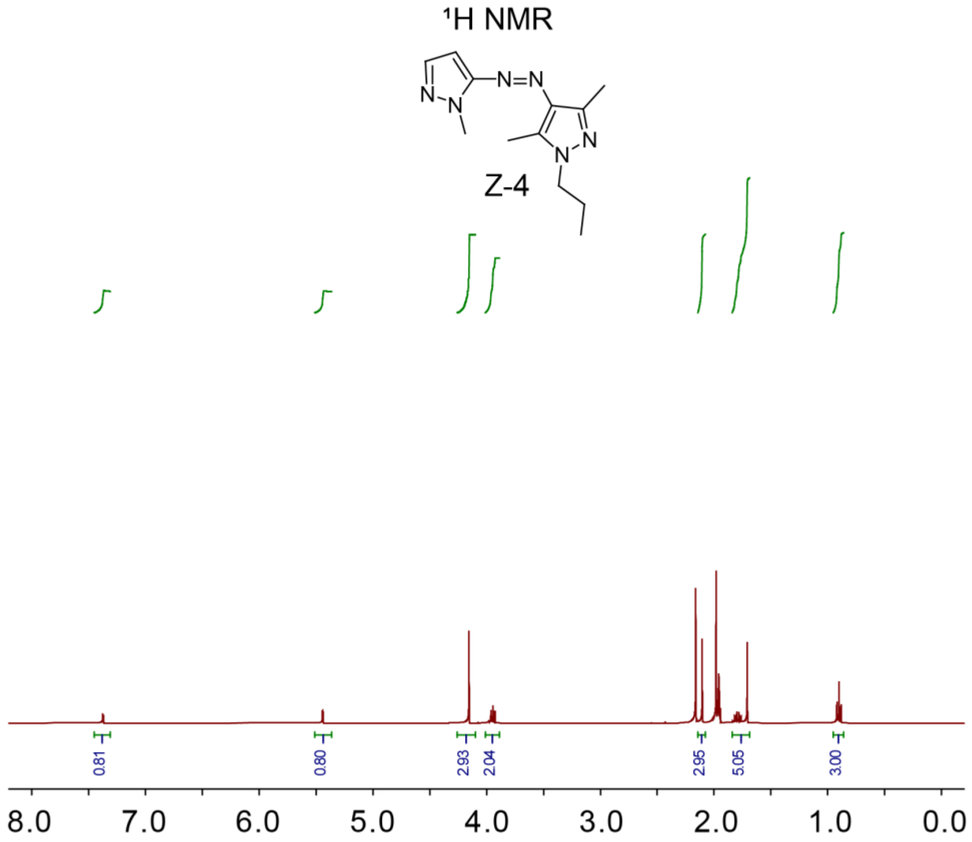

¹H NMR
Z-4
0.81
0.80
2.93
2.04
2.95
5.05
3.00
8.0
7.0
6.0
5.0
4.0
3.0
2.0
1.0
0.0

4. UV-Vis Absorption Spectra

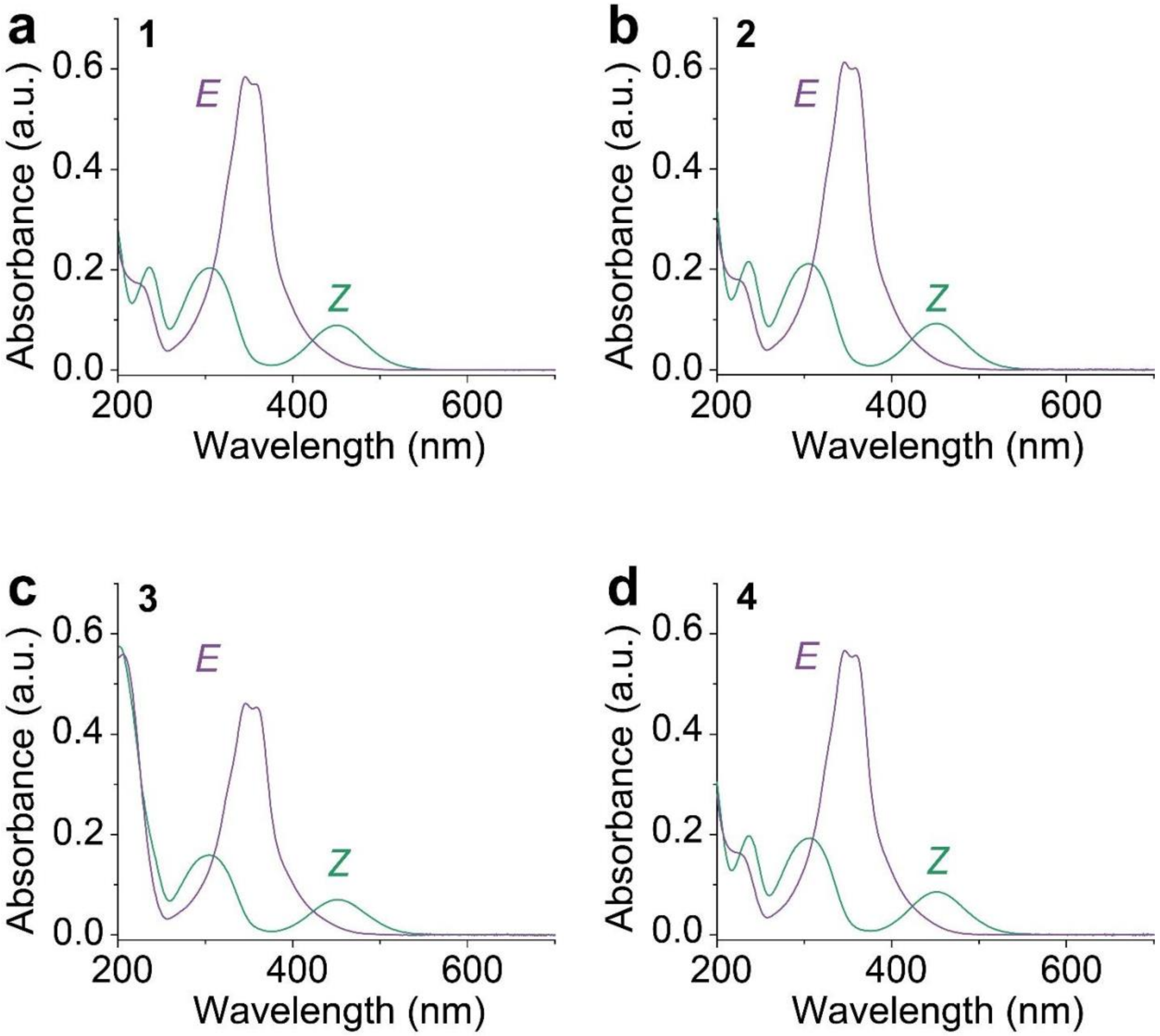

Fig. S7. UV-Vis absorption spectra of compounds **1-4**. The PSS of the *E* isomer was reached with 525 nm irradiation, and the PSS of the *Z* isomer was reached with 365 nm irradiation. Solutions were prepared to 25 µM in MeCN.

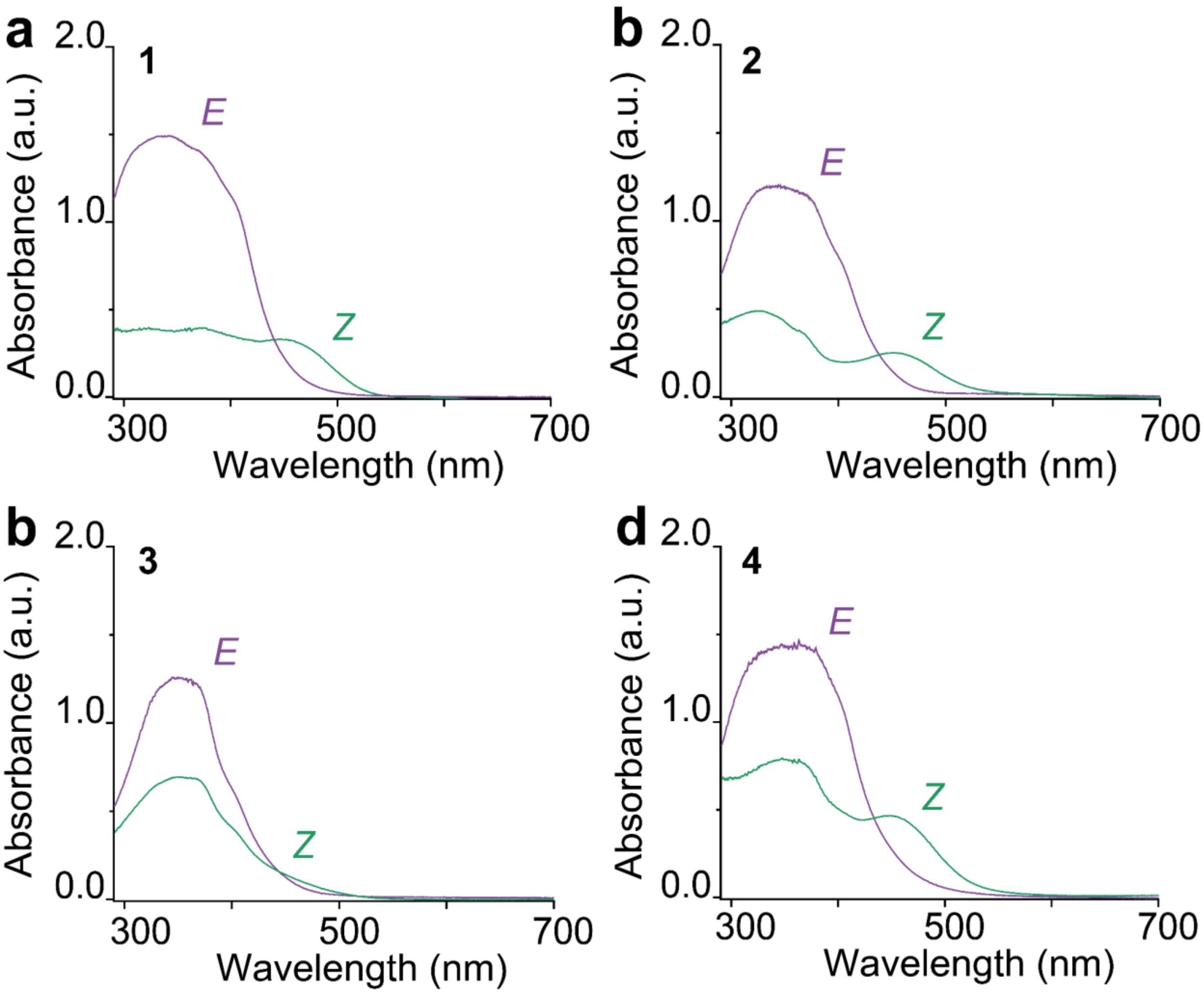

Fig. S8. UV-Vis absorption spectra of compounds **1-4** acquired in condensed phases. All compounds were irradiated for 72 hours to obtain the highest PSS possible. Unlike the UV Quartz cuvette that can absorb wavelengths as low as 200 nm, the glass slides used for condensed phase UV-Vis spectra can only absorb from 290 nm.

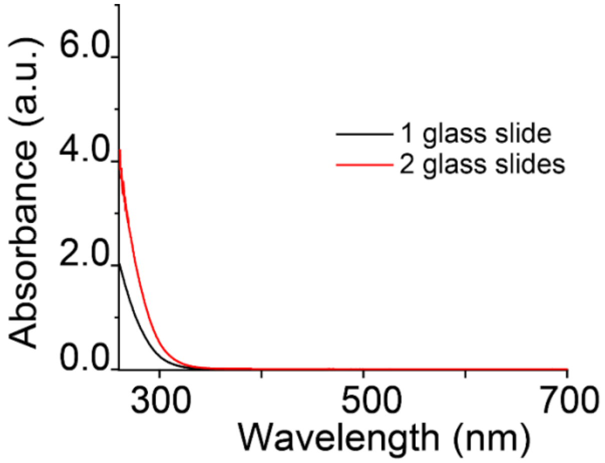

Figure S9. UV-Vis absorption spectra of blank glass slides used as sandwiched films for the condensed-phase thin film spectra.

5. Measuring Quantum Yields

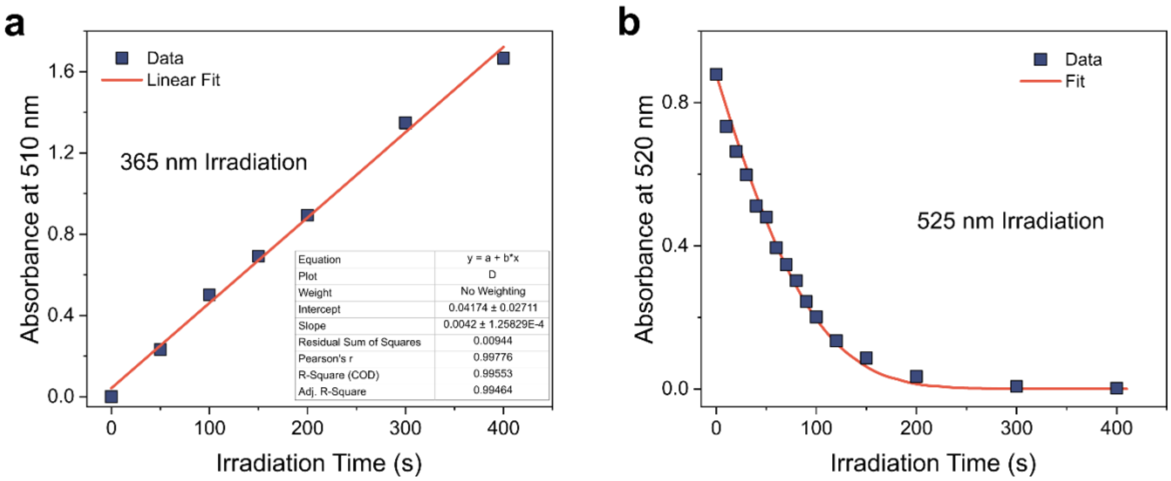

Fig. S10. a, Plot of the absorbance at 510 nm against irradiation time (365 nm NCSU276A LED) of an Fe(II) phenanthroline complex. The photon flux was calculated to be 1.845×10^{16} photons/s. **b**, Plot of the absorbance at 520 nm against irradiation time (525 nm NCSG219B-V1 LED) of the

ring-opening of Aberchrome 670. The photon flux was calculated to be 1.248×10^{16} photons/s using the fitting program in reference 1.

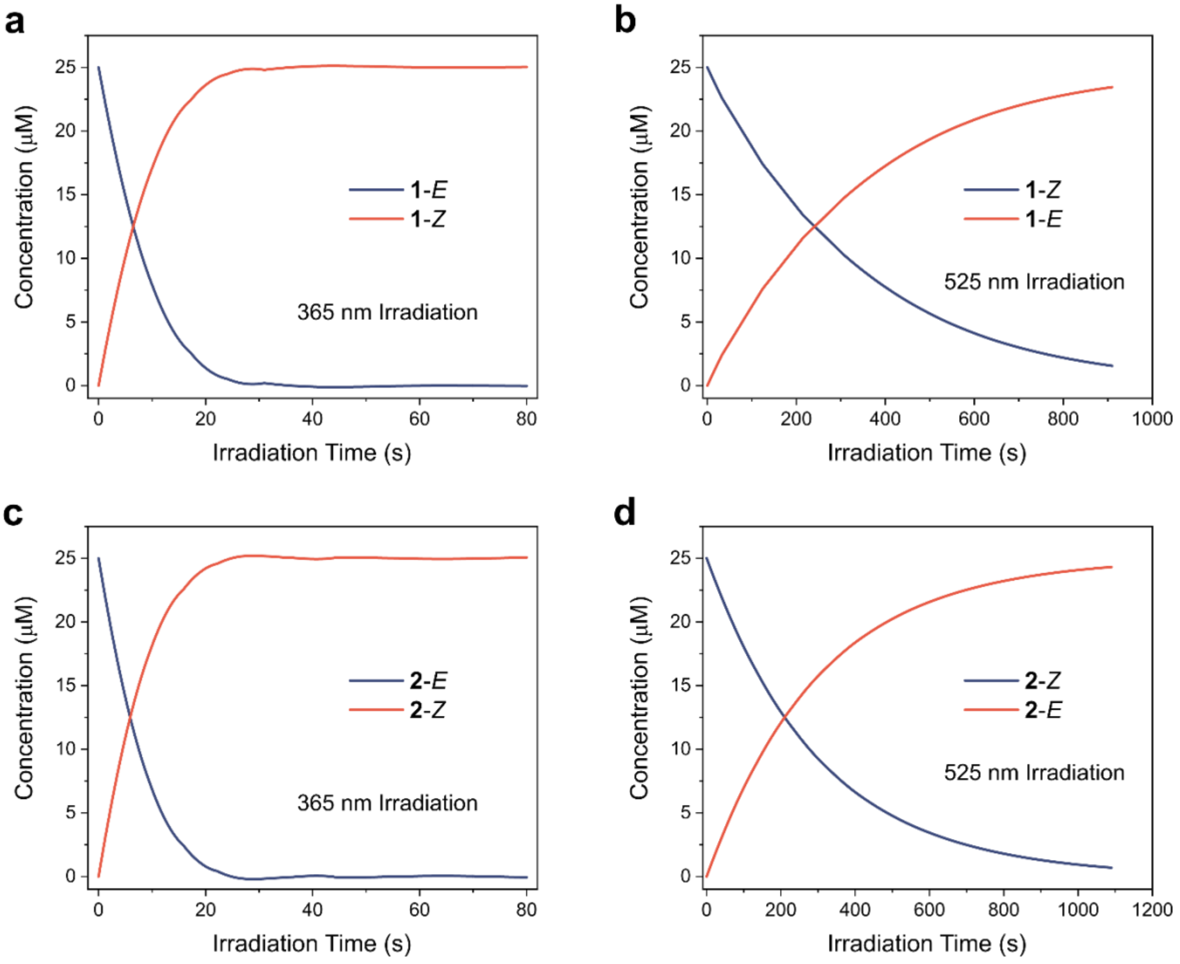

Fig. S11. **a**, Plot of concentration of switch **1** as a function of irradiation time with 365 nm light. **b**, Plot of concentration of switch **1** as a function of irradiation time with 525 nm light. **c**, Plot of concentration of switch **2** as a function of irradiation time with 365 nm light. **d**, Plot of concentration of switch **2** as a function of irradiation time with 525 nm light. The calculated quantum yields of photoswitching are presented in Table S1.

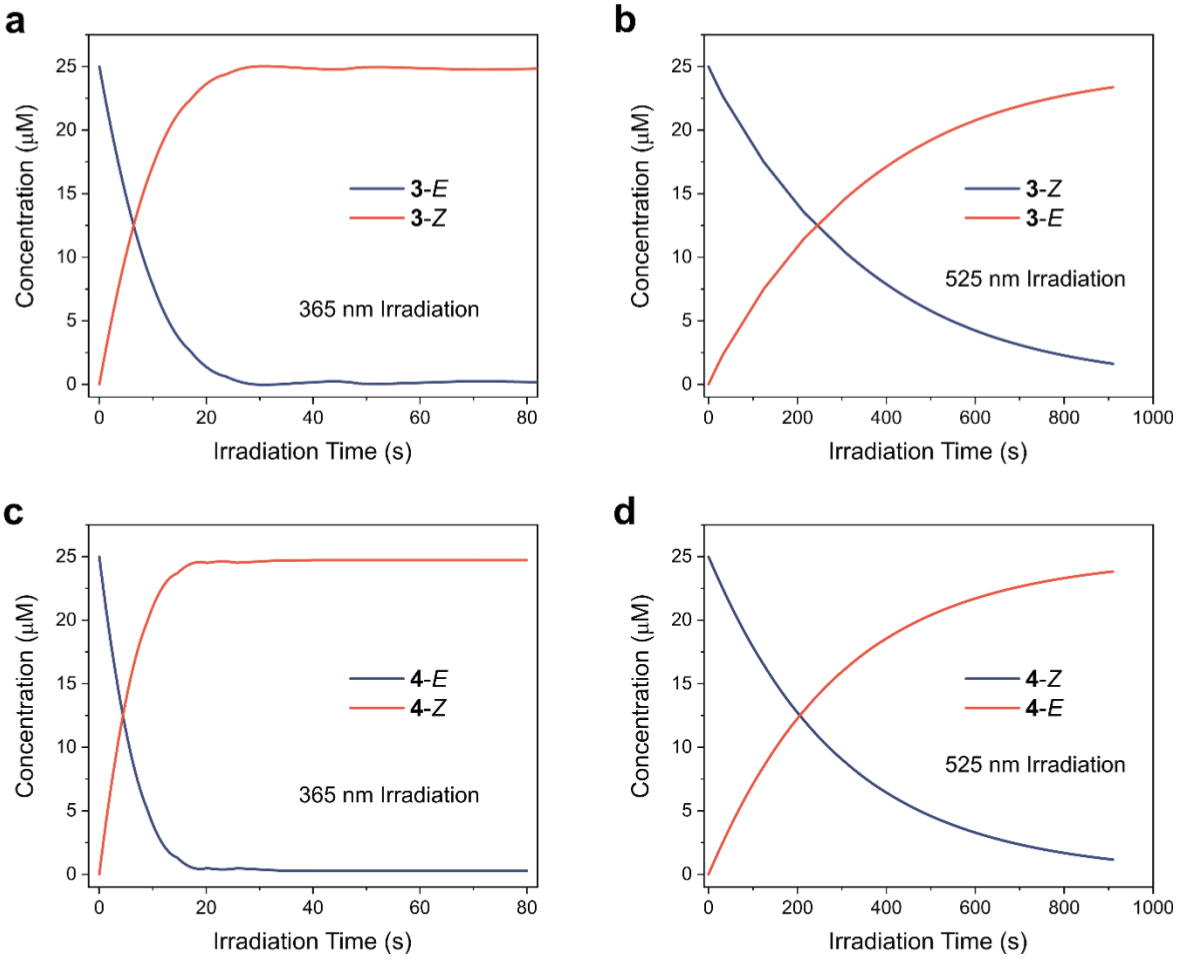

Fig. S12. **a**, Plot of concentration of switch **3** as a function of irradiation time with 365 nm light. **b**, Plot of concentration of switch **3** as a function of irradiation time with 525 nm light. **c**, Plot of concentration of switch **4** as a function of irradiation time with 365 nm light. **d**, Plot of concentration of switch **4** as a function of irradiation time with 525 nm light. The calculated quantum yields of photoswitching are presented in Table S1.

Table S1. Molar extinction coefficients and quantum yields of switches **1-4** at 365 nm and 525 nm irradiation.

	365 nm				525 nm			
Switch	ε_E (M^{-1} cm^{-1})	ε_Z (M^{-1} cm^{-1})	Φ_{EZ}	Φ_{ZE}	ε_E (M^{-1} cm^{-1})	ε_Z (M^{-1} cm^{-1})	Φ_{EZ}	Φ_{ZE}
**1**	21,273	457	0.33	0.04	9	331	0.03	0.60
**2**	22,151	640	0.35	0.04	7	329	0.04	0.63
**3**	21,897	467	0.33	0.03	10	335	0.02	0.58
**4**	22,000	451	0.34	0.01	20	363	0.02	0.58

6. Thermal Isomerization Kinetics and Fatigue Resistance

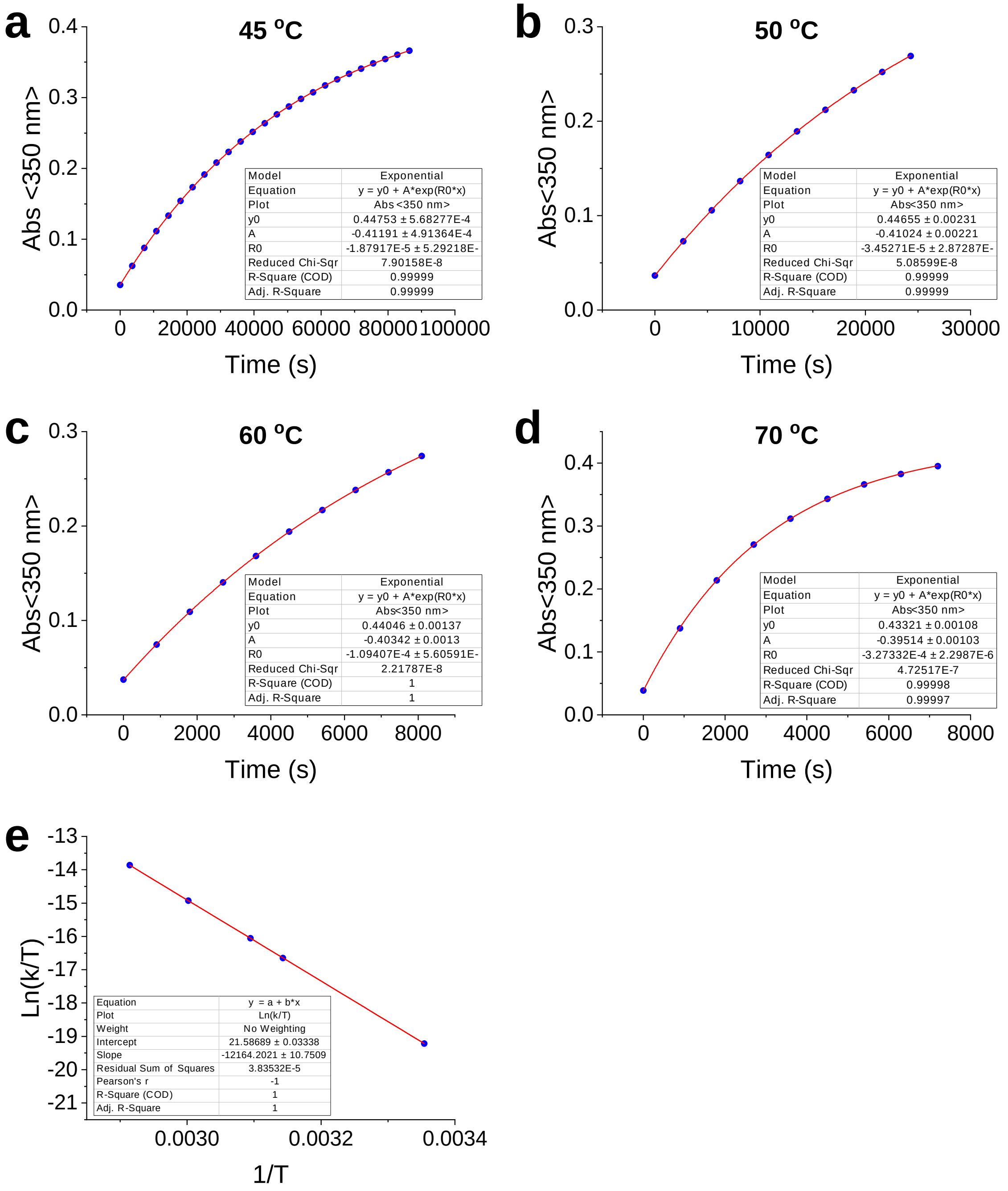

Fig. S13. Thermal isomerization kinetics of **1** at a) 45 °C, b) 50 °C, c) 60 °C and d) 70 °C and e) Eyring plot. The absorbance at 350 nm at each temperature was fitted to an exponential fit. The fitted parameters were used to obtain a Eyring plot that allowed determination of the thermal half-life at 25 °C to 6.0 days by extrapolation.

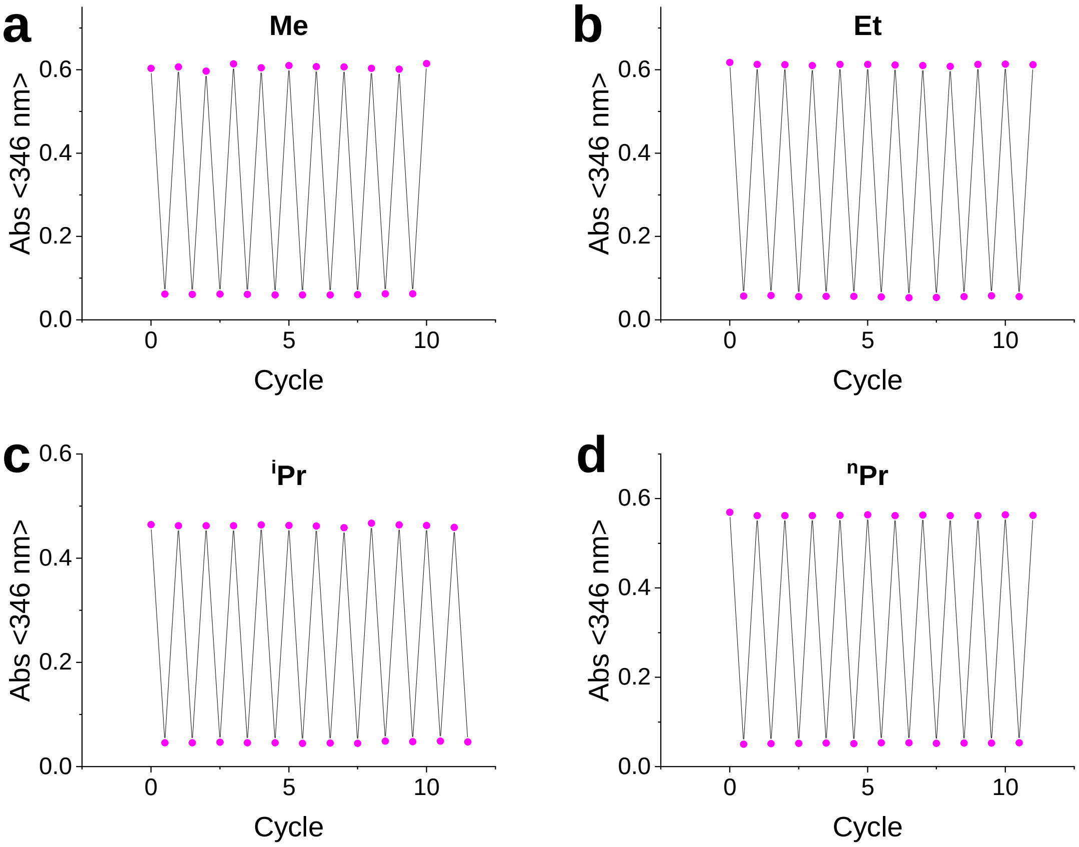

Fig. S14. Fatigue resistances of a) **1**, b) **2**, c) **3** and d) **4**. The absorbance at 346 nm was monitored over at least 10 switching cycles. The forward *E*-*Z* and the back *Z*-*E* photoisomerisations were achieved with 365 nm and 525 nm irradiation respectively. No sign of fatigue was evident throughout.

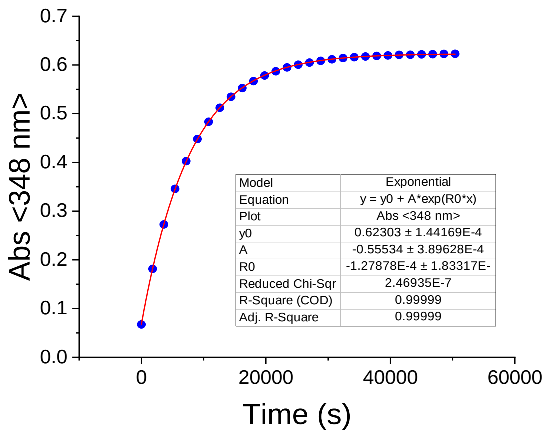

Fig. S15. Thermal isomerization kinetics of **4pzMe-5pzH-H** at 25 °C. The absorbance at 348 nm at each temperature was fitted to an exponential fit to allow determination of the thermal half-life to 1.5 h.

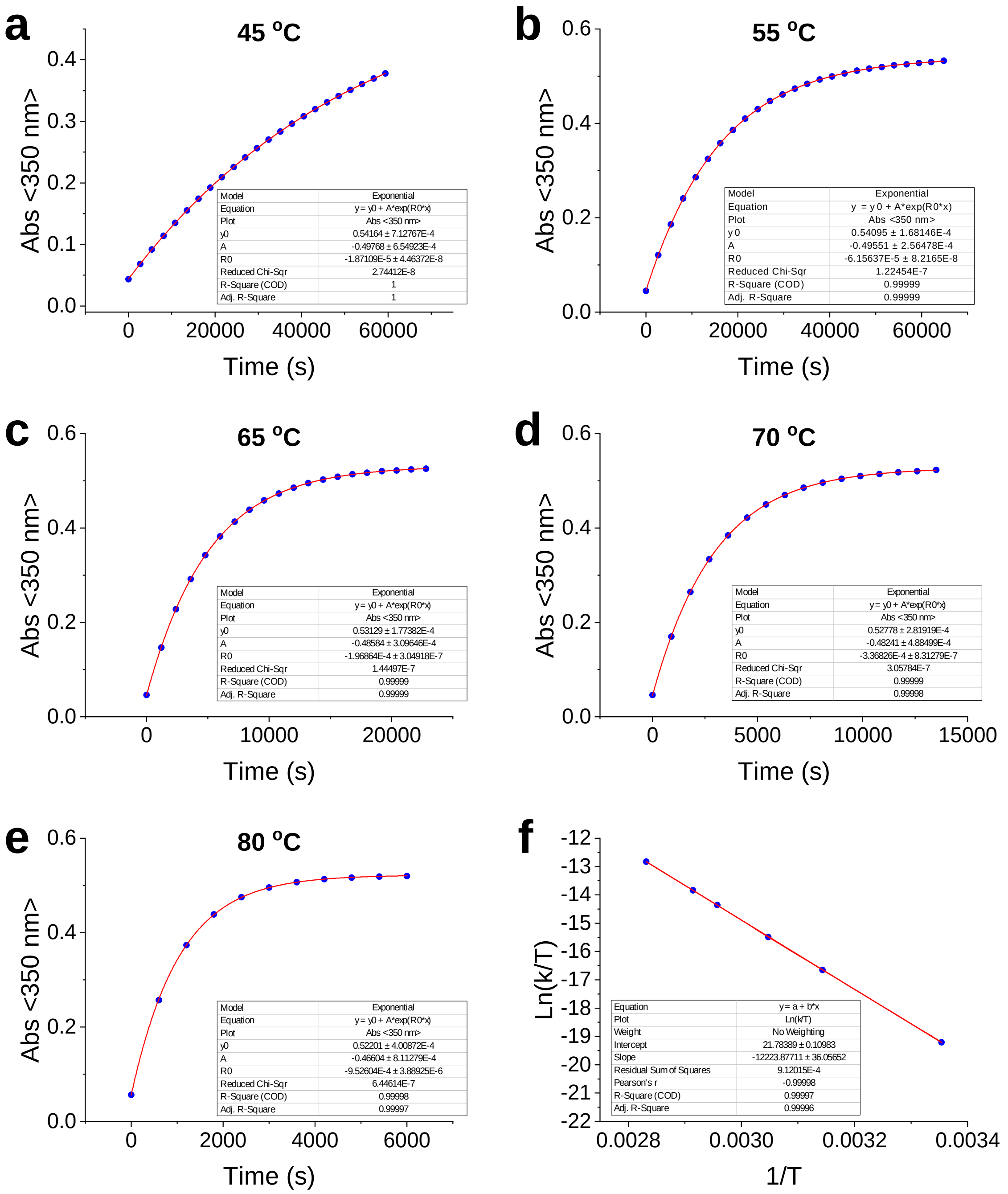

Fig. S16. Thermal isomerization kinetics of **2** at a) 45 °C, b) 55 °C, c) 65 °C, d) 70 °C and e) 80 °C and f) Eyring plot. The absorbance at 350 nm at each temperature was fitted to an exponential fit. The fitted parameters were used to obtain a Eyring plot that allowed determination of the thermal half-life at 25 °C to 5.9 days by extrapolation.

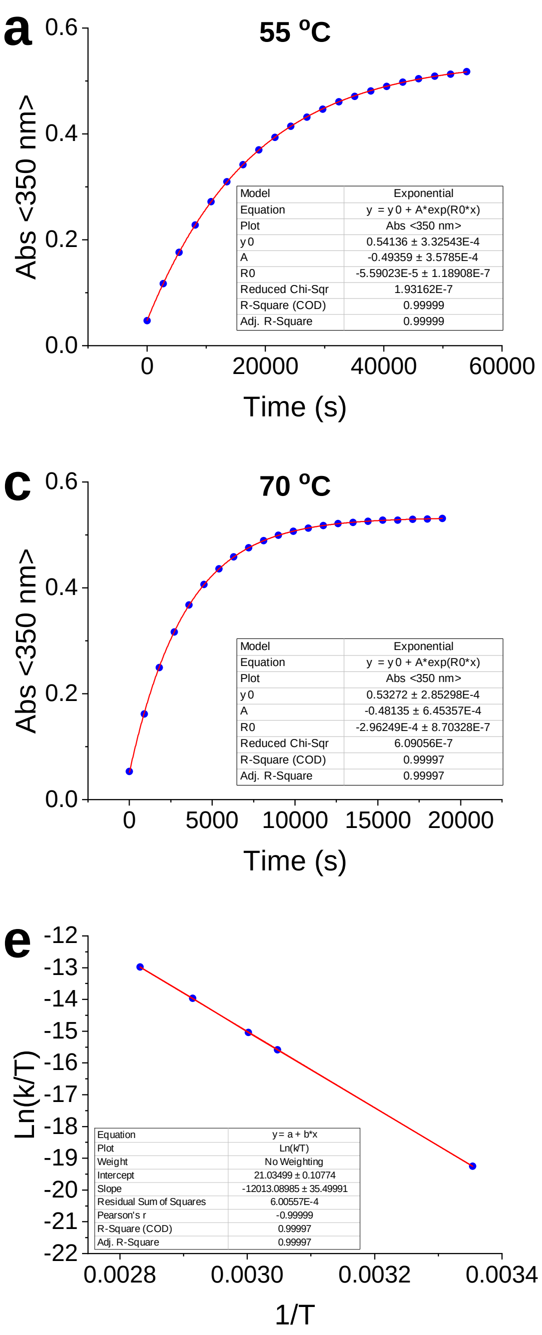

Fig. S17. Thermal isomerization kinetics of **3** at a) 55 °C, b) 60 °C, c) 70 °C and d) 80 °C and e) Eyring plot. The absorbance at 350 nm at each temperature was fitted to an exponential fit. The fitted parameters were used to obtain a Eyring plot that allowed determination of the thermal half-life at 25 °C to 6.2 days by extrapolation.

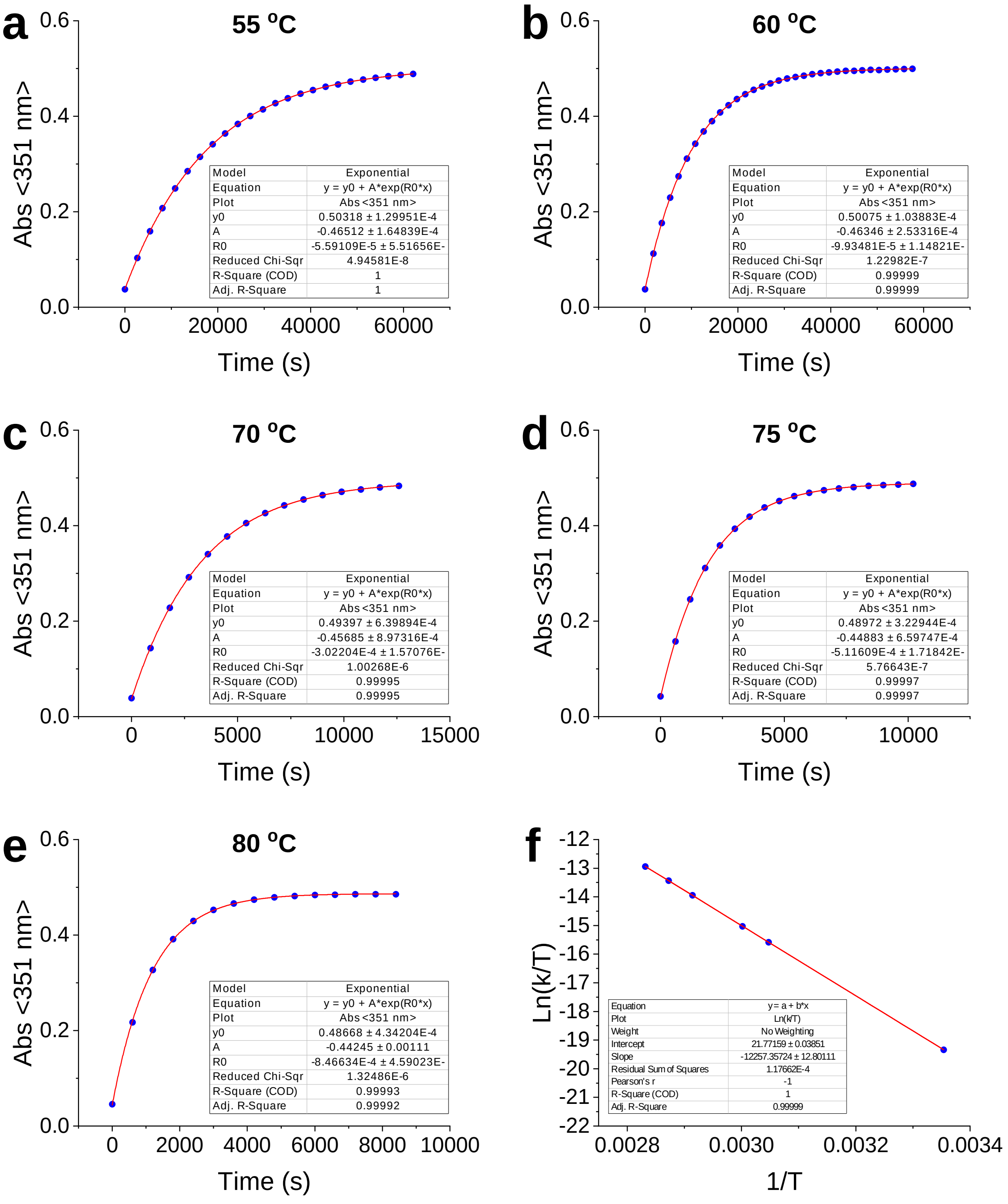

Fig. S18. Thermal isomerization kinetics of **4** at a) 55 °C, b) 60 °C, c) 70 °C, d) 75 °C and e) 80 °C and f) Eyring plot. The absorbance at 351 nm at each temperature was fitted to an exponential fit. The fitted parameters were used to obtain a Eyring plot that allowed determination of the thermal half-life at 25 °C to 6.7 days by extrapolation.

7. Differential Scanning Calorimetry Plots of Compounds 2 and 3

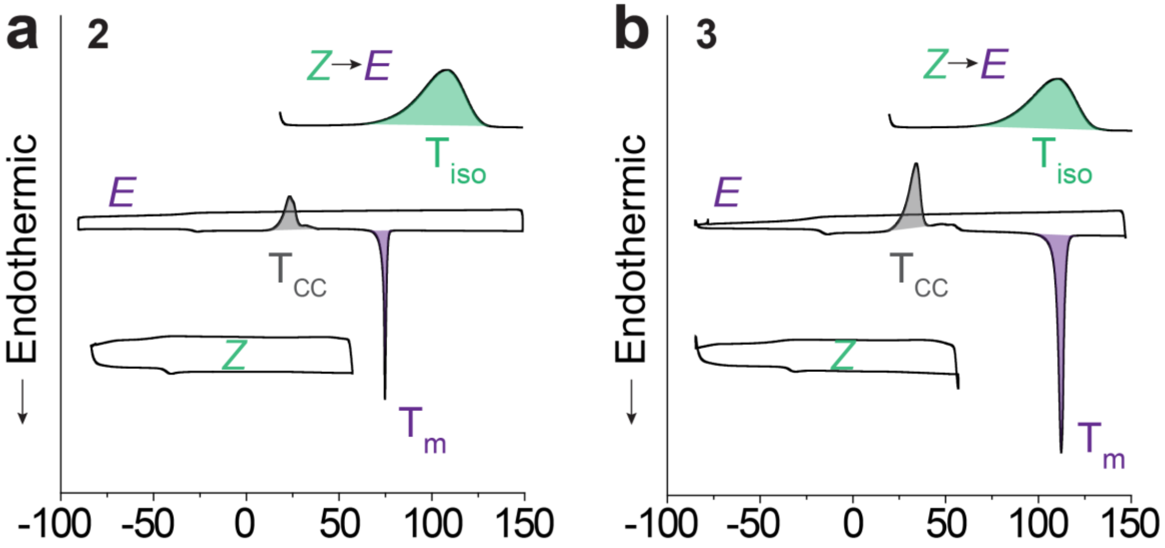

Fig. S19. DSC plots illustrating thermal properties of *Z*-*E* photoisomerization (top curve), *E* (middle curve), and *Z* (bottom curve) isomers upon heating and cooling for a) **2** and b) **3**. The following thermal parameters are labeled in plots: isomerization temperature (T_{iso}), cold-crystallization temperature (T_{cc}), and melting temperature (T_m). Scan rate is 10 ˚C/min. The first heating curve is shown in purple, and first cooling shown in grey.

8. Solar Irradiance Measurement

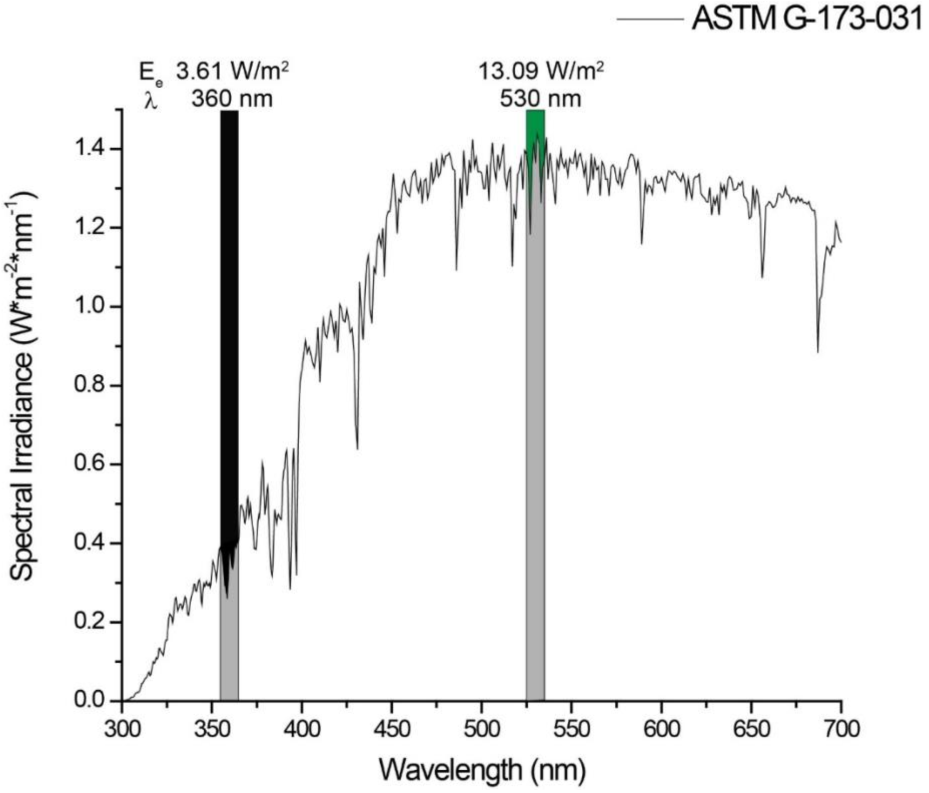

Fig. S20. Integration E_e of Air Mass 1.5 solar irradiance spectrum (ASTM G-173-031 reference spectra)[6] at 360 nm, and 530 nm with 10 nm bandwidth. The total integrated irradiance is 900.1 $W \cdot m^{-2}$.

9. Photoinduced Reversible Phase Transition of Compounds 1-4

Compound 1

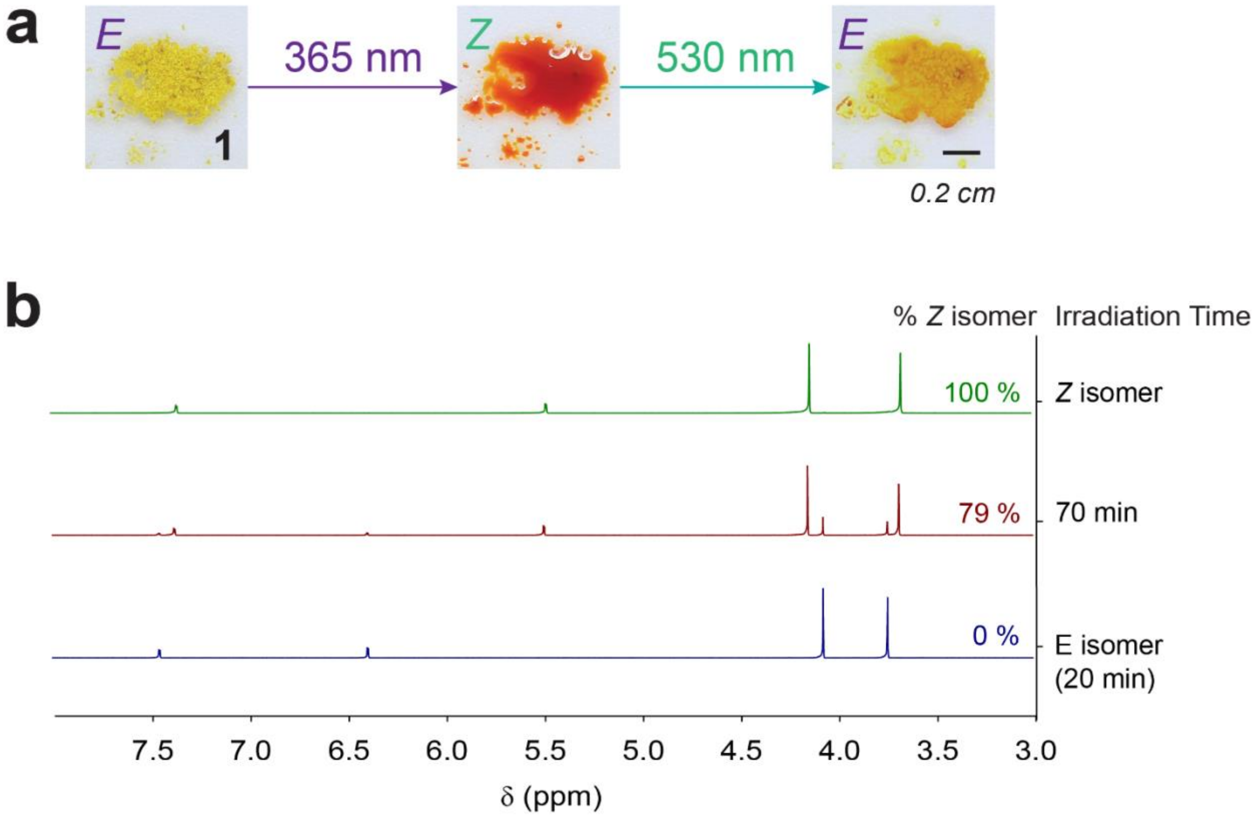

Fig. S21. a) Images demonstrating light-induced isomerization of compound **1**. The first image shows an *E*-rich compound, and the following images illustrate the change of the sample with 365 nm and 530 nm exposure. b) The *Z* ratios of corresponding films were determined by ^{1}H NMR.

Compound 2

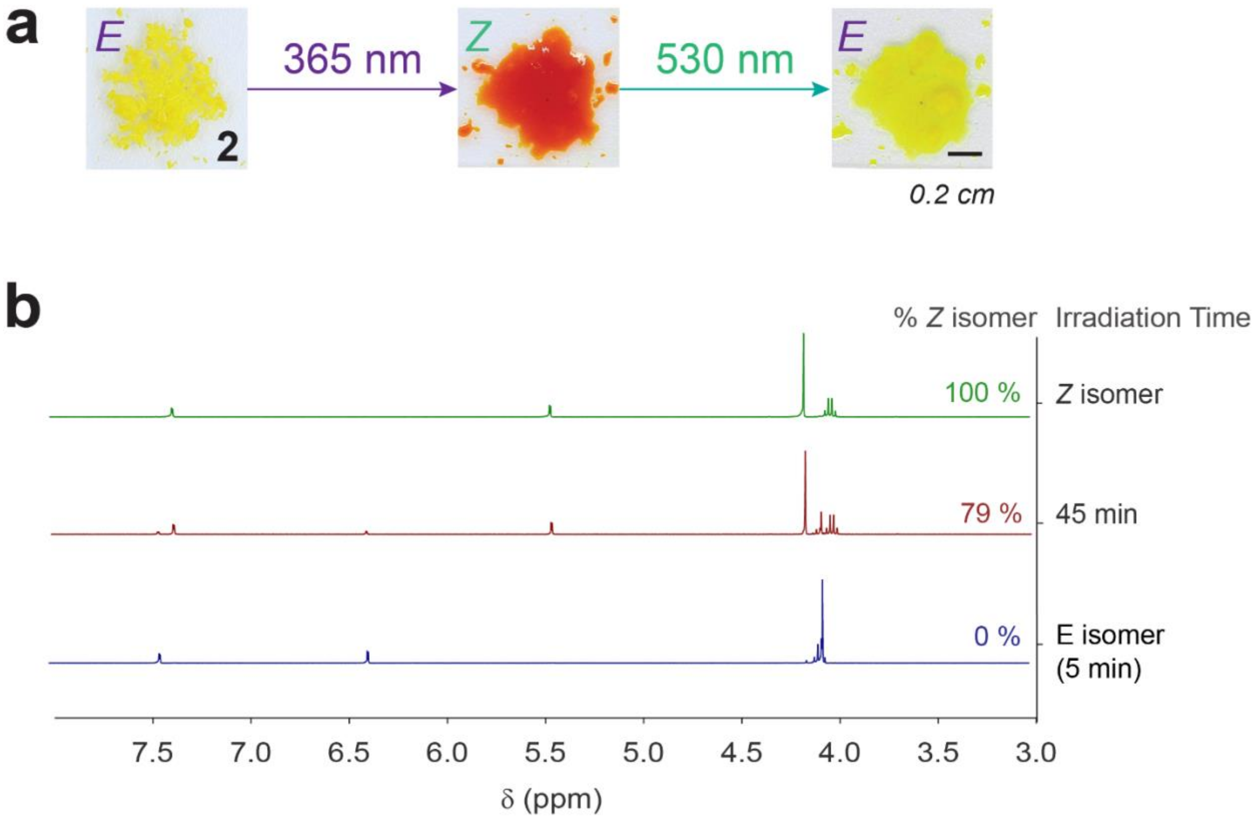

Fig. S22. a) Images demonstrating light-induced isomerization of compound **2**. The first image shows an *E*-rich compound, and the following images illustrate the change of the sample with 365 nm and 530 nm exposure. b) The *Z* ratios of corresponding films were determined by ^{1}H NMR.

Compound 3

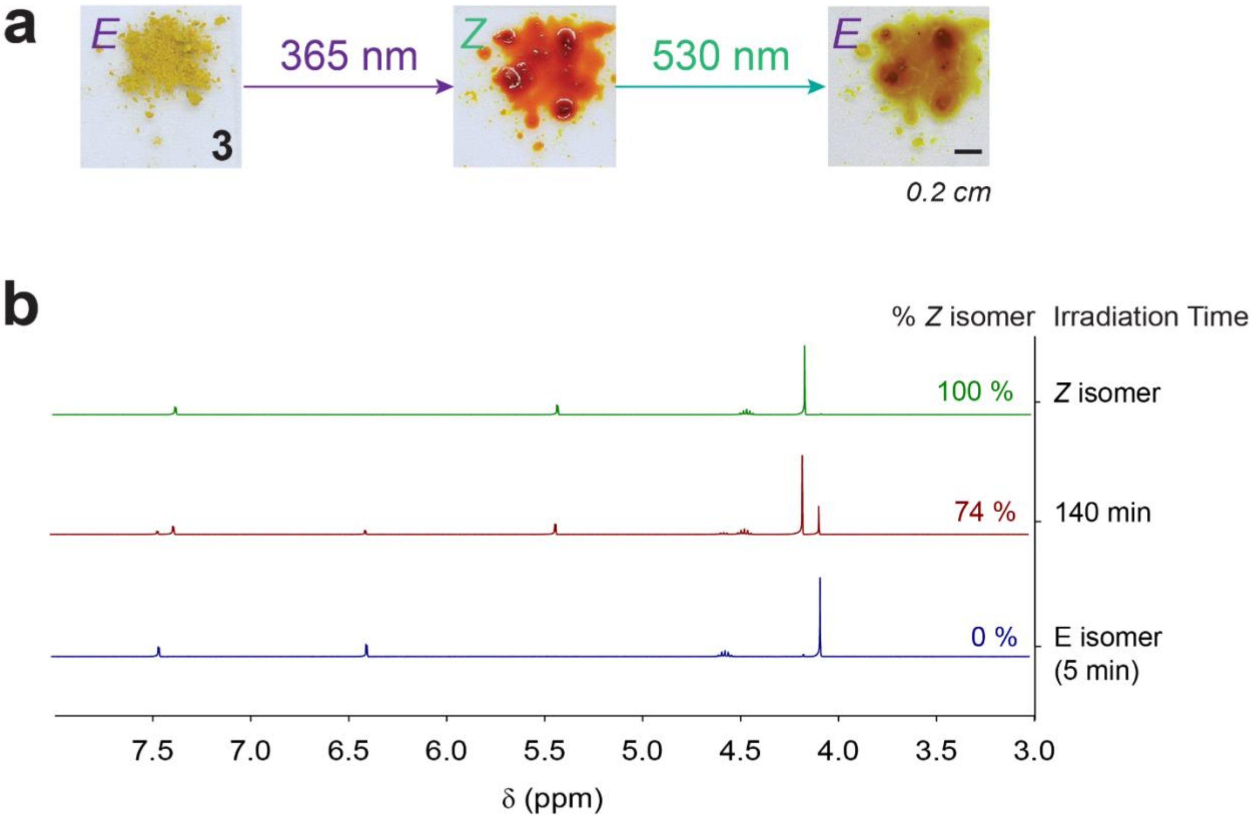

Fig. S23. a) Images demonstrating light-induced isomerization of compound **3**. The first image shows an *E*-rich compound, and the following images illustrate the change of the sample with 365 nm and 530 nm exposure. b) The *Z* ratios of corresponding films were determined by ^{1}H NMR.

Compound 4

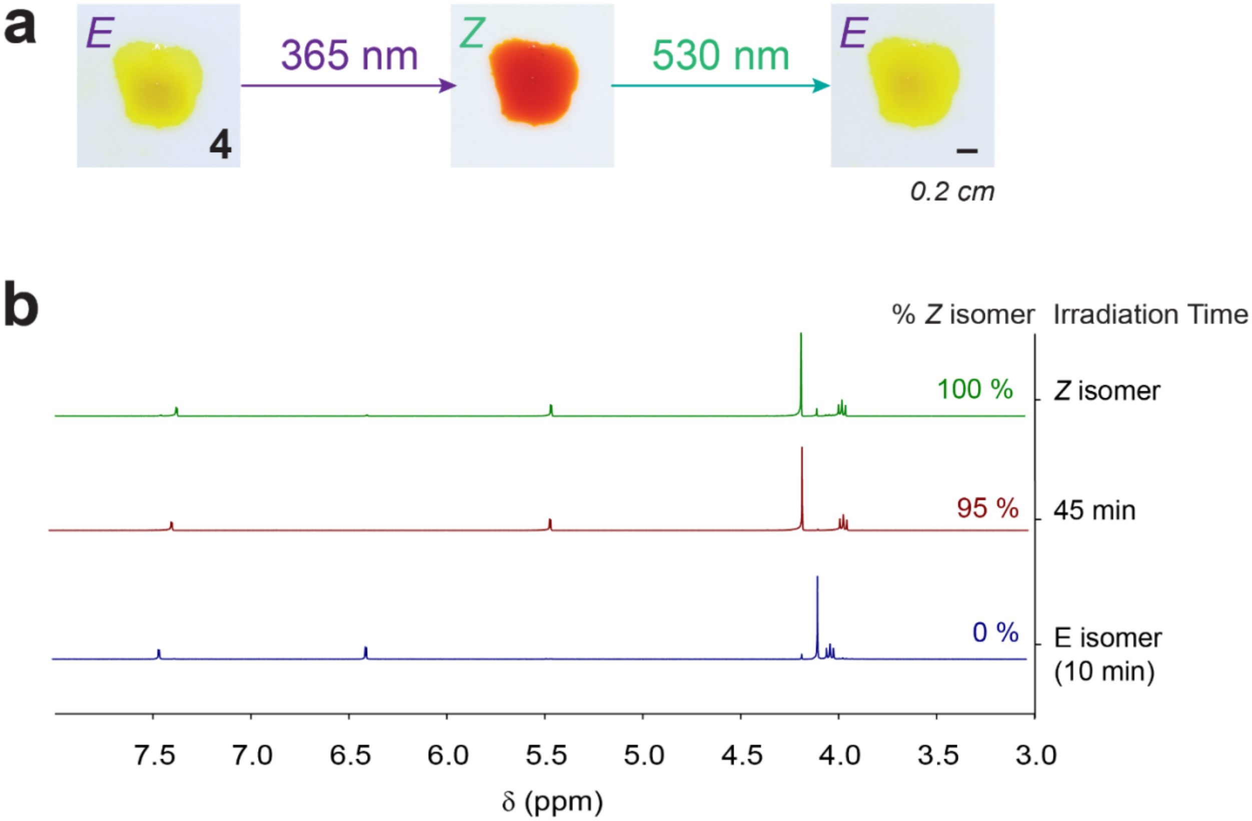

Fig. S24. Images demonstrating light-induced isomerization of compound **4**. The first image shows an *E*-rich compound, and the following images illustrate the change of the sample with 365 nm and 530 nm exposure. b) The *Z* ratios of corresponding films were determined by ^{1}H NMR.

10. X-Ray Diffraction Measurements

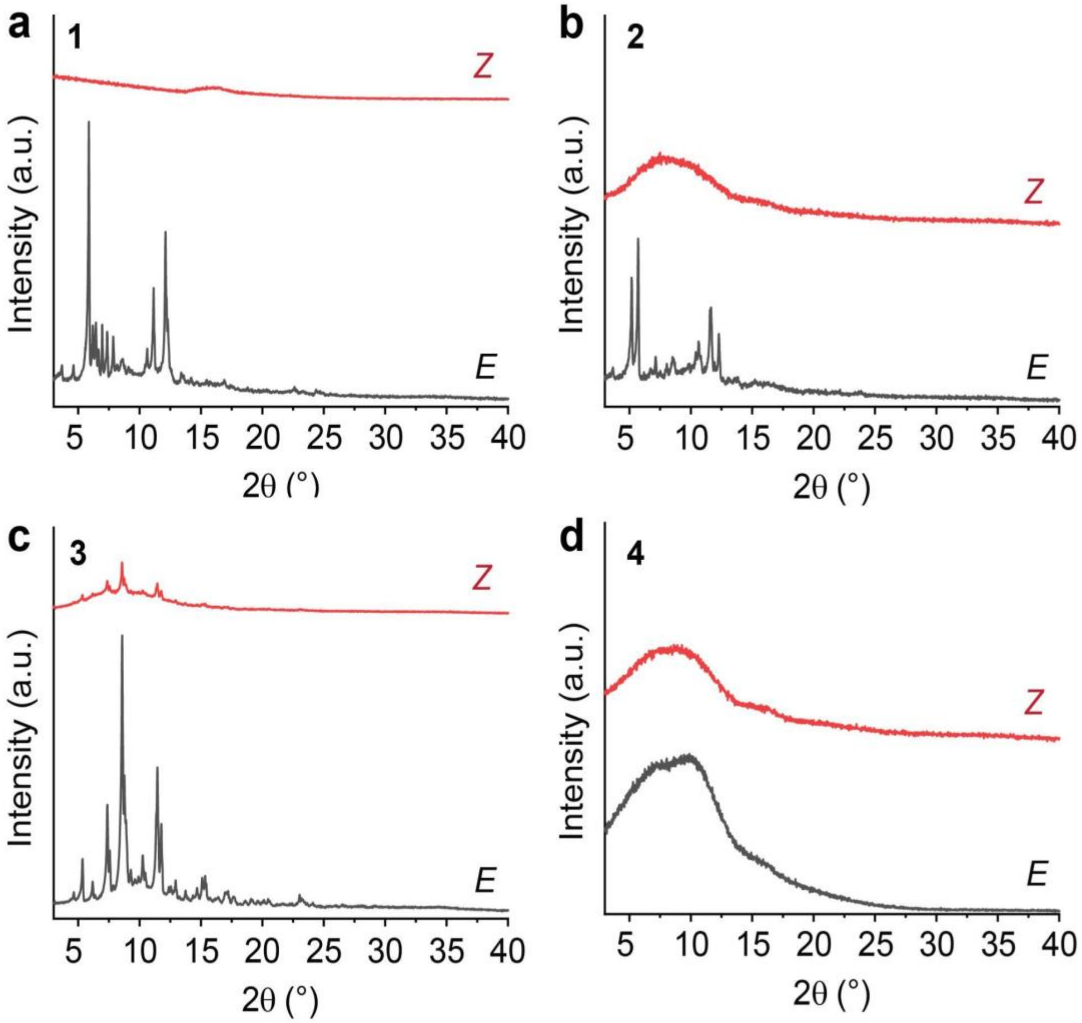

Fig. S25. X-ray diffraction measurement of *E* and *Z* isomers of compounds **1-4** (a-d).

11. Penetration Depth Measurements

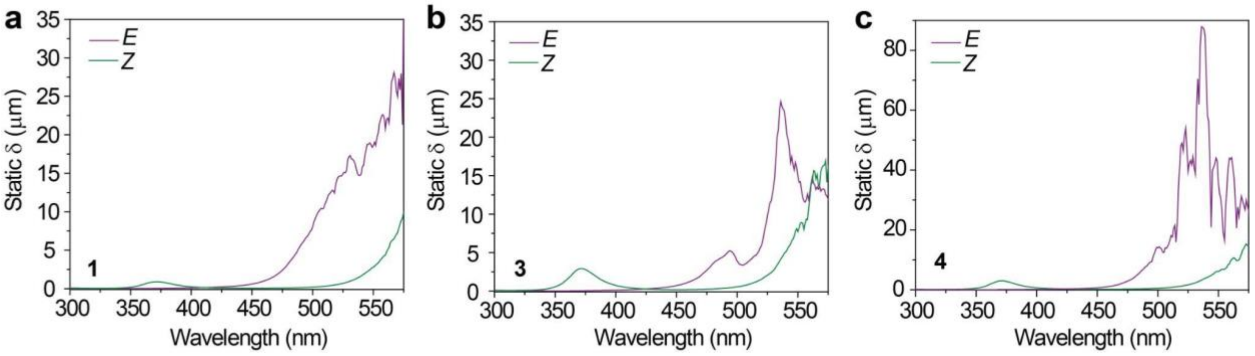

Fig. S26. Plots of the static penetration depth of compounds **1, 3,** and **4** for their *E* and *Z* isomeric forms. The static penetration depth was calculated using the procedure reported by Grossman and co-workers.[7] The UV-vis absorption data used in the calculation was smoothed using the Savitzky-Golay method (points window 10, polynomial order 2).

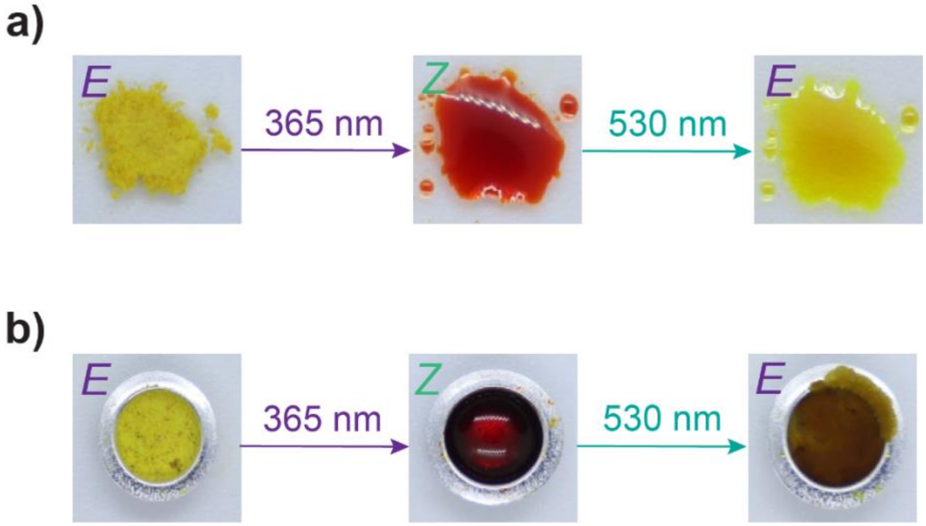

Fig. S27. Images illustrating the penetration depth studies of compound **2** with a) thickness *smaller than 450 μm* using thin films and b) thickness *larger than 450 μm* using aluminum pans.

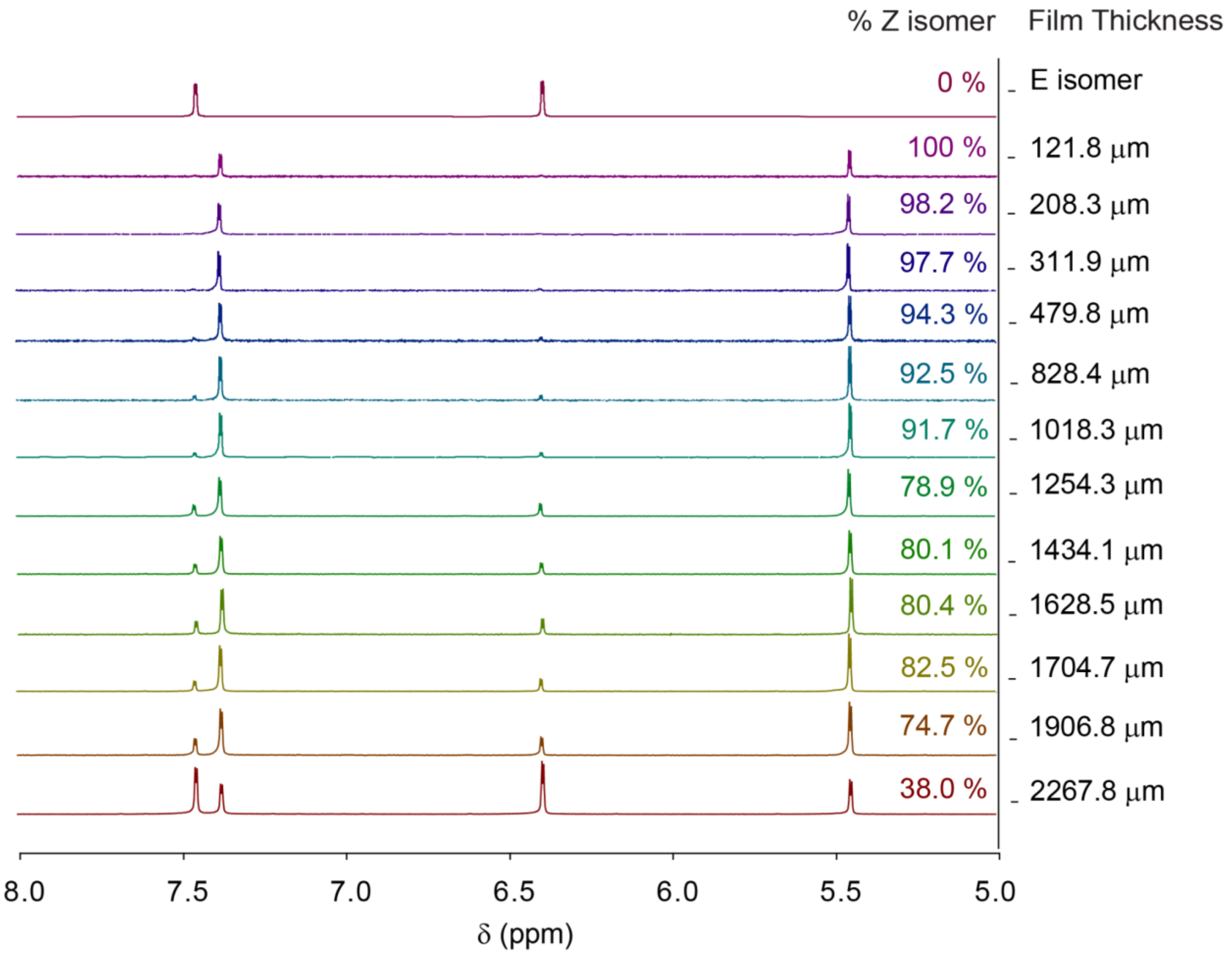

Fig. S28. The *Z* ratios of UV-irradiated samples of compound **2** at different thickness were determined by ^{1}H NMR. 24 hours of irradiation were applied.

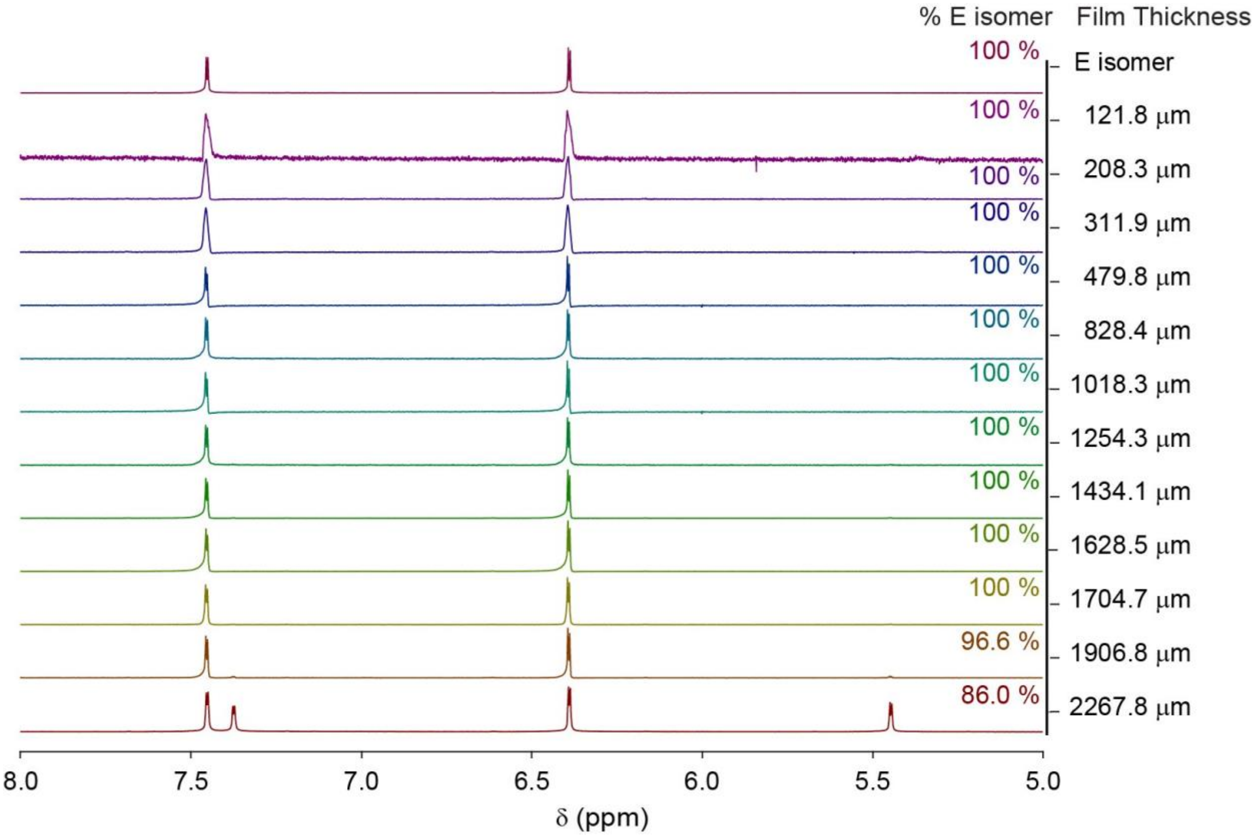

Fig. S29. The *E* ratios of 530 nm-irradiated samples of compound **2** at different thickness were determined by ^{1}H NMR. 5 hours of irradiation were applied.

12. Profilometer Measurements

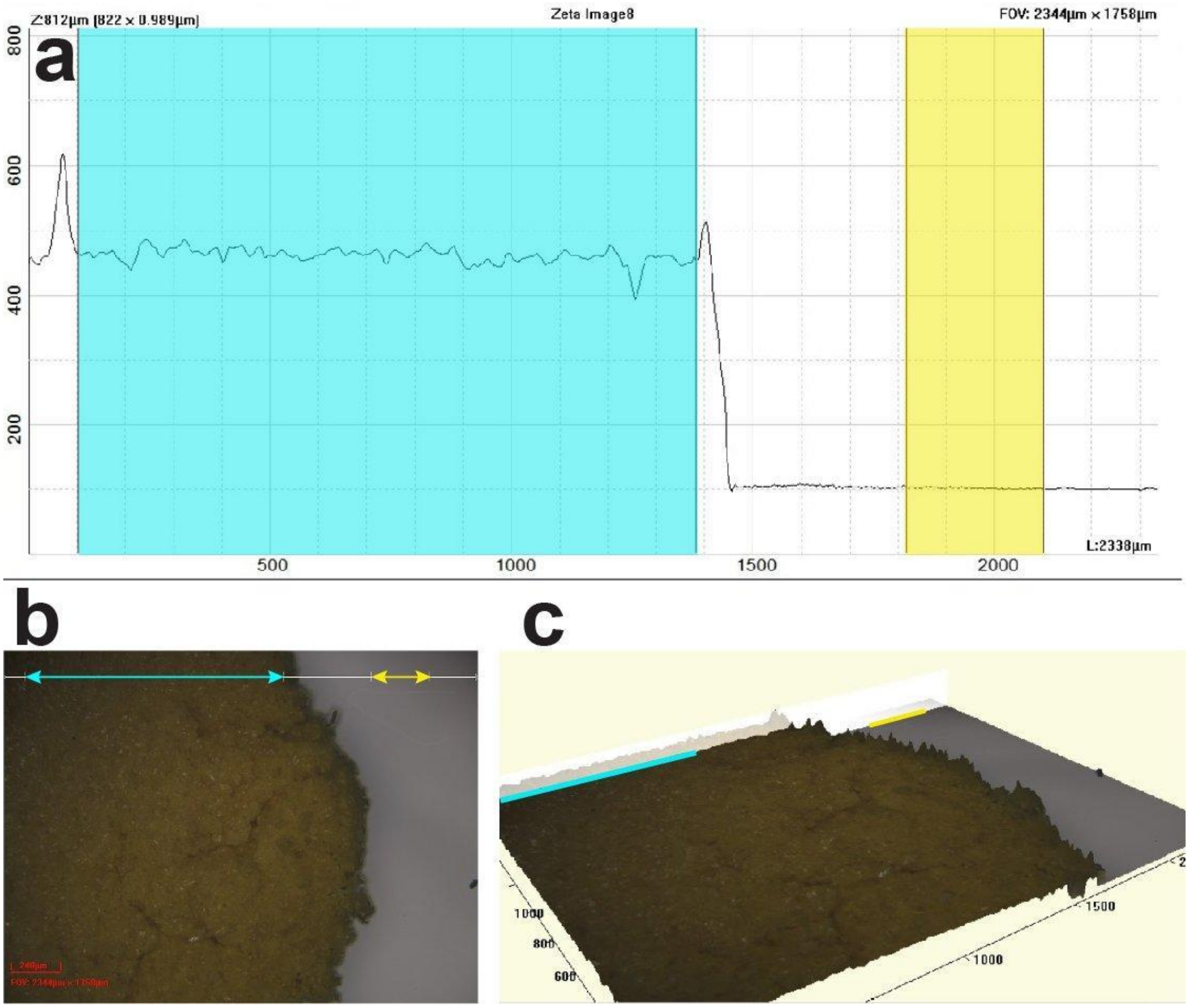

Fig. S30. a) Profile of **2-*E*** film with an average thickness of 425.8 µm using thin film. The thickness of the sample corresponds to the region in blue, and the yellow corresponds to the surface of the glass substrate. b) Optical microscope image of the measured area. c) 3D topography of the measured area.

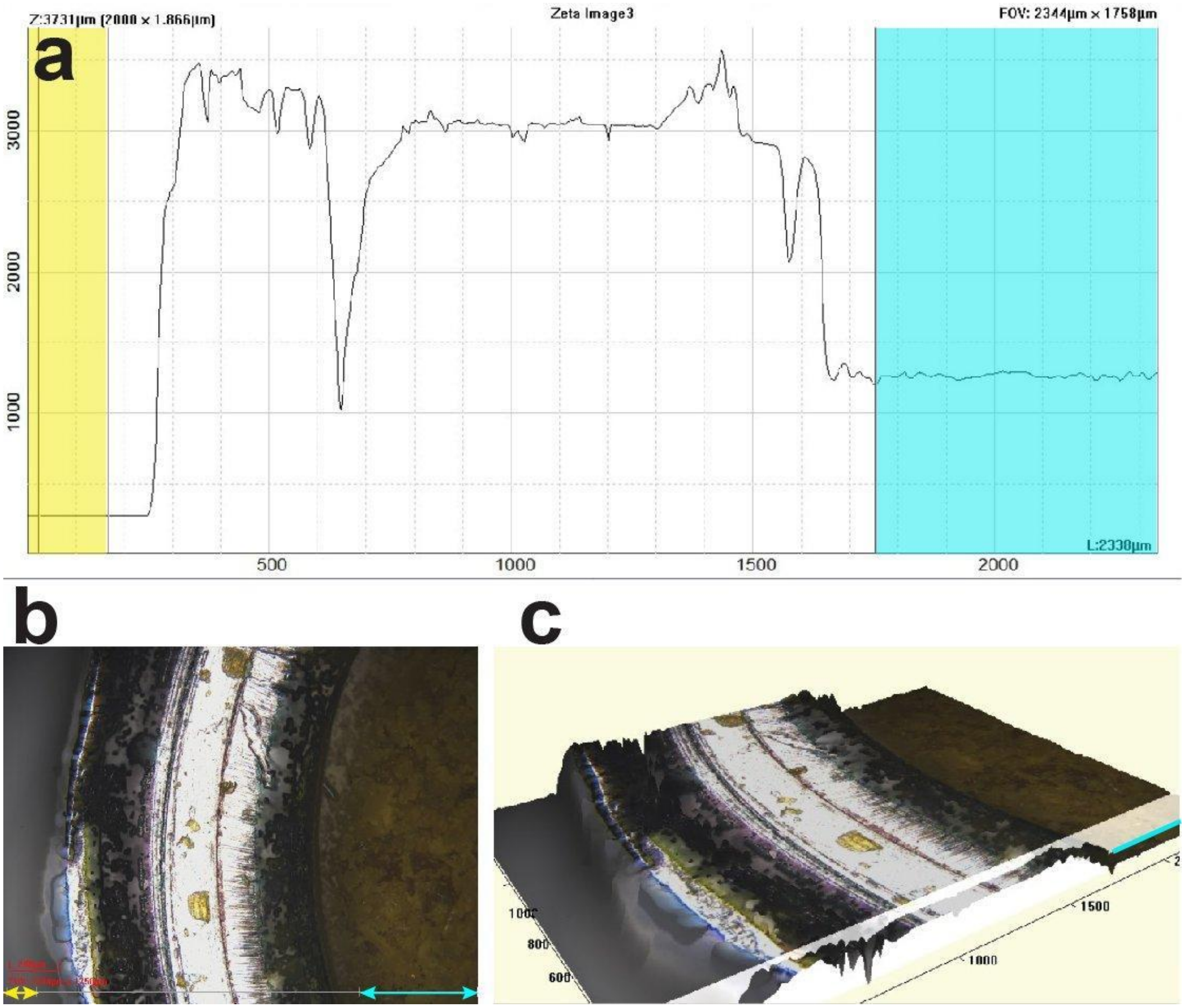

Fig. S31. a) Profile of **2-*E*** film with an average thickness of 479.8 µm using empty DSC pan. The thickness of both the sample and pan corresponds to the region in blue, and the yellow corresponds to the surface of the glass substrate. The measurements were taken as the difference between the surface containing compound and the surface of the substrate. b) Optical microscope image of the measured area. c) 3D topography of the measured area. See Supplementary Note 3 for how thicknesses were determined.

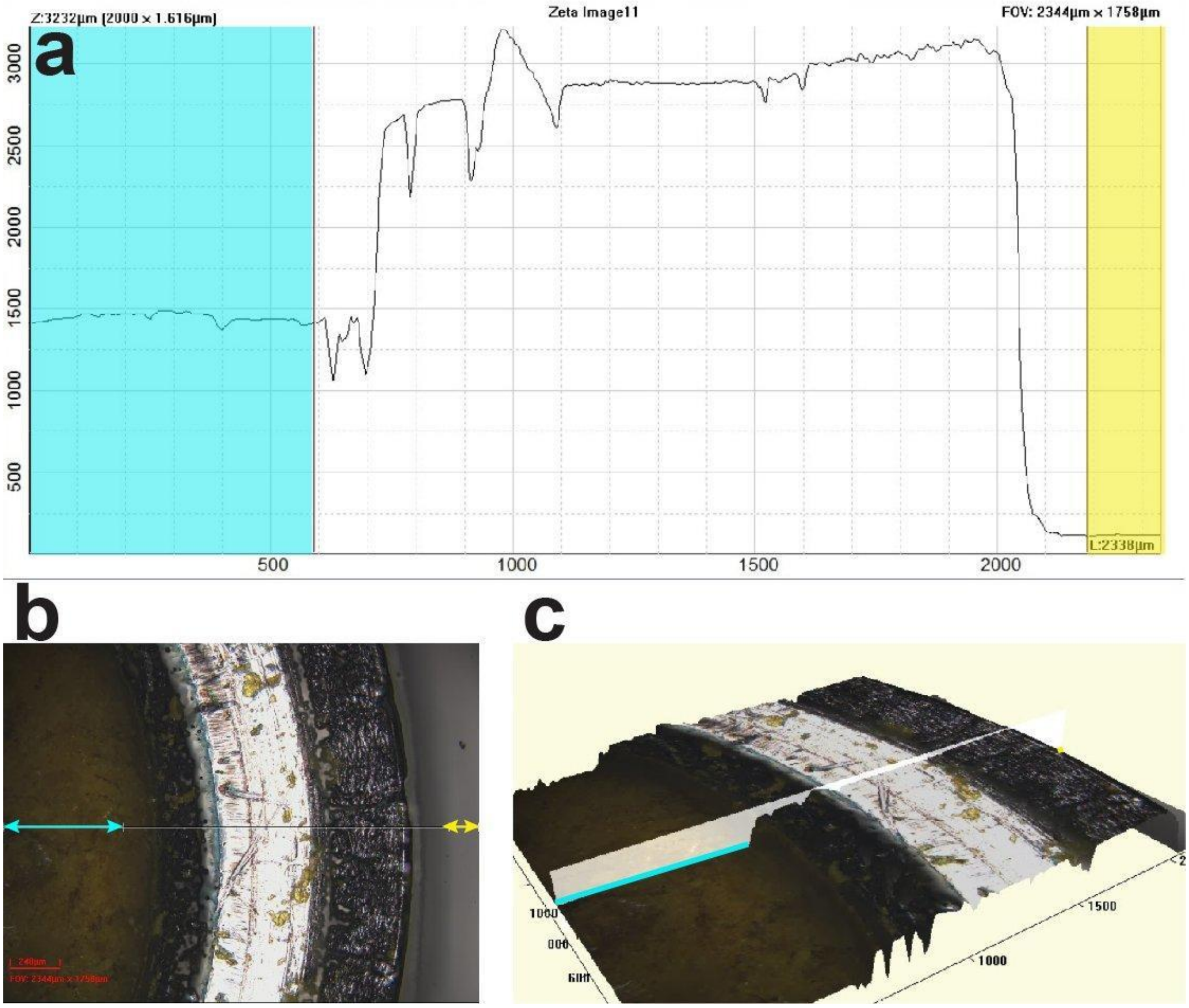

Fig. S32. a) Profile of **2-*E*** film with an average thickness of 828.4 µm using empty DSC pan. The thickness of both the sample and pan corresponds to the region in blue, and the yellow corresponds to the surface of the glass substrate. Measurements were taken as the difference between the surface containing compound and the surface of the substrate. b) Optical microscope image of the measured area. c) 3D topography of the measured area. See Supplementary Note 3 for how thicknesses were determined.

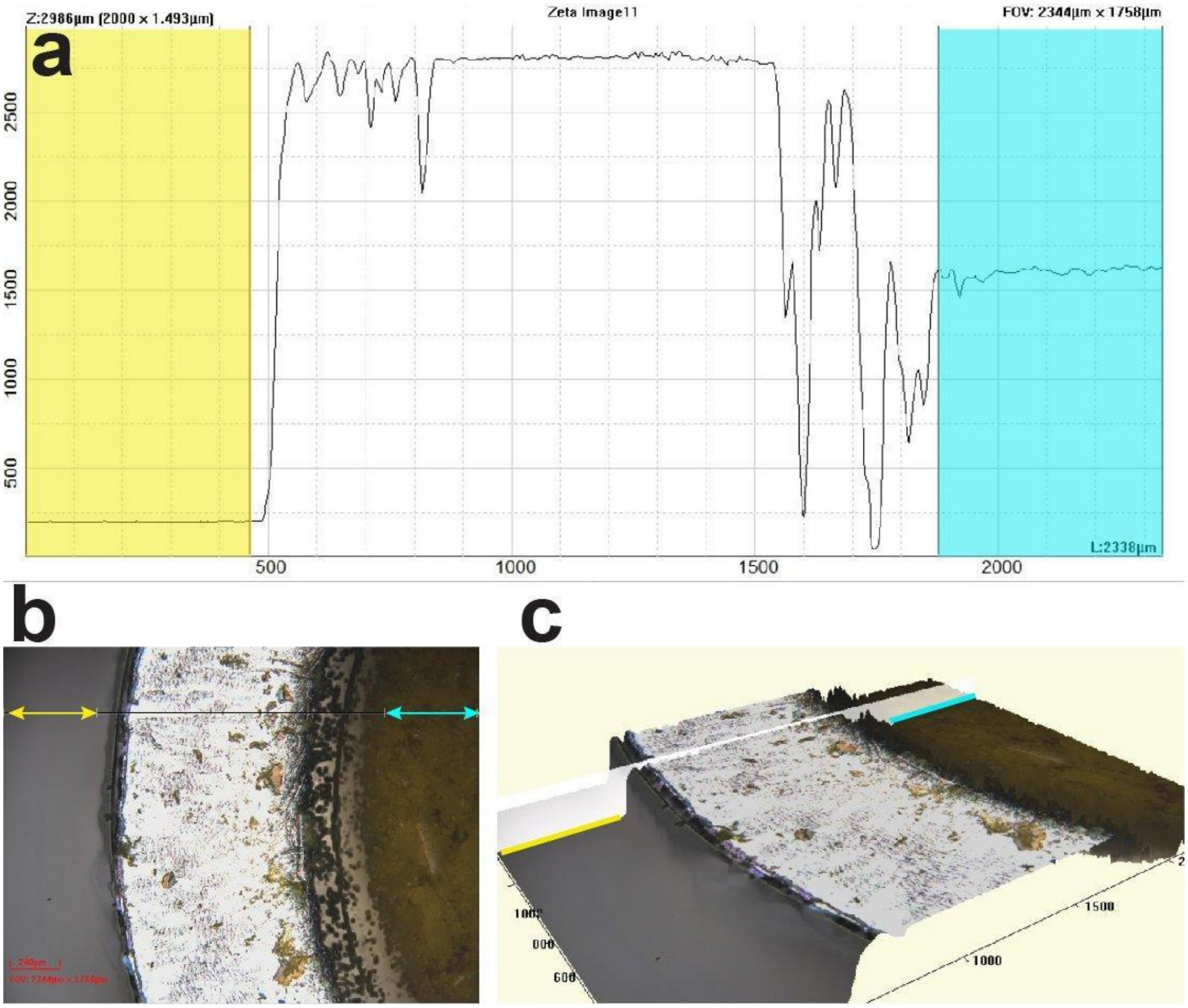

Fig. S33. a) Profile of **2-*E*** film with an average thickness of 1022.7 µm using empty DSC pan. The thickness of both the sample and pan corresponds to the region in blue, and the yellow corresponds to the surface of the glass substrate. The measurements were taken as the difference between the surface containing compound and the surface of the substrate. b) Optical microscope image of the measured area. c) 3D topography of the measured area. See Supplementary Note 3 for how thicknesses were determined.

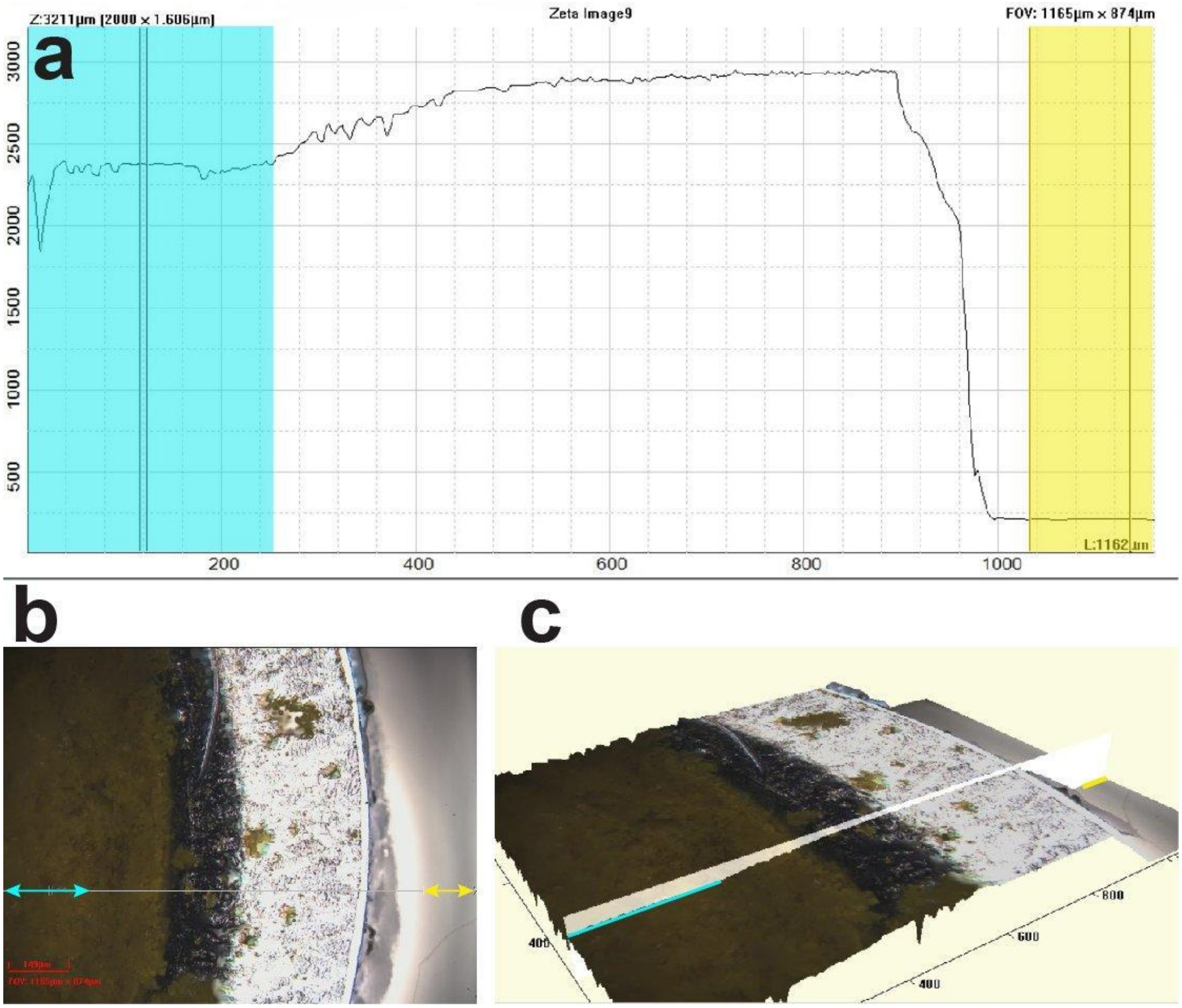

Fig. S34. a) Profile of **2**-***E*** film with an average thickness of 1906.8 µm using empty DSC pan. The thickness of both the sample and pan corresponds to the region in blue, and the yellow corresponds to the surface of the glass substrate. The measurements were taken as the difference between the surface containing compound and the surface of the substrate. b) Optical microscope image of the measured area. c) 3D topography of the measured area. See Supplementary Note 3 for how thicknesses were determined.

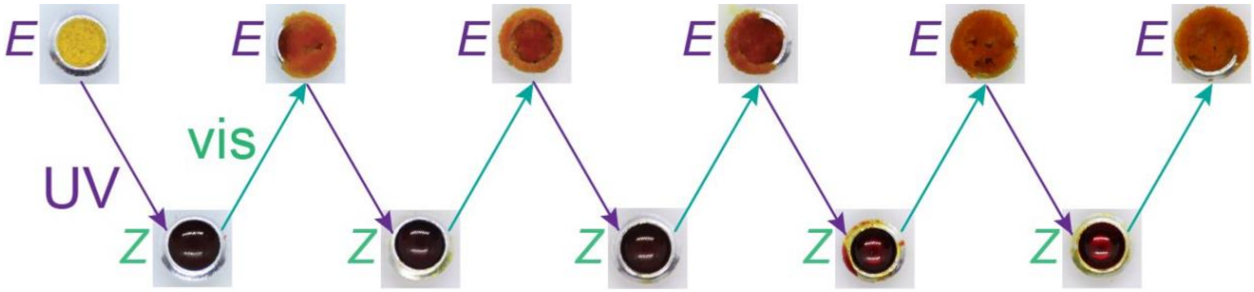

Fig. S35. Condensed-phase isomerization cycling of a 1704.7 μm film. Films were cycled five times between charging and discharging states, using 365 nm for 24 hours and 530 nm for 5 hours, respectively.

13. Bulk Sample Isomerization

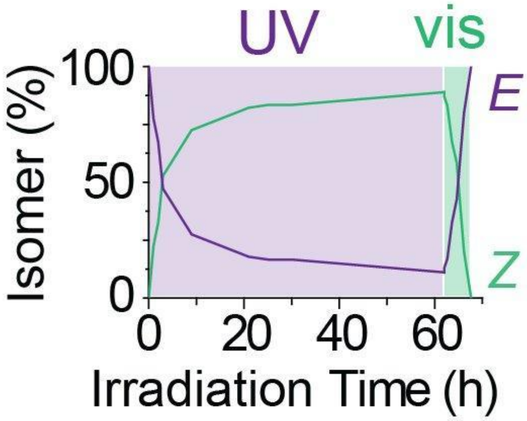

Fig. S36. The ratio between *E* and *Z* isomers changing during the UV and visible light irradiation in bulk experiment of **2**.

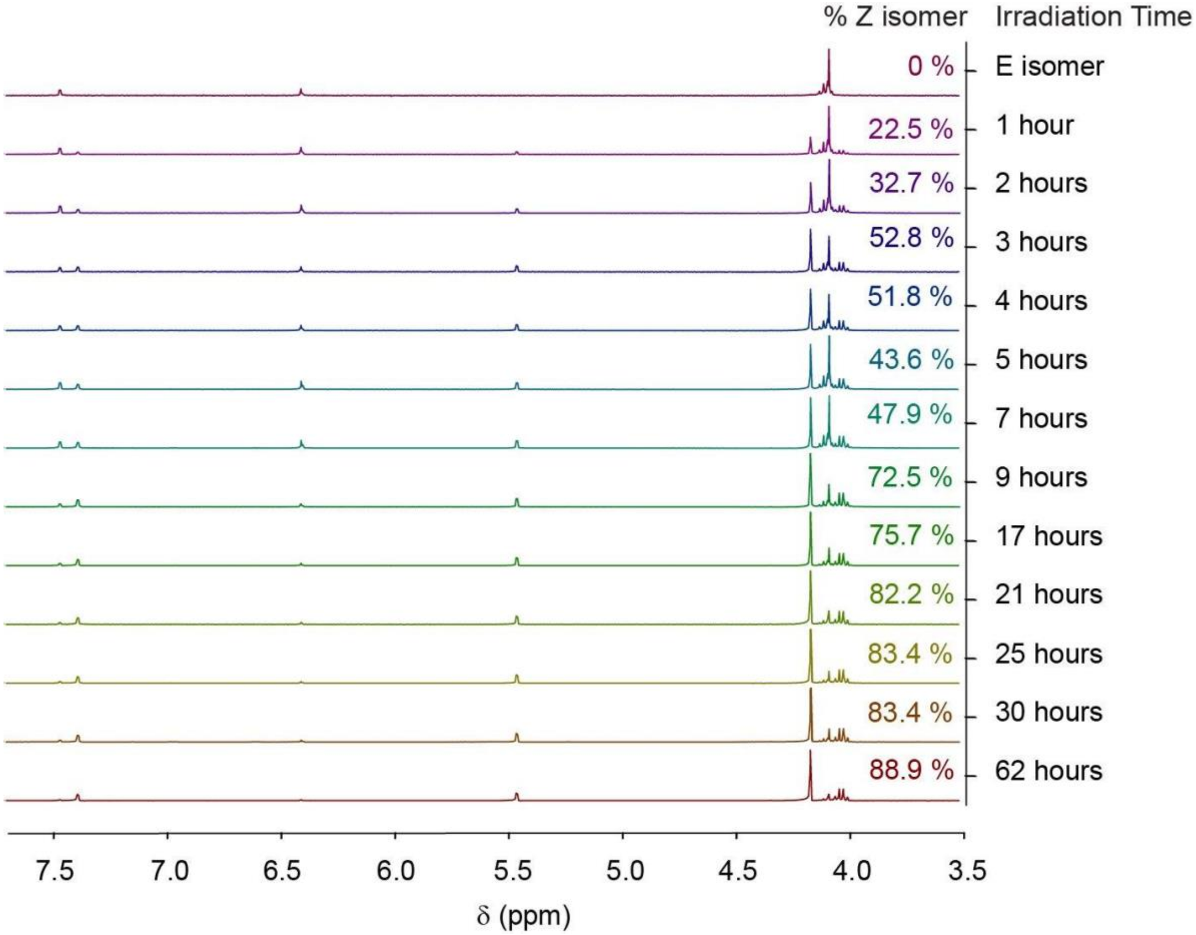

Fig. S37. The *Z* ratios were determined by ^{1}H NMR for the bulk charging of compound **2** at 115 mg scale.

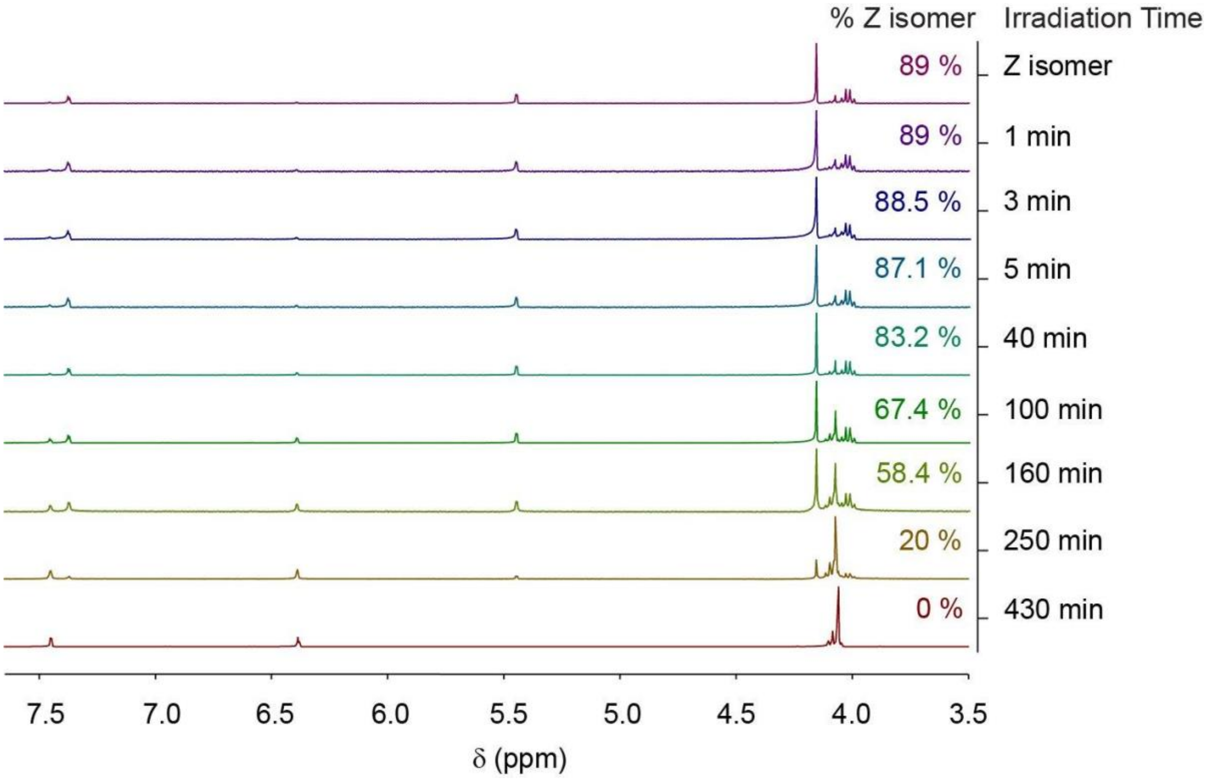

Fig. S38. The *Z* ratios of corresponding cuvettes were determined by ^{1}H NMR for the bulk discharging of compound **2** at 115 mg scale.

14. Additional Photoliquefaction Results

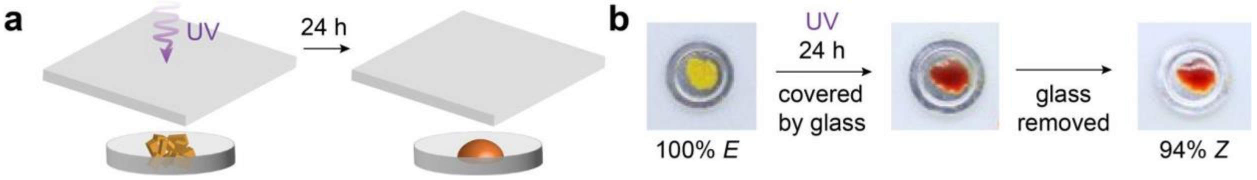

Fig. S39. A control experiment for the molecular convection test.

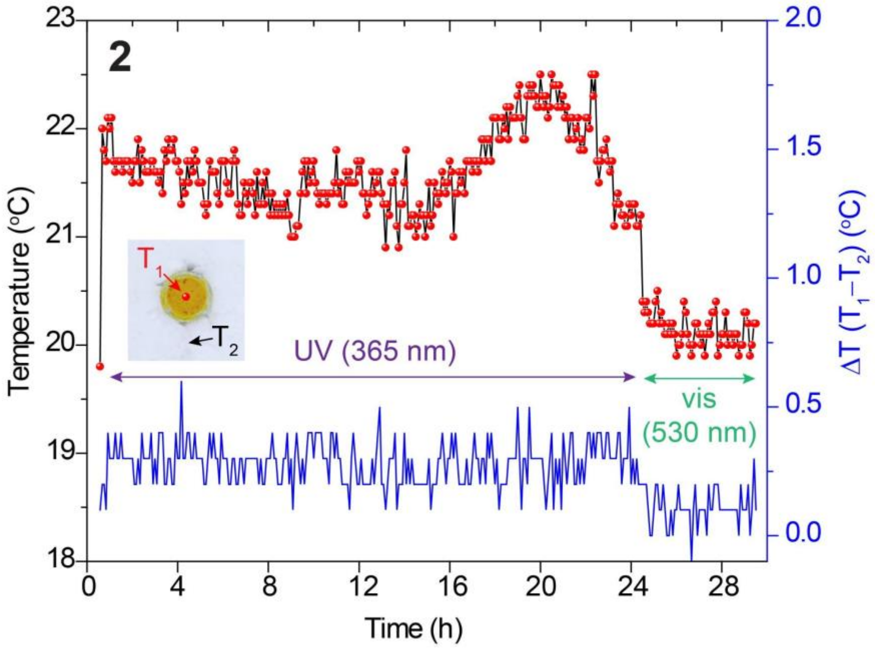

Fig. S40. IR camera experiment showing the photothermal effect of UV irradiation. T_1 is measured from the center of the sample, and T_2 is measured from the styrofoam substrate.

15. Crystal Structures

The X-ray Crystal Structure of 1

Crystal data for **1**: $C_{10}H_{14}N_6\cdot 2(H_2O)$, M = 254.30, monoclinic, $P2_1/c$ (no. 14), a = 7.2525(2), b = 25.3800(6), c = 7.3209(2) Å, β = 94.271(3)°, V = 1343.81(7) Å^3, Z = 4, D_c = 1.257 g cm^{-3}, μ(Cu-Kα) = 0.761 mm^{-1}, T = 173 K, pale yellow platy needles, Agilent Xcalibur PX Ultra A diffractometer; 2644 independent measured reflections (R_{int} = 0.0388), F^2 refinement,[8,9] R_1(obs) = 0.0445, wR_2(all) = 0.1301, 2016 independent observed absorption-corrected reflections [$|F_o| > 4\sigma(|F_o|)$, completeness to θ_{full}(67.7°) = 100%], 191 parameters. CCDC 2165362.

The O20- and O30-based included water molecules in the structure of **1** were both found to be involved in O–H···N hydrogen bonding, to N6 and N13 respectively. The hydrogen atoms involved in these interactions were located from Δ*F* maps and refined freely subject to O–H distance constraints of 0.90 Å. Additionally these two water molecules are also linked by O–H···O hydrogen bonds to each other across two independent centers of symmetry to form an extended ···O30···O20···O20···O30···O30···O20··· chain along the crystallographic *c* axis direction. As a consequence of the two independent centers of symmetry, the positions of the hydrogen atoms along this chain are inherently disordered, and four sites were located from Δ*F* maps and refined freely at 50% occupancy subject to O–H distance constraints of 0.90 Å.

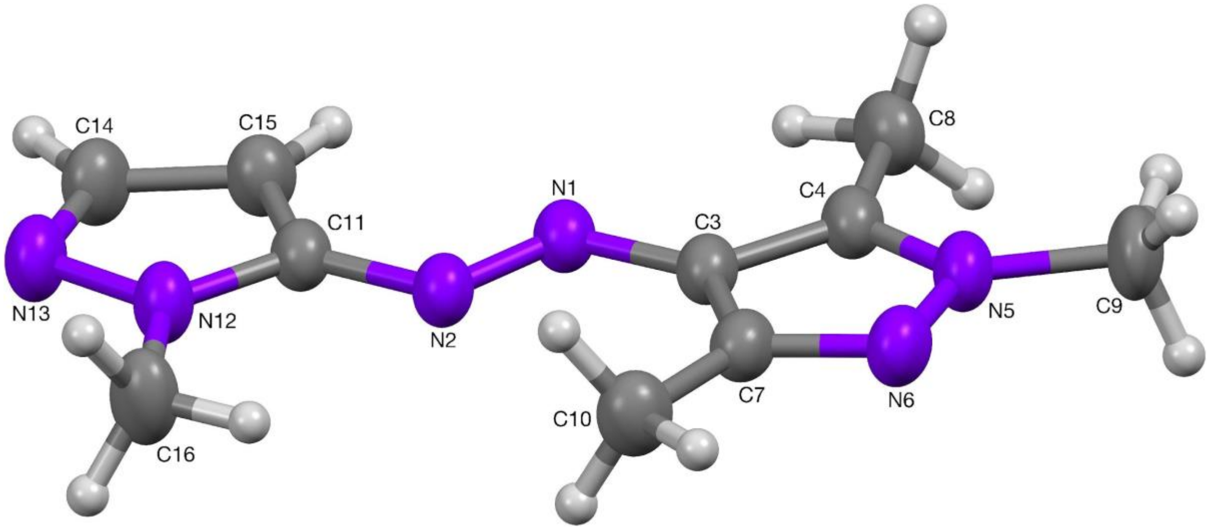

Fig. S41. The crystal structure of **1** (50% probability ellipsoids).

The X-ray Crystal Structure of 3

Crystal data for **3**: $C_{12}H_{18}N_6$, M = 246.32, orthorhombic, $P2_12_12_1$ (no. 19), a = 5.57769(13), b = 9.2160(2), c = 26.2480(7) Å, V = 1349.26(6) Å^3, Z = 4, D_c = 1.213 g cm^{-3}, μ(Cu-Kα) = 0.631 mm^{-1}, T = 173 K, yellow plates, Agilent Xcalibur PX Ultra A diffractometer; 2632 independent measured reflections (R_{int} = 0.0355), F^2 refinement,[8,9] R_1(obs) = 0.0363, wR_2(all) = 0.0935, 2324 independent observed absorption-corrected reflections [$|F_o| > 4\sigma(|F_o|)$, completeness to θ_{full}(67.7°) = 99.7%], 168 parameters. The absolute structure of **3** could not be determined [Flack parameter x = –0.1(3)]. CCDC 2165363.

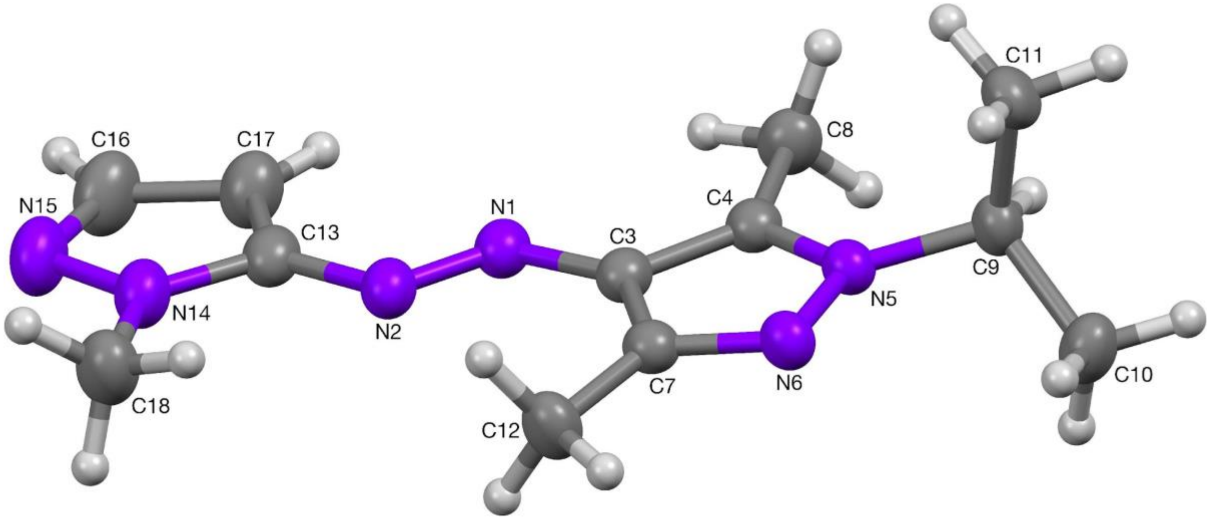

Fig. S42. The crystal structure of **3** (50% probability ellipsoids).

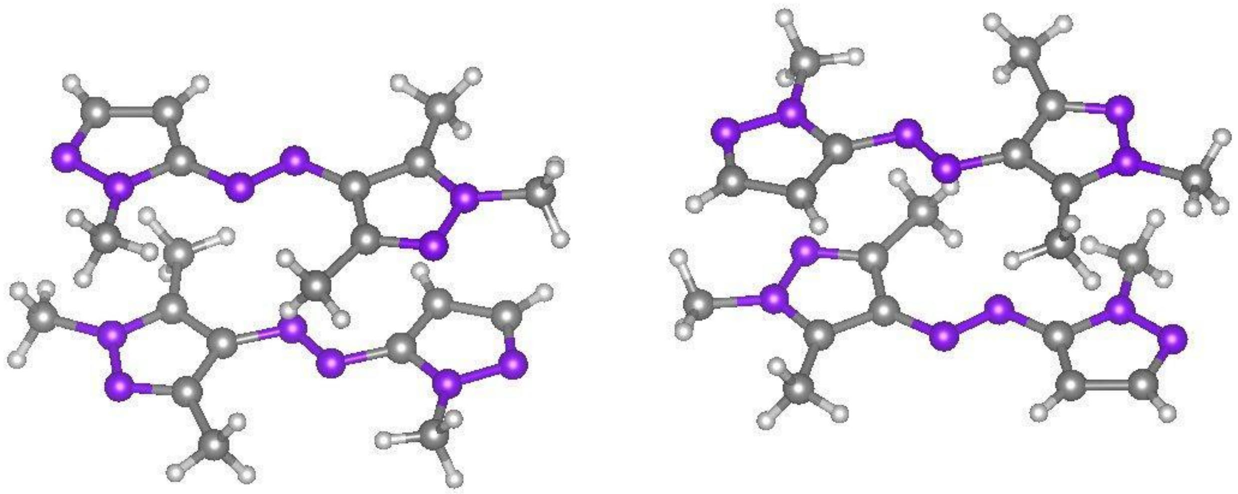

Fig. S43. The molecular packing structure of **1**.

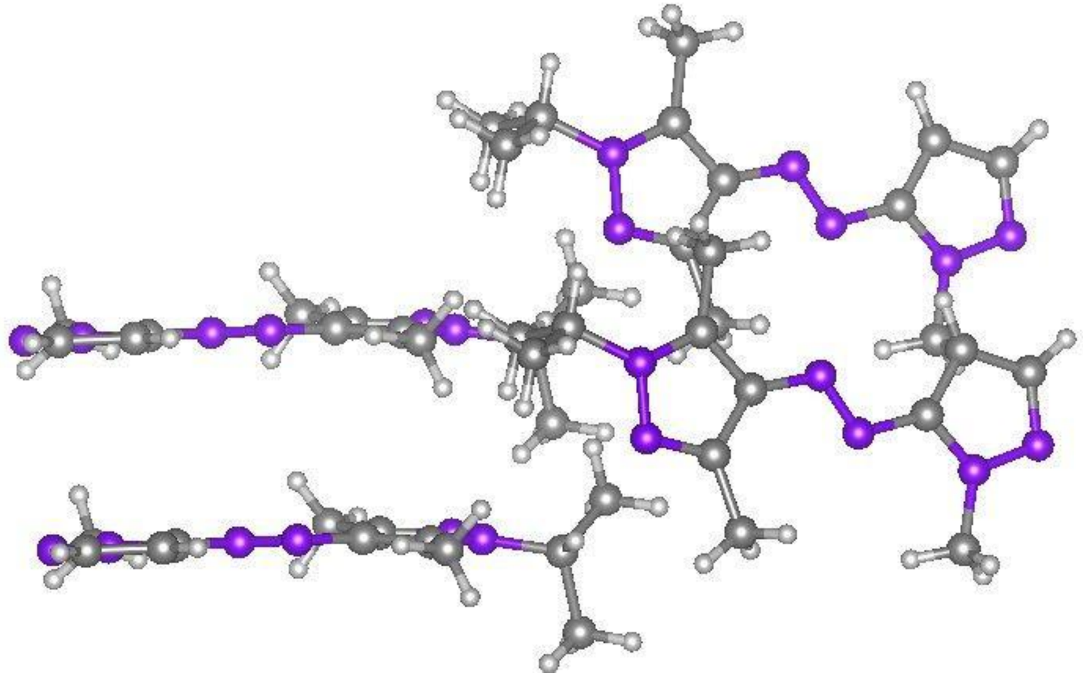

Fig. S44. The molecular packing structure of **3**.

16. Photoliquefaction of Arylazopyrazoles 4pzMe, 5pzH and 4pzH

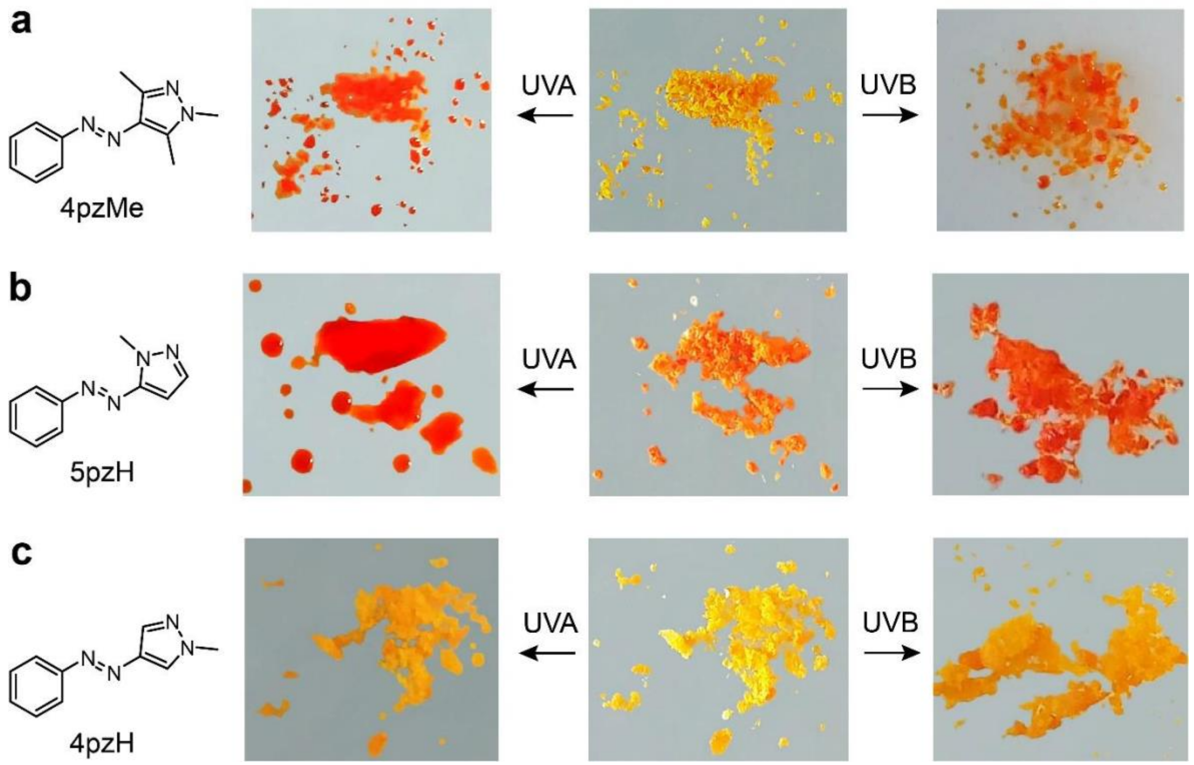

Fig. S45. Images demonstrating photoliquefaction, or lack thereof, of compounds a) **4pzMe** b) **5pzH** and c) **4pzH** when irradiated with UVA or UVB light for 24 hours.

17. References

1. Stranius, K.; Borjesson, K., Determining the Photoisomerization Quantum Yield of Photoswitchable Molecules in Solution and in the Solid State. *Sci. Rep.* **2017,** *7*, 41145.
2. https://www.luzchem.com/ExposureStandards.php accessed on 31 May 2022.
3. https://www.ledrise.eu/leds/nichia-smd-led-uv-ncsu276a-365nm-780mw-at-500ma-19w-emitter.html accessed on 20 June 2022.
4. https://www.ledrise.eu/leds/nichia-ncsg219b-v1-139lm-green-emitter.html accessed on 20 June 2022.
5. https://www.thorlabs.com/newgrouppage9.cfm?objectgroup_id=2692 accessed on 20 June 2022.
6. ASTM G173-03 (2020), Standard Tables for Reference Solar Spectral Irradiances: Direct Normal and Hemispherical on 37° Tilted Surface, ASTM International, West Conshohocken, PA, 2020, www.astm.org
7. Han, G. G. D.; Li, H.; Grossman, J. C., Optically-Controlled Long-Term Storage and Release of Thermal Energy in Phase-Change Materials. *Nat. Commun.* **2017**, *8*, 1446.
8. SHELXTL v5.1, Bruker AXS, Madison, WI, 1998.
9. SHELX-2013, G.M. Sheldrick, *Acta Cryst.*, 2015, **C71**, 3-8.
